# A Body Shape Index (ABSI), hip index, and risk of cancer in the UK Biobank cohort

**DOI:** 10.1002/cam4.4097

**Published:** 2021-07-01

**Authors:** Sofia Christakoudi, Konstantinos K. Tsilidis, Evangelos Evangelou, Elio Riboli

**Affiliations:** ^1^ Department of Epidemiology and Biostatistics School of Public Health Imperial College London Norfolk Place, London UK; ^2^ MRC Centre for Transplantation King’s College London London UK; ^3^ Department of Hygiene and Epidemiology University of Ioannina School of Medicine Ioannina Greece

**Keywords:** cancer prevention, cancer risk factors, epidemiology, risk assessment

## Abstract

Abdominal size is associated positively with the risk of some cancers but the influence of body mass index (BMI) and gluteofemoral size is unclear because waist and hip circumference are strongly correlated with BMI. We examined associations of 33 cancers with A Body Shape Index (ABSI) and hip index (HI), which are independent of BMI by design, and compared these with waist and hip circumference, using multivariable Cox proportional hazards models in UK Biobank. During a mean follow‐up of 7 years, 14,682 incident cancers were ascertained in 200,289 men and 12,965 cancers in 230,326 women. In men, ABSI was associated positively with cancers of the head and neck (hazard ratio HR = 1.14; 95% confidence interval 1.03–1.26 per one standard deviation increment), esophagus (adenocarcinoma, HR = 1.27; 1.12–1.44), gastric cardia (HR = 1.31; 1.07–1.61), colon (HR = 1.18; 1.10–1.26), rectum (HR = 1.13; 1.04–1.22), lung (adenocarcinoma, HR = 1.16; 1.03–1.30; squamous cell carcinoma [SCC], HR = 1.33; 1.17–1.52), and bladder (HR = 1.15; 1.04–1.27), while HI was associated inversely with cancers of the esophagus (adenocarcinoma, HR = 0.89; 0.79–1.00), gastric cardia (HR = 0.79; 0.65–0.96), colon (HR = 0.92; 0.86–0.98), liver (HR = 0.86; 0.75–0.98), and multiple myeloma (HR = 0.86; 0.75–1.00). In women, ABSI was associated positively with cancers of the head and neck (HR = 1.27; 1.10–1.48), esophagus (SCC, HR = 1.37; 1.07–1.76), colon (HR = 1.08; 1.01–1.16), lung (adenocarcinoma, HR = 1.17; 1.06–1.29; SCC, HR = 1.40; 1.20–1.63; small cell, HR = 1.39; 1.14–1.69), kidney (clear‐cell, HR = 1.25; 1.03–1.50), and post‐menopausal endometrium (HR = 1.11; 1.02–1.20), while HI was associated inversely with skin SCC (HR = 0.91; 0.83–0.99), post‐menopausal kidney cancer (HR = 0.77; 0.67–0.88), and post‐menopausal melanoma (HR = 0.90; 0.83–0.98). Unusually, ABSI was associated inversely with melanoma in men (HR = 0.89; 0.82–0.96) and pre‐menopausal women (HR = 0.77; 0.65–0.91). Waist and hip circumference reflected associations with BMI, when examined individually, and provided biased risk estimates, when combined with BMI. In conclusion, preferential positive associations of ABSI or inverse of HI with several major cancers indicate an important role of factors determining body shape in cancer development.

## Graphical abstract

Using allometric anthropometric body‐shape indices, which are independent of body mass index (BMI) by design, we have confirmed positive associations with waist size and have additionally provided evidence for inverse associations with hip size for a range of cancers, extending beyond obesity‐related cancers. We have demonstrated that the traditional anthropometric indices waist and hip circumference are influenced by strong associations of BMI with cancer risk and can give biased risk estimates when combined with BMI.

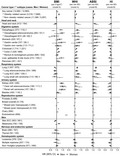

## 1 INTRODUCTION

General obesity, evaluated with body mass index (BMI), is an acknowledged risk factor for cancer.^1^ The metabolic complications of obesity, however, are differentially associated with body shape: positively with abdominal size and inversely with gluteofemoral size.^2^ While visceral obesity has been associated with higher risk of several cancers,^3^ less is known about gluteofemoral size. Further, waist (WC) and hip circumference (HC), traditionally used as body‐shape measures, are strongly correlated with BMI,^4^ questioning the independence from BMI of their associations. Although the waist‐to‐hip ratio (WHR) is correlated only moderately with BMI, it cannot differentiate abdominal from gluteofemoral size.

To account for the strong correlation of WC and HC with BMI, A Body Shape Index (ABSI) and hip index (HI) were designed as body shape indices independent from BMI.^5^, ^6^ In analogy to BMI, ABSI and HI are based on the principle of allometry, scaling the epansion of regional body compartments to the increase of total body size.^7^ ABSI and HI are relative measures of body size, while WC and HC are absolute measures. Although ABSI and WC reflect abdominal obesity more specifically than BMI and neither is influenced by lean mass, WC simply reflects the abdominal dimensions, which would increase when excess fat accumulates, but does not provide information whether the excess fat is distributed evenly across the body or accumulates preferentially in the abdominal area, which is achieved by ABSI. WC defines abdominal obesity in terms of absolute waist size, which increases in parallel to the total body size. Thus, WC identifies with abdominal obesity only those among normal‐weight individuals that have more extreme abdominal dimensions and classifies most obese individuals as having abdominal obesity.^4^ ABSI, however, defines abdominal obesity independently of the total body size, in comparison to an average individual with the same weight and height. A similar logic applies to HC and HI, but there is a further complication that lean mass has a larger contribution to hip than to waist size. Although associations of ABSI and HI with mortality and cardio‐metabolic risk factors have been examined in more detail,^4^, ^5^, ^8^ there are no studies to date systematically examining associations with cancer risk.

Using data from UK Biobank, we examined associations between combinations of allometric body shape indices and BMI and the risk of cancer development in the most common tumor sites and the major morphologies and contrasted these with traditional body shape indices.

## 2 METHODS

### 2.1 Study population

UK Biobank is a prospective population‐based cohort, comprising half a million participants aged 40–70 years at baseline, recruited between 2006 and 2010.^9^, ^10^ We excluded 71,873 participants with prevalent cancer at baseline, missing or extreme anthropometric measurements, hysterectomy (for endometrial cancer) or bilateral oophorectomy (for ovarian cancer) at baseline and restricted the study to participants with self‐reported white ancestry, as ethnic variations in body shape are large and other ethnic groups were limited (Figure S1).

### 2.2 Cancer ascertainment

Cancer cases were ascertained based on linkage to the national cancer registry of the United Kingdom. The outcome of interest was first primary cancer diagnosed after baseline, which was defined with codes C00–C99 from the 10th version of the International Statistical Classification of Diseases (ICD10) and malignant behavior with behavioral code 3 (malignant, primary site, *n* = 27,596) or 5 (malignant, microinvasive, *n* = 51). Non‐melanoma skin cancers were not considered cancer cases, except for skin squamous cell carcinomas (SCC, defined with ICD10 code C44 and morphological codes 8070, 8071, 8072 or 8083). Participants with first cancer with ICD10 code starting with “C” and metastatic or unknown behavior with behavioral codes 6 (malignant, metastatic site), 9 (malignant, uncertain whether primary or metastatic site) or missing (*n* = 286), were censored at the date of diagnosis. A limited number of cancer registry entries with ICD10 code starting with “C” had non‐malignant behavioral codes 0 “Benign,” 1 “Uncertain whether benign or malignant” or 2 “Carcinoma in situ” (*n* = 63). These were not considered cancer cases and for them, follow‐up continued until experiencing a malignant event, as defined above, death or administrative censoring. Similar rules were applied to the definition of prevalent cancers at baseline, but for them, some entries were coded according to the 9th version of ICD (see Supplementary Methods for further details). Follow‐up was censored for all participants remaining cancer‐free at 31st March 2016 (last complete cancer registry information, including *n* = 383 first primary cancers diagnosed after this date), or at the date of death, if earlier. Cancer types and subtypes are defined in Supplementary Methods, in accordance with our previous report.^11^ Obesity‐related cancers included oesophageal adenocarcinoma, cancers of the gastric cardia, colon, rectum and rectosigmoid junction, liver, gallbladder and bile ducts, pancreas, kidney, postmenopausal breast, ovary, endometrium, thyroid and multiple myeloma, for which the International Agency for Research on Cancer has acknowledged associations with obesity.^1^ Non‐obesity‐related cancers included the remaining cancers.

### 2.3 Anthropometric indices

Anthropometric measurements were assessed by trained technicians, following established protocols.^9^ Waist circumference was measured at the natural indent or the umbilicus and hip circumference at the widest point. We calculated ABSI and HI with coefficients from the National Health and Nutrition Examination Survey (NHANES)^5^, ^6^ and waist‐to‐hip index (WHI), the allometric counterpart of WHR, with simple‐fraction coefficients derived from UK Biobank data (Supplementary Methods):

$$\text{ABSI}=\text{WC}\left(\text{mm}\right)*\text{Weight}{\left(\text{kg}\right)}^{‐2/3}*\text{Height}{\left(m\right)}^{5/6}$$

$$\text{HI}=\text{HC}\left(\text{cm}\right)*\text{Weight}{\left(\text{kg}\right)}^{‐0.482}*\text{Height}{\left(\text{cm}\right)}^{0.310}$$

$$\text{WHI}=\text{WHR}*\text{Weight}{\left(\text{kg}\right)}^{‐1/4}*\text{Height}{\left(\text{cm}\right)}^{1/2}$$

$$\text{BMI}=\text{Weight}\left(\text{kg}\right)*\text{Height}{\left(m\right)}^{‐2}$$

### 2.4 Statistical analysis

In the main analyses, we used obesity indices as continuous variables. For body shape indices, we used a standardized scale in sex‐specific *z*‐scores (observed value minus mean, divided by standard deviation, SD). In secondary analyses, we examined the consistency of our findings with categorical variables, using sex‐specific tertiles of body shape indices and World Health Organization categories of BMI. Categorical variables provided some information for potentially nonlinear associations. As visceral fat increases when estrogens decline after the menopause^12^ and some cancers have been associated with abdominal obesity only in post‐menopausal women,^3^ we examined heterogeneity by menopausal status.

We estimated hazard ratios (HR) and 95% confidence intervals (CI) with delayed‐entry Cox proportional hazards models, conditional on cancer‐free survival to cohort entry. The underlying time scale was age, starting at the date of birth. Entry time was the date of attending an assessment center at baseline. Exit time was the date of diagnosis of the first incident cancer, or death, or last complete follow‐up, whichever occurred first. We examined associations with general and regional body size, independent of each other, with two combinations of obesity indices: BMI and WHI (Model A) and BMI, ABSI and HI (Model B). We interpreted HR estimates for continuous variables as the risk per one SD increment for body shape indices or per 5 kg/m^2^ increment for BMI. The reference category for categorical variables was the lowest tertile for body shape indices or the normal‐weight category for BMI. As body shape and the risk of some cancers show pronounced sexual dimorphisms,^13^, ^14^ we examined men and women separately.

All models were stratified by age at baseline and region of the assessment center and adjusted for major risk factors for cancer and obesity and potential confounders: height, weight change during the year preceding baseline (indicator of weight dynamics), Townsend deprivation index (indicator of socio‐economic status), smoking status, alcohol consumption, physical activity, consumption of fresh fruit and vegetables, processed and red meat, family history of cancer, in women also menopausal status, age at last live birth (indicator of reproductive history), use of hormone replacement therapy and oral contraceptives and, for skin SCC and melanoma, sun‐exposure‐related factors (skin color, skin tanning, hair color, childhood sunburns, solarium use, sun/UV protection and time outdoors in summer) (categories are defined in Supplementary Methods). We replaced missing values for covariates with the sex‐specific median category (when missingness was <5%) but created missing categories for childhood sunburns (≈25%) and time outdoors (≈5%). Tests of statistical significance were two‐sided. *p*‐values (marked in all Supplementary Tables) provided guiding information for the strength of the available evidence, with *p *< 0.05 considered weaker evidence and *p *< 0.001 stronger evidence (the latter would correspond to Bonferroni correction for 50 comparisons). We did not formally penalize *p*‐values for multiple comparisons, as we were not addressing the general question of whether body shape is associated with cancer overall. We consider individual cancers as separate outcomes and, hence, the comparisons for each specific cancer were limited. We focused the description of our results on HR estimates with 95% confidence intervals and graphical representations, which are more informative than the corresponding *p*‐value and additionally provide an indication of the statistical power of the analysis and an estimate of effect size.^15^ We tested statistical interactions with the Wald test for the interaction term between menopausal status at baseline and each obesity index (continuous scale), included (one at a time) in the fully adjusted model for women (*p*_interaction_).

In sensitivity analyses, we excluded participants with less than 2 years of follow‐up, to explore possible reverse causality. To examine the influence of covariates, we derived unadjusted HR estimates for combinations of obesity indices (with stratification by age and region), adjusted only for smoking status, and omitted female‐specific factors (women) or sun‐exposure‐related factors (skin cancers). To check for residual associations with BMI when calculating ABSI and HI with coefficients from NHANES, we derived coefficients from UK Biobank data (Supplementary Methods). To illustrate differences with traditional body shape indices, we examined these individually and in combination with BMI, with stratification and adjustment as the main models.

We used R version 3.6.1^16^ for data management and STATA‐13^17^ for statistical analyses.

## 3 RESULTS

### 3.1 Cohort characteristics

During a mean follow‐up of 7 years, 14,682 incident cancers were ascertained in 200,289 men and 12,965 cancers in 230,326 women. Men had larger WC, ABSI, WHR, and WHI, but similar HC and smaller HI compared to women and were less likely to have a healthy lifestyle regarding smoking, alcohol consumption or diet (Table 1). While a comparable proportion of men and women were obese (with BMI ≥ 30 to < 45 kg/m^2^), a larger proportion of men were overweight (with BMI ≥ 25 to < 30 kg/m^2^) but a smaller proportion had recently gained weight at baseline. Pre‐menopausal women had lower BMI and ABSI compared to post‐menopausal women, but higher use of oral contraceptives and older age at reproduction (Table S1).

**TABLE 1:** Characteristics of study participants

Characteristics	Men	Women	Pre‐menopausal	Post‐menopausal
Cohort size: *n* (%)	200,289 (46.5)	230,326 (53.5)	67,106 (29.1)	163,220 (70.9)
Cancer cases: *n* (%)	14,682 (53.1)	12,965 (46.9)	2341 (18.1)	10,624 (81.9)
Age at baseline (years): mean (SD)	57.2 (8.1)	56.8 (8.0)	47.2 (3.9)	60.8 (5.4)
Follow‐up time (years): mean (SD)	6.9 (1.4)	7.0 (1.3)	7.1 (1.1)	6.9 (1.3)
Body size measures/indices: mean (SD)				
Height (cm)	175.9 (6.8)	162.6 (6.2)	164.2 (6.2)	162.0 (6.1)
Weight (kg)	86.1 (13.8)	71.2 (13.1)	71.2 (13.7)	71.2 (12.9)
Body mass index (BMI) (kg/m^2^)	27.8 (4.0)	26.9 (4.8)	26.4 (4.9)	27.1 (4.7)
BMI WHO category: *n* (%)				
Normal weight: BMI ≥18.5 to <25 kg/m^2^	49,992 (25.0)	92,272 (40.1)	31,261 (46.6)	61,011 (37.4)
Overweight: BMI ≥25 to <30 kg/m^2^	99,591 (49.7)	85,748 (37.2)	22,117 (33.0)	63,631 (39.0)
Obese: BMI ≥30 to <45 kg/m^2^	50,706 (25.3)	52,306 (22.7)	13,728 (20.5)	38,578 (23.6)
Traditional body shape indices: mean (SD)				
Waist circumference (cm)	96.9 (11.0)	84.4 (12.0)	82.3 (11.9)	85.2 (11.9)
Hip circumference (cm)	103.4 (7.2)	103.2 (9.7)	102.6 (9.8)	103.4 (9.7)
Waist‐to‐hip ratio (WHR)	0.94 (0.06)	0.82 (0.07)	0.80 (0.07)	0.82 (0.07)
Allometric body shape indices: mean(SD)				
A Body Shape Index (ABSI)	79.8 (4.1)	73.8 (5.0)	72.6 (4.7)	74.3 (5.0)
Hip Index (HI)	60.2 (2.2)	64.2 (2.5)	64.1 (2.4)	64.3 (2.5)
Waist‐to‐Hip Index (WHI)	4.08 (0.22)	3.59 (0.27)	3.54 (0.26)	3.61 (0.27)
Weight change during last year: *n* (%)				
Weight loss	29,050 (14.5)	34,959 (15.2)	10,569 (15.7)	24,390 (14.9)
Stable weight	123,137 (61.5)	117,071 (50.8)	32,237 (48.0)	84,834 (52.0)
Weight gain	44,687 (22.3)	74,689 (32.4)	23,191 (34.6)	51,498 (31.6)
Missing	3415 (1.7)	3607 (1.6)	1109 (1.7)	2498 (1.5)
Townsend deprivation index: median (IQR)				
Lowest tertile	−4.18 (1.03)	−4.17 (1.03)	−4.18 (1.06)	−4.16 (1.02)
Middle tertile	−2.26 (1.14)	−2.27 (1.09)	−2.25 (1.11)	−2.29 (1.09)
Highest tertile	1.74 (3.34)	1.51 (3.19)	1.58 (3.20)	1.48 (3.19)
Missing: *n* (%)	243 (0.1)	255 (0.1)	96 (0.1)	159 (0.1)
Smoking status: *n* (%)				
Never smoked	68,545 (34.2)	100,586 (43.7)	30,871 (46.0)	69,715 (42.7)
Former occasional smoker	50,609 (25.3)	63,068 (27.4)	18,941 (28.2)	44,127 (27.0)
Former regular smoker	55,968 (27.9)	45,621 (19.8)	10,084 (15.0)	35,537 (21.8)
Current smoker	24,504 (12.2)	20,317 (8.8)	7068 (10.5)	13,249 (8.1)
Missing	663 (0.3)	734 (0.3)	142 (0.2)	592 (0.4)
Alcohol consumption: *n* (%)				
Up to 3 times a month	40,795 (20.4)	80,744 (35.1)	21,708 (32.3)	59,036 (36.2)
Up to four times a week	106,892 (53.4)	110,897 (48.1)	35,681 (53.2)	75,216 (46.1)
Daily or almost daily	52,445 (26.2)	38,540 (16.7)	9672 (14.4)	28,868 (17.7)
Missing	157 (0.1)	145 (0.1)	45 (0.1)	100 (0.1)
Physical activity: *n* (%)				
Less active	30,530 (15.2)	38,492 (16.7)	11,345 (16.9)	27,147 (16.6)
Moderately active	89,789 (44.8)	120,059 (52.1)	31,673 (47.2)	88,386 (54.2)
Very active	79,323 (39.6)	70,936 (30.8)	23,948 (35.7)	46,988 (28.8)
Missing	647 (0.3)	839 (0.4)	140 (0.2)	699 (0.4)
Family history of cancer: *n* (%)				
No	130,121 (65.0)	147,988 (64.3)	47,166 (70.3)	100,822 (61.8)
Yes (lung, breast, prostate, bowel)	70,168 (35.0)	82,338 (35.7)	19,940 (29.7)	62,398 (38.2)
Fresh fruit and vegetable intake: *n* (%)				
Less than five portions a day	133,561 (66.7)	122,894 (53.4)	39,633 (59.1)	83,261 (51.0)
Five or more portions a day	63,329 (31.6)	105,014 (45.6)	26,969 (40.2)	78,045 (47.8)
Missing	3399 (1.7)	2418 (1.0)	504 (0.8)	1914 (1.2)
Processed meat intake: *n* (%)				
Less than twice a week	111,678 (55.8)	181,903 (79.0)	51,430 (76.6)	130,473 (79.9)
Twice or more a week	88,300 (44.1)	48,114 (20.9)	15,584 (23.2)	32,530 (19.9)
Missing	311 (0.2)	309 (0.1)	92 (0.1)	217 (0.1)
Red meat intake: *n* (%)				
Less than twice a week	88,953 (44.4)	121,203 (52.6)	37,834 (56.4)	83,369 (51.1)
Twice or more a week	109,271 (54.6)	107,142 (46.5)	28,812 (42.9)	78,330 (48.0)
Missing	2065 (1.0)	1981 (0.9)	460 (0.7)	1521 (0.9)

Abbreviations: HRT, hormone replacement therapy; IQR, interquartile range; *n* (%), number of participants (percentage from total in the cohort (for cohort size and cancer cases in men and women), or from total in women (for cohort size and cancer cases in pre‐ and post‐menopausal women), or from total per column for categorical variables); SD, standard deviation; WHO, World Health Organization; The definition of variables is described in Supplementary Methods. Tertile cut‐off points for the Townsend deprivation index were −3.23 and −0.80 for men, and −3.22 and −0.88 for women. Table S1 summarizes sun‐exposure‐related characteristics, which were used as covariates in models for skin squamous cell carcinoma and melanoma, and female‐specific characteristics, which were used as covariates in models for women.

### 3.2 Associations with obesity‐related cancers

**FIGURE 1:**
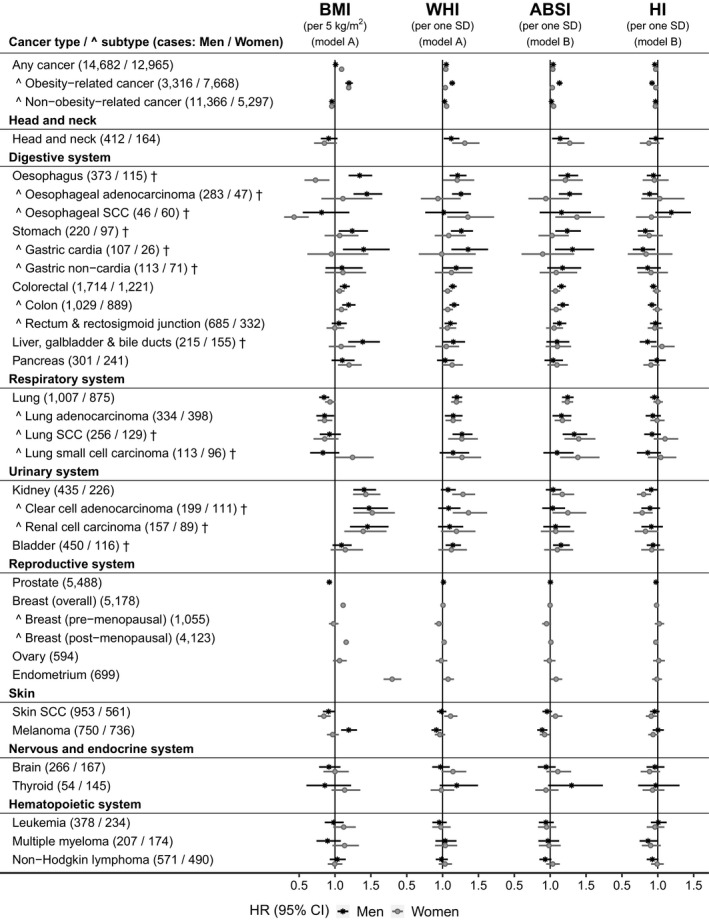
Allometric body shape indices in relation to cancer risk by sex. †—cancers with less than 20 cases in women pre‐menopausal at baseline, for which models were not adjusted for menopausal status; ABSI, a body shape index; BMI, body mass index; CI, confidence interval; HI, hip index; HR, hazard ratio; SCC, squamous cell carcinoma; SD, standard deviation; WHI, waist‐to‐hip index. HRs (95% CI) were obtained from delayed entry Cox proportional hazards models stratified by age at baseline and region of the assessment center. Model A—included BMI and WHI with adjustment variables. Model B—included BMI, ABSI, and HI with adjustment variables (HR estimates for BMI in Model A and Model B were similar). Men—models were adjusted for height, weight change during the year preceding baseline, Townsend deprivation index, smoking status, alcohol consumption, physical activity, consumption of fresh fruit and vegetables, processed meat and red meat, family history of cancer and, for skin SCC and melanoma, sun‐exposure‐related factors. Women—models included adjustment variables as for men, with the addition of menopausal status (except for cancers marked with †), use of hormone replacement therapy, ever use of oral contraceptives, and age at last live birth (with “no live births” as one of the categories). Cancer types and subtypes are defined in Supplementary Methods according to the 10th edition of the International Statistical Classification of Diseases (ICD10). Obesity‐related cancers included esophageal adenocarcinoma, cancers of the gastric cardia, colon, rectum and rectosigmoid junction, liver, gallbladder and bile ducts, pancreas, kidney, postmenopausal breast, ovary, endometrium, thyroid, and multiple myeloma. Non‐obesity‐related cancers included the remaining cancers. Numerical values for HR (95% CI) estimates are shown in Table S2

Obesity‐related cancers overall were associated positively with BMI, similarly in men (HR = 1.20, 95% CI = 1.14–1.25, per 5 kg/m^2^ increment) and women (HR = 1.19, 95% CI = 1.16–1.22), and also positively with ABSI, more prominently in men (HR = 1.13, 95% CI = 1.09–1.17, per one SD increment) compared to women (HR = 1.03, 95% CI = 1.01–1.05), but inversely with HI, again more prominently in men (HR = 0.92, 95% CI = 0.89–0.95, per one SD increment) compared to women (HR = 0.97, 95% CI = 0.95–1.00) (Figure 1).

**FIGURE 2:**
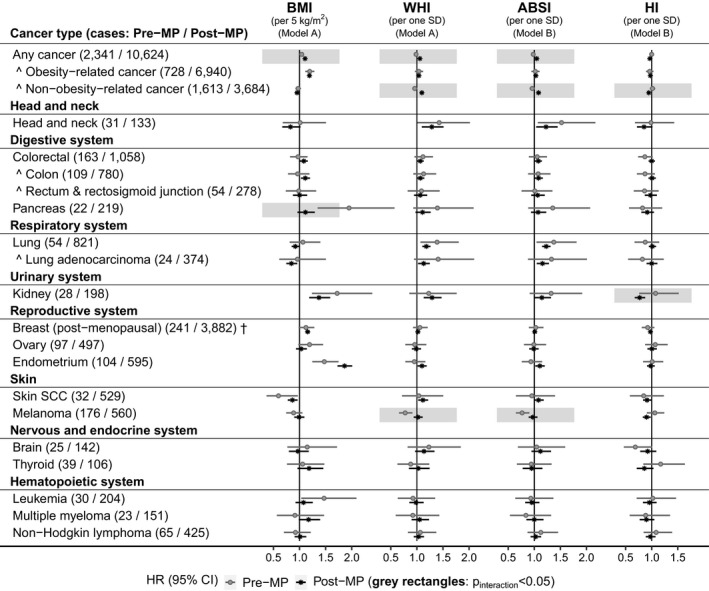
Allometric body shape indices in relation to cancer risk by menopausal status. ABSI, a body shape index; BMI, body mass index; CI, confidence interval; HI, hip index; HR, hazard ratio; SCC, squamous cell carcinoma; SD, standard deviation; WHI, waist‐to‐hip index. HRs (95% CI) were obtained from delayed entry Cox proportional hazards models stratified by age at baseline and region of the assessment center, for cancers with at least 20 cases in pre‐menopausal women. Model A—included BMI and WHI with adjustment variables. Model B—included BMI, ABSI, and HI with adjustment variables (HR estimates for BMI in Model A and Model B were similar). All models were adjusted for height, weight change during the year preceding baseline, Townsend deprivation index, smoking status, alcohol consumption, physical activity, consumption of fresh fruit and vegetables, processed meat and red meat, family history of cancer, use of hormone replacement therapy, ever use of oral contraceptives, age at last live birth (with “no live births” as one of the categories) and, for skin SCC and melanoma, sun‐exposure‐related factors. Pre‐MP—models in the subgroup of women pre‐menopausal at baseline; Post‐MP—models in the subgroup of women post‐menopausal at baseline; post‐menopausal—breast cancers diagnosed at age 55 years or older, irrespective of menopausal status at baseline; grey background rectangles—highlight interactions with menopausal status with nominal statistical significance (*p*_interaction_ < 0.05), corresponding to a Wald test for the interaction term between menopausal status at baseline and each obesity index, included in adjusted models for women (Model A for BMI or WHI and Model B for ABSI or HI, one interaction at a time). Cancer types and subtypes (marked with ^) are defined in Supplementary Methods according to the 10th edition of the International Statistical Classification of Diseases (ICD10). Obesity‐related cancers included esophageal adenocarcinoma, cancers of the gastric cardia, colon, rectum and rectosigmoid junction, liver, gallbladder and bile ducts, pancreas, kidney, postmenopausal breast, ovary, endometrium, thyroid, and multiple myeloma. Non‐obesity‐related cancers included the remaining cancers. Numerical values for HR (95% CI) estimates are shown in Table S2

Body mass index was associated positively with esophageal adenocarcinoma (HR = 1.44, 95% CI = 1.26–1.66) and cancers of the gastric cardia (HR = 1.40, 95% CI = 1.11–1.76), colon (HR = 1.19, 95% CI = 1.10–1.29), liver (HR = 1.39, 95% CI = 1.18–1.63), and kidney in men (HR = 1.41, 95% CI = 1.26–1.57, similarly for renal‐cell carcinoma [RCC] and clear‐cell adenocarcinoma) and, in women, with cancers of the colon (HR = 1.09, 95% CI = 1.01–1.17), kidney (HR = 1.43, 95% CI = 1.25–1.63), post‐menopausal breast (HR = 1.16, 95% CI = 1.12–1.19) and endometrium (HR = 1.80, 95% CI = 1.68–1.92) and, in pre‐menopausal women, also pancreas (HR = 1.95, 95% CI = 1.35–2.82, *p*_interaction_ = 0.013) (Figures 1 and 2).

In men, ABSI was associated positively with esophageal adenocarcinoma (HR = 1.27, 95% CI = 1.12–1.44) and cancers of the gastric cardia (HR = 1.31, 95% CI = 1.07–1.61), colon (HR = 1.18, 95% CI = 1.10–1.26), and rectum (HR = 1.13, 95% CI = 1.04–1.22), while HI was associated inversely with esophageal adenocarcinoma (HR = 0.89, 95% CI = 0.79–1.00) and cancers of the gastric cardia (HR = 0.79, 95% CI = 0.65–0.96), colon (HR = 0.92, 95% CI = 0.86–0.98), and liver (HR = 0.86, 95% CI = 0.75–0.98). In women overall, ABSI was associated positively with colon cancer (HR = 1.08, 95% CI = 1.01–1.16) and kidney clear‐cell carcinoma (HR = 1.25, 95% CI = 1.03–1.50), but not RCC. In post‐menopausal women, ABSI was also associated positively with endometrial cancer (HR = 1.11, 95% CI = 1.02–1.20, *p*_interaction_ = 0.084), but not breast cancer, while HI was associated inversely with kidney cancer (HR = 0.77, 95% CI = 0.67–0.88, *p*_interaction_ = 0.035).

Associations with WHI were directionally consistent and often similar in size to associations with ABSI.

### 3.3 Associations with non‐obesity‐related cancers

Although non‐obesity‐related cancers overall were associated inversely with BMI, similarly in men (HR = 0.96, 95% CI = 0.93–0.98) and women (HR = 0.95, 95% CI = 0.93–0.98), they were associated positively with ABSI in post‐menopausal women (HR = 1.08, 95% CI = 1.05–1.12, *p*_interaction_ < 0.001) and inversely with HI in men (HR = 0.97, 95% CI = 0.95–0.99) and post‐menopausal women (HR = 0.94, 95% CI = 0.91–0.98, *p*_interaction_ = 0.008) (Figures 1 and 2).

Notably, in women, ABSI was associated positively with SCC in the upper aerodigestive tract: head and neck (HR = 1.27, 95% CI = 1.10–1.48), esophagus (HR = 1.37, 95% CI = 1.07–1.76), and lung (HR = 1.40, 95% CI = 1.20–1.63), despite inverse associations of BMI with esophageal SCC (HR = 0.43, 95% CI = 0.29–0.63) and skin SCC (HR = 0.84, 95% CI = 0.76–0.94), while HI was associated inversely with skin SCC (HR = 0.91, 95% CI = 0.83–0.99). In men, ABSI was associated positively with head and neck (HR = 1.14, 95% CI = 1.03–1.26) and lung SCC (HR = 1.33, 95% CI = 1.17–1.52), while BMI was associated inversely with skin SCC (HR = 0.91, 95% CI = 0.83–0.99). Further for lung cancers, ABSI was associated positively with lung adenocarcinoma, similarly in men (HR = 1.16, 95% CI = 1.03–1.30) and women (HR = 1.17, 95% CI = 1.06–1.29), despite inverse associations with BMI, similar in men (HR = 0.85, 95% CI = 0.74–0.99) and women (HR = 0.85, 95% CI = 0.76–0.96), while small‐cell carcinoma was associated positively with both ABSI (HR = 1.39, 95% CI = 1.14–1.69) and BMI (HR = 1.24, 95% CI = 1.01–1.54), but only in women.

In addition in men, ABSI was associated positively with bladder cancer (HR = 1.15, 95% CI = 1.04–1.27), HI was associated inversely with multiple myeloma (HR = 0.86, 95% CI = 0.75–1.00), while only BMI (not body shape indices) was associated inversely with prostate cancer (HR = 0.92, 95% CI = 0.89–0.96).

Melanoma was unusually associated inversely with ABSI in men (HR = 0.89, 95% CI = 0.82–0.96) and pre‐menopausal women (HR = 0.77, 95% CI = 0.65–0.91, *p*_interaction_ = 0.006), despite being associated positively with BMI in men (HR = 1.19, 95% CI = 1.09–1.31) and inversely with HI in post‐menopausal women (HR = 0.90, 95% CI = 0.83–0.98).

Although we have highlighted associations with nominal statistical significance, some associations with borderline significance may merit further evaluation in larger datasets, for example, positive associations of ABSI with skin SCC in women, or with thyroid cancer in men (Figure 1).

### 3.4 Sensitivity analyses

**FIGURE 3:**
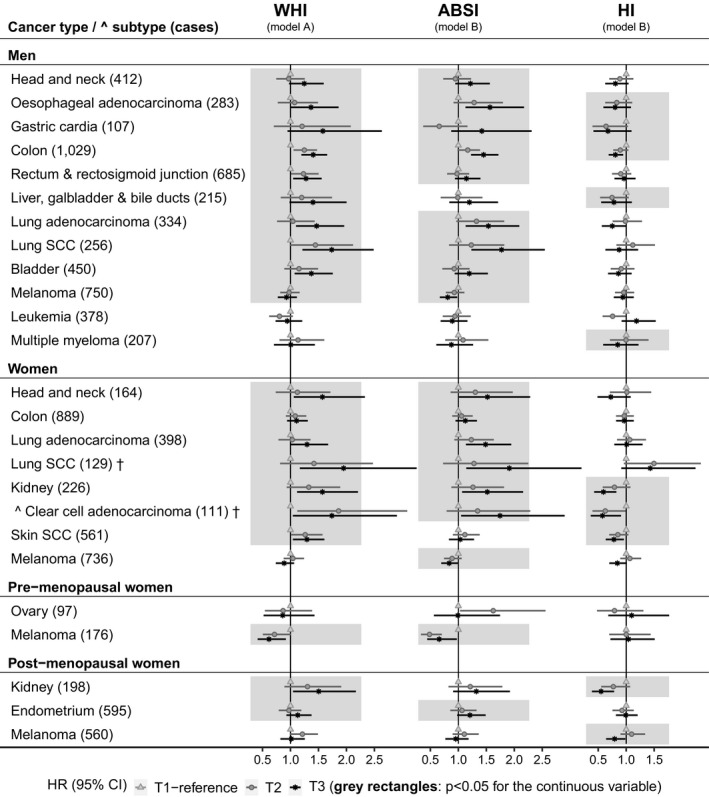
Allometric body shape index tertiles in relation to cancer risk by sex. †—cancers with less than 20 cases in women pre‐menopausal at baseline, for which models were not adjusted for menopausal status; ABSI, a body shape index; BMI, body mass index, categorized according to the World Health Organization as normal weight (≥18.5 to <25 kg/m^2^), overweight (≥25 to <30 kg/m^2^) or obese (≥30 to <45 kg/m^2^); CI, confidence interval; HI, hip index; HR, hazard ratio; SCC, squamous cell carcinoma; T1‐T3, sex‐specific tertiles for ABSI, HI, and WHI; WHI, waist‐to‐hip index. HRs (95% CI) were obtained from delayed entry Cox proportional hazards models stratified by age at baseline and region of the assessment center. Model A—included BMI and WHI with adjustment variables. Model B—included BMI, ABSI, and HI with adjustment variables. Men—models were adjusted for height, weight change during the year preceding baseline, Townsend deprivation index, smoking status, alcohol consumption, physical activity, consumption of fresh fruit and vegetables, processed meat and red meat, family history of cancer, and, for skin SCC and melanoma, sun‐exposure‐related factors. Women—models included adjustment variables as for men, with the addition of menopausal status (except for cancers marked with †), use of hormone replacement therapy, ever use of oral contraceptives, and age at last live birth (with “no live births” as one of the categories). Grey background rectangles—highlight associations with nominal statistical significance (*p *< 0.05) for the corresponding continuous variable, representing a test for linear trend. Cancer types and subtypes are defined in Supplementary Methods according to the 10th edition of the International Statistical Classification of Diseases (ICD10). This figure includes cancers showing statistically significant associations with allometric body shape indices used as continuous variables (highlighted in grey rectangles), or isolated associations with allometric body shape indices used as categorical variables. Numerical values for all HR (95% CI) estimates, derived from models for cancers with at least 10 cases per BMI category and body shape index tertile, are shown in Table S3

The observed associations remained reasonably consistent when using body shape indices as categorical variables, with the exception of some potentially U‐shaped associations as follows. There was a lower risk of lung SCC in men with overweight compared to normal‐weight BMI (HR = 0.72, 95% CI = 0.52–0.98) but not with obese BMI, a lower risk of leukemia for men in the middle compared to the lowest tertile of HI (HR = 0.76, 95% CI = 0.58–0.99) but not in the highest tertile, and a higher risk of ovarian cancer for pre‐menopausal women in the middle compared to the lowest tertile of ABSI (HR = 1.62, 95% CI = 1.03–2.56) but not in the highest tertile (Figure 3, Table S3). Adjustment for covariates had little influence overall, but attenuated associations of ABSI with lung and head and neck cancers, accounted for by adjustment only for smoking status. Adjustment for smoking status alone, however, had little influence on associations of BMI with lung and bladder cancers in women, for which the attenuation achieved by the fully adjusted model was accounted for mainly by weight change in the last year, Townsend deprivation index, and alcohol consumption. Omitting female‐specific or sun‐exposure‐related factors had no material influence (see Figure S2 for alternative adjustments in men and Figure S3 in women). The associations remained consistent after removing participants with less than 2 years of follow‐up (Figure S4), with the following exceptions. Associations of ABSI with lung adenocarcinoma (men), melanoma (women), and endometrial cancer were attenuated, while in men gained nominal statistical significance a positive association of ABSI with liver cancer (HR = 1.18, 95% CI = 1.01–1.38), an inverse association of HI with kidney clear‐cell carcinoma (HR = 0.85, 95% CI = 0.73–1.00), and inverse associations of HI (HR = 0.89, 95% CI = 0.81–0.99) and ABSI (HR = 0.90, 95% CI = 0.81–0.99) with non‐Hodgkin lymphoma. ABSI and HI calculated with coefficients from NHANES and from UK Biobank were very strongly correlated (Figure S5) and associations based on them showed no material difference.

### 3.5 Comparison with traditional body shape indices

**FIGURE 4:**
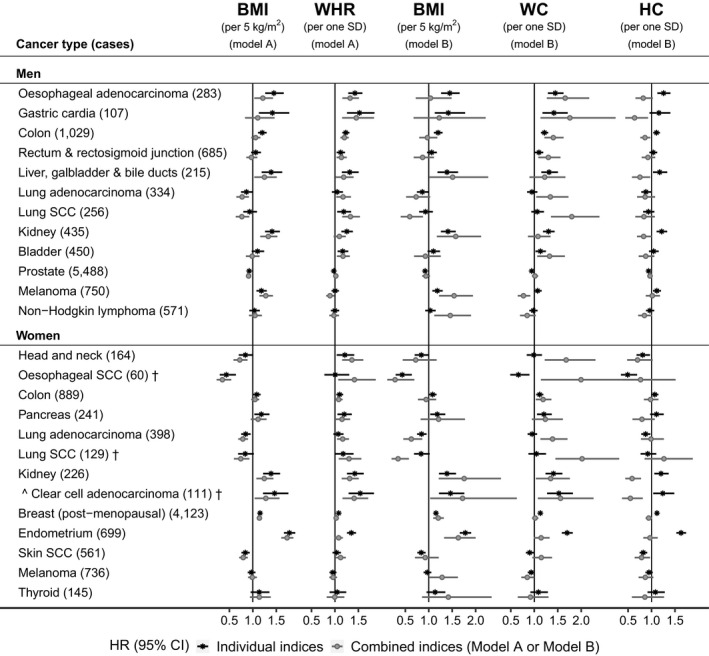
Traditional body shape indices in relation to cancer risk by sex. †—cancers with less than 20 cases in women pre‐menopausal at baseline, for which models were not adjusted for menopausal status; BMI, body mass index; CI, confidence interval; HC, hip circumference; HR, hazard ratio; SCC, squamous cell carcinoma; SD, standard deviation; WC, waist circumference; WHR, waist‐to‐hip ratio. HRs (95% CI) were obtained from delayed entry Cox proportional hazards models stratified by age at baseline and region of the assessment center. Individual indices—models included individually each of BMI, WHR, WC or HC with adjustment variables; Combined indices—models included combinations of obesity indices with adjustment variables; Model A—included BMI and WHR with adjustment variables. Model B—included BMI, WC, and HC with adjustment variables; Men—models were adjusted for height, weight change during the year preceding baseline, Townsend deprivation index, smoking status, alcohol consumption, physical activity, consumption of fresh fruit and vegetables, processed meat and red meat, family history of cancer and, for skin SCC and melanoma, sun‐exposure‐related factors. Women—models included adjustment variables as for men, with the addition of menopausal status (except for cancers marked with †), use of hormone replacement therapy, ever use of oral contraceptives, and age at last live birth (with “no live births” as one of the categories). Cancer types and subtypes are defined in Supplementary Methods according to the 10th edition of the International Statistical Classification of Diseases (ICD10). This figure includes individual cancers showing statistically significant associations with BMI or with allometric body shape indices used as continuous variables. For esophageal SCC in women, the upper limit of the 95% CI for WC in the models with combined indices was 3.49 and the lower limit for HC was 0.39. Numerical values for HR (95% CI) estimates are shown in Table S4

WC and HC were strongly positively correlated with BMI (Figure S5). When examined individually, they were strongly positively associated with obesity‐related cancers, similarly to BMI. When examined jointly with BMI, they showed HR estimates directionally consistent with estimates based on ABSI and HI, but with considerably larger confidence intervals and biased upwards for positive associations and downwards for inverse associations. Positive associations with BMI were introduced for some cancers (melanoma in women, non‐Hodgkin lymphoma in men), which were absent when BMI was examined individually (Figure 4). Although WHR was only moderately correlated with BMI (Figure S5) and showed associations comparable to WHI, HR estimates based on WHR for cancers strongly positively associated with BMI (kidney, endometrium) were biased upwards when WHR was not adjusted for BMI, while HR estimates based on BMI for cancers strongly positively associated with waist size (esophageal adenocarcinoma, gastric cardia, and colon cancers in men) were biased downwards when BMI was examined jointly with WHR (Figure 4).

## 4 DISCUSSION

In the first systematic study of associations between allometric body shape indices and the risk of cancer development in all major anatomical sites and morphologies, we report positive associations with waist size or inverse with hip size, independent of each other and of associations with BMI, extending beyond obesity‐related cancers. Unusually, waist size was associated inversely with melanoma in men and pre‐menopausal women.

The major differences between our findings and previous reports concern associations between body shape indices and cancers associated strongly with BMI.^3^ While published associations of WC and HC unadjusted for BMI resemble associations with BMI, that is, strongly positive for obesity‐related cancers of the endometrium,^18^ post‐menopausal breast^19^ or pancreas^20^ but inverse for esophageal SCC in women^21^ or prostate cancer in men,^22^ we did not find corresponding associations with ABSI or HI, independent of BMI. As regional body dimensions are strongly correlated with overall body size, risk estimates based on traditional body shape indices examined individually are influenced by associations with BMI. When WC and HC are combined with BMI, all three indices provide overlapping quantitative information for total body size, which reduces the specificity of WC and HC as measures of regional size and creates collinearity problems in classical regression models, resulting in biased risk estimates.^4^

The main compatibilities between our findings and previous reports concern cancers associated more strongly with waist size. Thus, positive associations based on WC or WHR, with or without adjustment for BMI and in some studies for HC, have previously been reported for cancers of the head and neck,^23^, ^24^, ^25^ esophagus and gastric cardia (adenocarcinoma),^21^, ^24^, ^26^ colon,^24^, ^27^, ^28^ liver,^24^, ^29^ lung overall,^30^ and bladder (men).^24^, ^31^, ^32^ Nevertheless, while we observed comparable associations with ABSI and WHI, previous studies have often reported positive associations with WC but not with WHR adjusted for BMI.^19^, ^33^ Some discrepancies for hip size may also be due to the mutual adjustment used in previous studies for HC and WC, which are correlated strongly with each other and, as explained above, are both proxy measures of total body size. Thus, we could not confirm a reported inverse association of HC adjusted for WC with head and neck cancer in men,^25^ although our results were directionally consistent in women, but found an inverse association of HI with liver cancer, not reported for HC adjusted for WC.^29^ Further, we could not confirm a reported inverse association of HC adjusted for body weight with RCC in men,^34^ but found evidence for an inverse association of HI with clear‐cell adenocarcinoma, especially in women.

Biological mechanisms linking body shape and cancer would likely involve insulin resistance, metabolic factors, and chronic inflammation, which are associated with visceral adiposity and smaller gluteofemoral size^2^, ^35^, ^36^; estrogens, which facilitate gluteofemoral fat accumulation; androgens, which direct fat centralization, as well as glucocorticoids, which regulate central obesity and metabolism^38^ and promote macrophage migration and chronic inflammation.^39^ The relationships, however, are likely to be complex. For example, liver steatosis, a feature of visceral adiposity, is driven in women by post‐menopausal estrogen reduction, which results in increased follicle‐stimulating hormone and subsequent hypersensitization of the glucocorticoid receptor.^40^ Further, glucocorticoids have pro‐ and anti‐inflammatory functions^41^ and act as immunomodulators regulating other signaling factors.^42^ Thus, altered glucocorticoid‐mediated signaling associated with abdominal obesity could facilitate cancer development independent of metabolic factors.^43^, ^44^, ^45^ Furthermore, cancer tissues modulate local cortisol activation‐deactivation enzymes toward increased local cortisol production, thus inhibiting tumor‐specific cytotoxic T‐cells and facilitating tumor growth.^46^ In addition, major genes associated with body shape code signaling factors involved in cell differentiation, proliferation, apoptosis, migration, and angiogenesis,^47^, ^48^ which may promote region‐specific cancer growth and have been associated with various cancers.^49^, ^50^ Indirect links are also possible. For example, inactivation of melanocortin receptors in mouse knockout models or in humans with genetic polymorphisms has been associated with abdominal obesity and metabolic syndrome,^51^ but could also be associated with lower risk of melanoma, which is linked to the activation of melanocortin receptors.^52^ A major question, however, remains, why major sex‐steroid‐related cancers (postmenopausal breast, prostate) are not associated overall with allometric body shape indices, when sex steroids are key determinants of body shape.^13^, ^37^

It is important, therefore, to obtain unbiased estimates of associations between body shape and specific cancers, independent of associations with BMI. Despite the caveat of not distinguishing lean from fat mass, BMI provides information for mass quantity and would reflect disturbances in the balance between energy supply and energy expenditure. When ABSI and HI are combined with BMI, they provide complementary information for mass distribution and potentially also for disturbances of the factors that determine body shape, offering additional insights into the mechanisms of cancer development, extending beyond energy balance and adipose‐derived factors. Thus, non‐obesity‐related cancers, which are associated only with body shape indices but not with BMI, would more likely be related to the factors determining the mass distribution and not to adipose‐derived factors or energy balance.

A strength of our study is the sizeable number of incident cancer cases, which permitted examining men and women separately and provided statistical power for the major cancer sites, although cancer cases and statistical power were limited for less‐common sites and morphologies. Anthropometric measurements were obtained by trained personnel, which avoided bias from self‐reported values. Models were adjusted for major lifestyle, dietary, and reproductive factors, thus minimizing confounding. Nevertheless, our study has several limitations. We could not examine ethnic variations, as UK Biobank includes participants with mainly white ethnic background. Ethnic minorities included in UK Biobank not only represent a small proportion of the cohort (<6%) but are also a heterogeneous group and could not provide adequate statistical power to examine associations with cancer risk. Mixing all ethnicities in a single analysis would not be appropriate as there are ethnic differences in body composition^53^, ^54^ and in the associations of body shape indices with more extensively studied outcomes such as mortality.^5^, ^55^ The genetic background of body shape would also differ between ethnicities, as the lead genetic variant for allometric body shape indices identified in UK Biobank was specific to European populations.^47^ It would, therefore, be important to explore ethnic differences in the association of body shape with cancer, but in datasets with appropriate size. Another limitation of our study was the assessment of body shape measurements at a single timepoint, at baseline. Longitudinal data would have allowed for a more accurate analysis but <10% of participants had two or three follow‐up measurements. We were also unable to examine associations of regional fat depots and muscle mass with cancer risk, as imaging measurements of body composition were limited. Further, we could not explore heterogeneity by receptor status for breast cancer, or by stage and aggressiveness for prostate cancer, due to the lack of relevant data. Furthermore, UK Biobank is not representative of the UK population. Participants are more likely to adhere to healthier lifestyles and less likely to be obese or to develop cancer compared to the general UK population of the same age,^56^ which may have influenced body shape and the observed associations with cancer risk. In addition, UK Biobank is a middle‐aged cohort, with fewer pre‐menopausal women, which hindered the evaluation of heterogeneity by menopausal status and prevented comparisons between early and later life. Categorical variables also provided limited information for nonlinear associations with body shape. Notably, nonlinear associations with mortality have been described not only for BMI, but also for HI^6^ and our results have suggested some U‐shaped associations of body shape indices with cancer. Examining nonlinear associations with continuous variables, however, would benefit from more statistical power and hence would be more adequately addressed in UK Biobank when further cancer cases are ascertained. In general, lack of statistical power may have resulted in chance findings for cancers with fewer cases. Nevertheless, even if the examined sample was not representative of the underlying population overall, it may still be informative for heterogeneities in the underlying biological mechanisms and be applicable to a specific cancer morphology or to part of the population with specific characteristics. Potential heterogeneities, however, would need to be explored and validated in cohorts with more cases. Finally, excluding participants with shorter follow‐up to evaluate potential reverse causality reduces statistical power and could introduce selection bias.

In conclusion, allometric body shape indices provide unbiased risk estimates for associations independent of BMI. Preferential positive associations of waist size or inverse of hip size with several major cancers indicate an important role of factors related to body shape in cancer development, potentially extending beyond the effects of regional fat depots.

## CONFLICT OF INTEREST

The authors declare no competing interests.

## AUTHOR CONTRIBUTIONS

S.C., K.K.T., and E.R. conceived and designed the study. K.K.T. and E.E. provided statistical advice. S.C. led the research and performed the statistical analysis. S.C. had full access to all of the data in this study and takes responsibility for the integrity of the data and the accuracy of the data analysis. S.C. drafted the paper with contributions from E.E, K.K.T, and E.R. All authors, that is, S.C., E.E., E.R., and K.K.T. were involved in the interpretation of the results, the critical revisions of the paper, and the approval of the final version of the manuscript.

## ETHICAL APPROVAL AND CONSENT TO PARTICIPATE

The UK Biobank cohort has been approved by the North West Multicenter Research Ethics Committee, UK (Ref: 16/NW/0274). Written informed consent has been obtained from all study participants. The current study was approved by the UK Biobank access management board. This research was conducted using the UK Biobank Resource (https://www.ukbiobank.ac.uk/about‐biobank‐uk/) under Application number 41952, granting access to the corresponding data.

## Supporting information

Supplementary Material

## Data Availability

The data supporting the findings of the study are available *to bona* *fide* researchers upon approval of an application to the UK Biobank (https://www.ukbiobank.ac.uk/researchers/) and a material transfer agreement.
