# Supplementary material for: A Body Shape Index (ABSI), hip index, and risk of cancer in the UK Biobank cohort
Source: Cancer Med. 2021 Jul 1;10(16):5614–28. doi: 10.1002/cam4.4097 (PMC8366087; doi:10.1002/cam4.4097)
Supplement: Supplementary file 1 — Supplementary Material [file CAM4-10-5614-s001.pdf]

# A Body Shape Index (ABSI), hip index and risk of cancer in the UK Biobank cohort

**Supplementary Methods**


Exclusion criteria ........ 2

Definition of prevalent and incident cancer ........ 2

Definition of cancer types and subtypes ........ 3

Definition of variables ........ 5

Calibration of allometric body shape indices for UK Biobank participants ........ 10


**Supplementary Tables**


Supplementary Table S1 Sun-exposure related characteristics of study participants ........ 12

Supplementary Table S2 Allometric obesity indices in relation to cancer risk ........ 13

Supplementary Table S3 Allometric obesity index categories in relation to cancer risk . 17

Supplementary Table S4 Traditional obesity indices in relation to cancer risk ........ 23


**Supplementary Figures**


Supplementary Figure S1 Flow diagram of UK Biobank participants included in the study ........ 26

Supplementary Figure S2 Sensitivity analyses with alternative adjustments in men ........ 27

Supplementary Figure S3 Sensitivity analyses with alternative adjustments in women . 29

Supplementary Figure S4 Sensitivity analyses excluding participants with less than two years of follow-up ........ 31

Supplementary Figure S5 Correlation between anthropometric indices ........ 33

# Supplementary Methods

## Exclusion criteria

Data for 502,488 participants were available, after removing participants who had withdrawn consent. In total, 71,873 participants were excluded after applying sequentially the exclusion criteria listed below, such that each excluded individual was counted only once:

1. Ethnic background restricted to self-reported white (n=29,809): Field [21000-0.0] "*Ethnic background*"; included codes: 1 "*White*", 1001 "*British*", 1002 "*Irish*", 1003 "*Any other white background*".
2. Missing or extreme anthropometry (n=7,130): missing waist-to-hip index, calculated from Field [48-0.0] "*Waist circumference*", Field [49-0.0] "*Hip circumference*", Field [50-0.0] "*Standing height*" and Field [21002-0.0] "*Weight*" OR Height < 130 cm OR Waist circumference < 50 cm or > 160 cm OR body mass index BMI < 18.5 kg/m$^2$ OR BMI ≥ 45 kg/m$^2$.
3. Sex chromosome aneuploidy or mismatch between genetic and self-reported sex (n=782): Field [22019-0.0] "*Sex chromosome aneuploidy*" (code 1) OR a mismatch between Field [22001-0.0] "*Genetic sex*" and Field [34-0.0] "*Sex (self-reported)*".
4. Age at baseline < 40 or > 70 years (rounded to an integer) (n=11).
5. Women pregnant at baseline (n=105): Field [3140-0.0] Pregnant; Answer: 1 "Yes".
6. Prevalent cancer at baseline (n=34,036), see definition in the next section below.

The total number of participants included in the main study dataset was **430,615**.

For analyses involving endometrial cancer, 40,611 women were excluded from the main study dataset due to hysterectomy prior to baseline. The total number of women included in the endometrial cancer dataset was **189,715**.

For analyses involving ovarian cancer, 16,274 women were excluded from the main study dataset due to bilateral oophorectomy prior to baseline. The total number of women included in the ovarian cancer dataset was **214,052**.

## Definition of prevalent and incident cancer

Information for prevalent cancers was obtained from the cancer registry and from self-reported cancer. Information for incident cancers was obtained from the cancer registry.

For the cancer registry, information was included in Fields [40005-0.0/16] "*Date of cancer diagnosis*"; Fields [40006-0.0/16] "*Type of cancer: ICD10*" (10$^{th}$ version of the International Statistical Classification of Diseases, applicable to both prevalent and incident cancers), Fields [40013-0.0/14] "*Type of cancer: ICD9*" (9$^{th}$ version of ICD, relevant only to prevalent cancers), Fields [40011-0.0/16] "*Histology of cancer tumour*" and Fields [40012-0.0/16] "*Behaviour of cancer*

*tumour*". We converted the cancer registry fields to long format, such that each instance for each participant was included in a separate row. As cancer types coded according to ICD9 contained only instances 0-14, two additional instances with missing values were created, such that there were instances 0-16 for all fields. In total, there were **118,718** entries for **89,766** participants in the cancer registry records. We excluded **47,305** entries, as follows:

1. Entries with missing information for date or type of diagnosis (n=11).
2. Cancer type codes which did not belong to malignant cancers, i.e. did not belong to the list 140 to 209 for ICD9 or did not begin with "C" for ICD10 coding (n=22,813).
3. Non-melanoma skin cancers (defined with code 173 for ICD9 or C44 for ICD10), except skin cancers with squamous cell morphology (codes 8070, 8071, 8072 or 8083 for histology) (n=24,418).
4. Tumours with topological code corresponding to a malignant cancer (140 to 209 for ICD9 or beginning with "C" for ICD10) but with behavioural codes 0 "*Benign*", 1 "*Uncertain whether benign or malignant*" or 2 "*Carcinoma in situ*", as these are similar, correspondingly, to tumours with codes 210-229, 235-239 and 230-234 for ICD9 or tumours with codes D10-D36, D37-D48 and D00-D09 for ICD10 (n=63).

In total, remained **71,413** malignant cancer entries for **58,548** participants.

Prevalent cancer was considered present, if there was entry in the cancer registry up to and including the date of cohort entry OR a self-reported prevalent cancer at baseline. Information on self-reported prevalent cancers was obtained from Fields [20001-0.0/5] "*Cancer code, self-reported*" (excluding codes: 1003 "*skin cancer*", 1060 "*non-melanoma skin cancer*", 1061 "*basal cell carcinoma*", 1073"*rodent ulcer*", 1072 "*cin/pre-cancer cells cervix*").

Incident cancer was considered present if there was entry in the cancer registry with a date of diagnosis after the date of attending an assessment centre at baseline. Cancer type and date of diagnosis corresponded to the first incident cancer. Cancers with behavioural codes 3 "*Malignant, primary site*" or 5 "*Malignant, microinvasive*" were considered cases. Participants with first incident cancer with behavioural code 6 "*Malignant, metastatic site*", 9 "*Malignant, uncertain whether primary or metastatic site*" or missing were censored at the date of cancer diagnosis.

In the total dataset (n=502,488), there were **36,036** prevalent cancers at baseline and **29,585** first primary incident cancers with behavioural code 3, **54** with behavioural code 5, **300** with behavioural codes 6 or 9 and **11** with a missing behavioural code.

**Definition of cancer types and subtypes**

Topological and morphological (histological) codes for incident cancers were defined according to the 10th version of the International Statistical Classification of Diseases (ICD10).

Obesity-related cancers included oesophageal adenocarcinoma, cancers of the gastric cardia, colon, rectum and rectosigmoid junction, liver, gallbladder and bile ducts, pancreas, kidney, postmenopausal breast, ovary, endometrium, thyroid and multiple myeloma. Meningioma was not considered as cases were limited.

Non-obesity-related cancers included cancers not specified for the obesity-related group.

Cancer types and subtypes (^) below are ordered by body system.

1. Head and neck: ICD10 codes: C01, C02, C03, C04, C05, C06, C09, C10, C11, C12, C13, C14, C30, C31 and C32; Excluded rate morphologies: 8800, 8850, 9120, 9220, 9591, 9673, 9680, 9690, 9691, 9734.
2. Oesophagus (overall): ICD10 code C15; Excluded rare morphologies: 8170, 8720, 9591.
3. ^ Oesophageal adenocarcinoma: ICD10 code C15; Morphologies: 8140, 8144.
4. ^ Oesophageal squamous cell carcinoma: ICD10 code C15; Morphologies: 8070, 8071, 8072.
5. Stomach (overall): ICD10 code C16; Excluded rare morphologies: 8560, 8800, 8891, 8936, 9590, 9591, 9595, 9663, 9670, 9676, 9680, 9699, 9702, 9711, 9715, 9823.
6. Gastric cardia: ICD10 code C16.0; Excluded rare morphologies as for stomach (overall).
7. Gastric non-cardia: any remaining stomach cancer not included in category gastric cardia.
8. Colorectal (overall): ICD10 codes C18, C19, C20; Excluded rare morphologies: 8240, 8241, 8243, 8245, 8246, 8472, 8743, 8936, 9680, 9699.
9. ^ Colon: ICD code C18; Excluded rare morphologies: as for colorectal (overall).
10. ^ Rectum and rectosigmoid junction: ICD codes C19 and C20; Excluded rare morphologies: as for colorectal (overall).
11. Liver (overall) (including gallbladder and bile ducts): ICD codes C22.0, C22.1, C23, C24; Excluded rare morphologies: 9120, 9130, 9590, 9680.
12. Pancreas: ICD code C25; Excluded rare morphologies: 8150, 8151, 8152, 8246, 9591.
13. Lung (overall): ICD10 code C34; Excluded rare morphologies: 8710, 8800, 8801, 8990, 9050, 9120, 9133, 9591, 9680, 9699.
14. ^ Lung adenocarcinoma: ICD10 code C34; Morphologies: 8140, 8144, 8230, 8250, 8253, 8254, 8256, 8257, 8260, 8265, 8333, 8480, 8551.
15. ^ Lung squamous cell carcinoma: ICD10 code C34; Morphologies: 8070, 8071, 8072, 8083.
16. ^ Lung small cell carcinoma: ICD10 code C34; Morphologies: 8041, 8045.
17. Kidney (overall): ICD10 code C64; Excluded rare morphologies: 8800, 8830, 8890, 8964.
18. ^ Clear cell adenocarcinoma: ICD10 code C64; Morphology: 8310.
19. ^ Renal cell carcinoma: ICD10 code C64; Morphology: 8312.
20. Bladder: ICD10 code C67; Excluded rare morphologies: 8800, 8801, 8980, 9590, 9671.
21. Prostate: ICD10 code C61 in men.
22. Breast (overall): ICD10 code C50 in women; Excluded rare morphologies: 8801, 8804, 8810, 8980, 8982, 9020, 9120, 9590, 9675, 9690, 9691.

23. ^ Breast (pre-menopausal): as breast (overall) but diagnosed before age 55 years in women pre-menopausal at baseline.
24. ^ Breast (post-menopausal): as breast (overall) but diagnosed at age 55 years or later, irrespective of the menopausal status at baseline, censoring women with breast cancer diagnosed before age 55 years.
25. Ovary: ICD10 codes C56, C48, C57.0 in women; Excluded rare morphologies: 8890, 8980, 9680.
26. Endometrium: ICD10 code C54.1 in women; Excluded rare morphologies: 8890, 8930, 8931, 8933, 8935, 8950, 8951, 8980, 9100.
27. Skin squamous cell carcinoma: ICD10 code C44; Morphologies: 8070, 8071.
28. Melanoma: ICD10 code C43; Morphologies: 8720 – 8780.
29. Brain: ICD10 code C71; Excluded rare morphologies: 8420, 8510, 9120, 9150, 9473, 9490, 9590, 9591, 9680, 9702, 9732.
30. Thyroid: ICD10 code C73; Excluded rare morphologies: 8012, 8021, 8041, 8345, 8510, 8511, 9680.
31. Leukaemia: ICD10 codes C91, C92, C93, C94, C95.
32. Multiple myeloma: ICD10 code C90; Morphologies: 9731, 9732.
33. Non-Hodgkin lymphoma: ICD10 codes C82, C83, C84, C85.

The above definitions are based on [11], in which cancer types and subtypes were defined according to the 2nd version of the International Classification of Diseases for Oncology (ICD-O-2).

**Definition of variables**

Missing values for covariates were replaced with the sex-specific median category, if missingness was less than 5% for both sexes. A separate category “Missing” was created when missingness was larger than 5%. The proportion of missing values is shown in Table 1.

Date of birth was constructed from Field [34-0.0] “*Year of birth*” and Field [52-0.0] “*Month of birth*”, using 15 as the day of birth for all participants.

Age at baseline was calculated in years as the difference between Field [53-0.0] “*Date of attending assessment centre*” and the date of birth (as defined above), divided by 365.25. Six five-year categories were used for stratification (40 to <45 years, 45 to <50 years, 50 to <55, 55 to <60 years, 60 to <65 years and 65 to 70 years).

Region of the assessment centre at baseline was based on Field [54-0.0] “*UK Biobank assessment centre*”. Assessment centres were grouped in 10 geographic regions as follows: London, including Barts (centre ID number 11012), Hounslow (11018) and Croydon (11020); North-West, including Bury (11008), Liverpool (11016), Manchester (11001) and Stockport (pilot, 10003); North-East, including Middlesbrough (11017) and Newcastle (11009); Yorkshire and

Humber, including Leeds (11010) and Sheffield (11014); West Midlands, including Birmingham (11021) and Stoke (11006); East Midlands, including Nottingham (11013); South-East, including Oxford (11002) and Reading (11007); South-West, including Bristol (11011); Wales, including Cardiff (11003), Swansea (11022) and Wrexham (11023); Scotland, including Edinburgh (11005) and Glasgow (11004).

Weight change during last year preceding baseline was self-reported in Field [2306-0.0] "*Weight change compared with 1 year ago*"; Question: "*Compared with one year ago, has your weight changed?*" with three valid answers: 0 *"No - weigh about the same",* 2 *"Yes - gained weight",* 3 *"Yes - lost weight".* Missing values were replaced with category "No weight change" for both sexes.

Smoking status was defined as follows: Current smoker – was based on Field [1239-0.0] "*Current tobacco smoking*"; Question: *"Do you smoke tobacco now?"*; Answer 1: "*Yes, on most or all days*" or 2: "*Only occasionally*"; Former regular smoker – was based on Field [1249-0.0] "*Past tobacco smoking*"; Question: "*In the past, how often have you smoked tobacco?*"; Answer 1: "*Smoked on most or all days*", when the answer to Filed [1239-0.0] was not 1 or 2; Former occasional smoker – was based on Field [1249-0.0] Answer 2: "*Smoked occasionally*" or 3: "*Just tried once or twice*" when the answer to Filed [1239-0.0] was not 1 or 2; Never smoked – was based on Field [1249-0.0] Answer 4: "*I have never smoked*" when the answer to [Filed 1239-0.0] was not 1 or 2. Missing values were replaced with category "Former occasional smoker" for both sexes.

Alcohol consumption was based on Field [1558-0.0] "*Alcohol intake frequency*"; Question: "*About how often do you drink alcohol?*" as follows: Up to 3 times a month – Answer 4: "*One to three times a month*"; or 5: "*Special occasions only*"; or 6: "*Never*"; Up to four times a week – Answer 2: "*Three or four times a week*"; or 3: "*Once or twice a week*"; Daily or almost daily – Answer 1: "*Daily or almost daily*". Missing values were replaced with category "Up to four times a week" for both sexes.

Physical activity was defined as follows: Very active – was based on Field [816-0.0] "*Job involves heavy manual or physical work*"; Question: "*Does your work involve heavy manual or physical work?*"; Answer 3: "*Usually*" or 4: "*Always*"; OR Field [904-0.0] "*Number of days/week of vigorous physical activity 10+ minutes*"; Question: "*In a typical WEEK, how many days did you do 10 minutes or more of vigorous physical activity? (These are activities that make you sweat or breathe hard such as fast cycling, aerobics, heavy lifting)*"; Answer (numerical) 3-7; Moderately active – was based Field [904-0.0] Answer 1-2 OR Field [884-0.0] "*Number of days/week of moderate physical activity 10+ minutes*"; Question: "*In a typical WEEK, on how many days did you do 10 minutes or more of moderate physical activities like carrying light loads, cycling at normal pace? (Do not include walking)*"; Answer (numerical) 3-7; OR Field [864-0.0] "*Number of days/week walked 10+ minutes*"; Question: "*In a typical WEEK, on how many days did you walk for*

*at least 10 minutes at a time? (Include walking that you do at work, travelling to and from work, and for sport or leisure)*"; Answer (numerical) 7, when participants were not already included in category very active; Less active - was based on Field [904-0.0] Answer (numerical) 0 OR Field [884-0.0] Answer (numerical) 0-2 OR Field [864-0.0] Answer (numerical) 0-6 or -2: "*Unable to walk*", when participants were not already included in category moderately or very active. Missing values were replaced with category "Moderately active" for both sexes.

Townsend deprivation index was used as an indicator of socioeconomic status and was based on Field [189-0.0] "*Townsend deprivation index at recruitment*" (continuous) calculated by UK Biobank. This variable represents a score corresponding to the output area in which the participant's postcode was located immediately prior to joining UK Biobank, based on the preceding national census output areas. A greater score implies a greater degree of material deprivation. Missing values were replaced with the middle tertile for both sexes.

Fresh fruit and vegetable intake was based on the sum of two fields: Field [1309-0.0] "*Fresh fruit intake*" (continuous), Question: "*About how many pieces of FRESH fruit would you eat per DAY? (Count one apple, one banana, 10 grapes etc as one piece; put '0' if you do not eat any)*" and Field [1299-0.0] "*Salad / raw vegetable intake*" (continuous), Question: "*On average how many heaped tablespoons of SALAD or RAW vegetables would you eat per DAY? (Include lettuce, tomato in sandwiches; put '0' if you do not eat any)*". Answers: -10 *"Less than one"* were re-coded to 0.5. Answers: -1 "*Do not know*" and -3 "*Prefer not to answer*" were consider missing. The total was dichotomised as Less than five portions a day or Five or more portions a day and were used as an indication of a healthy lifestyle. Missing values were replaced with category "Less than five portions a day" for both sexes.

Processed meat intake was based on Field [1349-0.0] "*Processed meat intake*", Question: *"How often do you eat processed meats (such as bacon, ham, sausages, meat pies, kebabs, burgers, chicken nuggets)"*. Category Less than twice a week included answers: 0 "Never", 1 "Less than once a week" and 2 "Once a week". Category Twice or more a week included answers: 3 "*2-4 times a week*", 4 "*5-6 times a week*" 5 "*Once or more daily*". Answers: -1 "*Do not know*" and -3 "*Prefer not to answer*" were consider missing. Missing values were replaced with category "Less than twice a week" for both sexes.

Red meat intake was based on the sum of three fields: Field [1369-0.0] "*Beef intake*", Question: "*How often do you eat beef? (Do not count processed meats)*", Field [1379-0.0] "*Lamb/mutton intake*", Question: "*How often do you eat lamb/mutton? (Do not count processed meats)*" and Field [1389-0.0] "*Pork intake*", Question: *"How often do you eat pork? (Do not count processed meats such as bacon or ham)"*. The categorical answers were converted to a continuous scale as follows: Answer 0 "Never" remained 0; Answer 1 "Less than once a week" was coded as 0.5; Answer 2 "Once a week" was coded as 1; Answer 3 "2-4 times a week" was coded as 3; Answer 4 "5-6 times a week" was coded as 5.5; Answer 5 "Once or more daily" was coded as

7. Answers: -1 "*Do not know*" and -3 "*Prefer not to answer*" were consider missing. Categories Less than twice a week and Twice or more a week were derived with respect to the total of the three variables. Missing values were replaced with category "Twice or more a week" for men or category "Less that twice a week" for women.

Family history of cancer was based on three variables: Fields [20107-0.0/9] "*Illness of father*", Question: "*Has/did your father ever suffer from? (You can select more than one answer)"*, Fields [20110-0.0/10] "*Illness of mother*", Question: *"Has/did your mother ever suffer from? (You can select more than one answer)"* and Field [20111-0.0/11] "*Illness of siblings*", Question: "*Have any of your brothers or sisters suffered from any of the following diseases? (You can select more than one answer)".* Category Yes was based on Answers: 3 "*Lung cancer*", 4 "*Bowel cancer*", 5 "*Breast cancer*" or 13 "*Prostate cancer*" to any of the three sets of fields and category No included the remaining participants.

Hormone replacement therapy (HRT) use was determined for women by Field [2814-0.0] "*Ever used hormone-replacement therapy (HRT)*"; Question: "*Have you ever used hormone replacement therapy (HRT)?*"; Answer 0: "*No*" (for Never user) or Answer 1: "Yes" and Field [3546-0.0] "*Age last used hormone-replacement therapy (HRT)*" Question: *"How old were you when you last used HRT?"* Answer -11: "*Still taking HRT"* (for Current user) or else Answer 1: "Yes" to Field [2814-0.0] (for Former user). Further information was derived from Fields [6153-0.0/3] "*Medication for cholesterol, blood pressure, diabetes, or take exogenous hormones*", Question: "*Do you regularly take any of the following medications? (You can select more than one answer)"*. Women providing Answer 4 "*Hormone replacement therapy*" were considered Current user. Missing values were replaced with category "Never user".

Use of oral contraceptives was determined for women by Field [2784-0.0] "*Ever taken oral contraceptive pill*"; Question: "*Have you ever taken the contraceptive pill? (include the 'mini-pill')*"; Answer 0: "*No*" (for Never user) or Answer 1: "Yes" (for Ever user). Further information was derived from Fields [6153-0.0/3]. Women providing Answer 5 "*Oral contraceptive pill or minipill*" were considered Ever user. Missing values were replaced with category "Ever user".

Age at last live birth was defined as follows: No live births - was based on Field [2734-0.0] "*Number of live births*"; Question: "*How many children have you given birth to? (Please include live births only)*"; Answer (numerical) 0; < 30 years or ≥ 30 years – was based on Field [2764-0.0] Age at last live birth; Question: "*How old were you when you had your LAST child?*" and Field [3872-0.0] "*Age of primiparous women at birth of child*"; Question: "*How old were you when you had your child?*" (UK Biobank note: "Current Field was collected from women who indicated they had given birth to only one child, as defined by their answers to Field 2734"). Missing values were replaced with category "< 30 years".

Menopausal status was determined as follows: Post-menopausal – were classified women with age at baseline ≥58 years OR with bilateral oophorectomy from Field: [2834-0.0] "*Bilateral oophorectomy (both ovaries removed)*"; Question: "*Have you had BOTH ovaries removed?*"; Answer 1: "*Yes*" OR Field [20004-0] "*Operation code (self-reported operation)*" code: 1355 "*bilateral oophorectomy*"; OR with self-reported post-menopausal status from Field [2724-0.0] "*Had menopause*"; Question: "*Have you had your menopause (periods stopped)?*"; Answer 1: "Yes" OR with age at baseline ≥ 55 years when menopausal status was unknown, i.e. they had not answered 0: "No" to Field [2724-0.0]; Pre-menopausal – were classified women who had not been defined as post-menopausal above and had reported pre-menopausal status with Answer 0: "No" to Field [2724-0.0] OR had age at baseline < 55 years when menopausal status was unknown, i.e. not defined as post- or pre-menopausal according to the above criteria.

Bilateral oophorectomy prior to baseline was determined as follows: women with Answer 1: "Yes" for Field: [2834-0.0] "*Bilateral oophorectomy (both ovaries removed)*" OR code 1355 for Field [20004-0] "*Operation code (self-reported operation)*".

Hysterectomy prior to baseline was determined as follows: women with Answer 1: "Yes" for Field: [3591-0.0] "*Ever had hysterectomy (womb removed)*"; Question: "*Have you had a hysterectomy (womb removed)*" OR codes 1357 "*hysterectomy*", 1358 "*hysterectomy with oophorectomy*", 1359 "*hysterectomy with cervical sparing*", for Field [20004-0] "*Operation code (self-reported operation)*".

Skin colour was based on Field [1717-0.0] "*Skin colour*"; Question: *"What best describes the colour of your skin without tanning?"*; Answer 1: "*Very fair*" (for Very fair); Answer 2: "*Fair*" (for Fair); Answer 3: "*Light olive*", or Answer 4: "*Dark olive*", or Answer 5: "*Brown*", or Answer 6: "*Black*" (for Dark). Missing values were replaced with category "Fair" for both sexes.Ease of skin tanning was based on Field [1727-0.0] "*Ease of skin tanning*"; Question: *"What would happen to your skin if it was repeatedly exposed to bright sunlight without any protection?"*; Answer 1: "Get very tanned"; Answer 2: "Get moderately tanned"; Answer 3: "Get mildly or occasionally tanned"; Answer 4: "Never tan, only burn". Missing values were replaced with category "Get moderately tanned" for both sexes.

Hair colour was based on Field [1747-0.0] *"Hair colour (natural, before greying)";* Question: *"What best describes your natural hair colour? (If your hair colour is grey, the colour before you went grey)"*; Answer 1: "*Blond*" or Answer 2: "*Red*" (for Blond or red); Answer 3: "*Light brown*" (for Light brown); Answer 4: "*Dark brown*", or Answer 5: "*Black*", or Answer 6: "*Other*" (for Dark). Missing values were replaced with category "Light brown" for both sexes.

Sunburn in childhood was based on Field [1737-0.0] *"Childhood sunburn occasions";* Question: *"Before the age of 15, how many times did you suffer sunburn that was painful for at least 2 days or caused blistering?"*; Answer numerical 0 (for Never burned); Answer any positive numerical (for Ever burned); missing, or Answer -1: "*Do not know*", or Answer -3: "*Prefer not to answer*" (for Missing).

Solarium use was based on Field [2277-0.0] *"Frequency of solarium/sunlamp use";* Question: *"How many times a year would you use a solarium or sunlamp?";* Answer -10: "*Less than once a year*" or any positive numerical value (for Ever use); not missing, and not Answer -1: "*Do not know*", and not Answer -3: "*Prefer not to answer*" (for Never use). Missing values were replaced with category "Never use" for both sexes.

Sun / UV protection was based on Field [2267-0.0] *"Use of sun/UV protection";* Question: *"Do you wear sun protection (e.g. sunscreen lotion, hat) when you spend time outdoors in the summer?";* Answer 1: "Never / rarely"; Answer 2: "Sometimes"; Answer 3: "Most of the time"; Answers 4 or 5: "Always / do not go out in sunshine". Missing values were replaced with category "Sometimes" for men or category "Most of the time" for women.

Time spent outdoors in summer was based on Field [1050-0.0] *"Time spent outdoors in summer";* Question: *"In a typical DAY in summer, how many hours do you spend outdoors?";* Answer -10: "*Less than an hour a day*" or numerical 1-3 (for ≤ 3 hours a day); Answer positive numerical >3 (for > 3 hours a day); missing, or Answer -1: "*Do not know*", or Answer -3: "*Prefer not to answer*" (for Missing).

**Calibration of allometric body shape indices for UK Biobank participants**

Allometric body-shape indices calibrated for UK Biobank participants were calculated according to the general formula:

$$Index = Measure * Weight^{-\beta} * Height^{-\gamma}$$

where $\beta$ and $\gamma$ are the scaling power coefficients for weight and height and *Measure* is either WC for A Body Shape Index ($ABSI_{UKB}$), HC for Hip Index ($HI_{UKB}$) or WHR for the Waist-to-Hip Index ($WHI_{UKB}$).

The scaling power coefficients were derived from log-linear models regressing each of log-transformed waist circumference (WC, Field [48-0.0]), hip circumference (HC, Field [49-0.0]) or the waist-to-hip ratio (WHR, the ratio of Field [48-0.0] to Field [49-0.0]) on log-transformed waist (Field [21002-0.0]) and height (Field [50-0.0]). The log-linear regression coefficients for each allometric index are shown below (in brackets are shown standard errors):

**Women**

log(WC, mm) ~ 4.09709 (0.00341) + 0.72201 (0.00082) * log(Weight, kg) - 0.90129 (0.00377) * log(Height, m)

log(HC, cm) ~ 4.36243 (0.01075) + 0.48661 (0.00048) * log(Weight, kg) - 0.35326 (0.00220) * log(Height, cm)

log(WHR) ~ 1.58264 (0.02058) + 0.23540 (0.00091) * log(Weight, kg) - 0.54803 (0.00421) * log(Height, cm)

The models for women explained 77.2% of the variability of log(WC), 82.2% of the variability of log(HC) and 23.2% of the variability of log(WHR).

**Men**

log(WC, mm) ~ 4.25766 (0.00329) + 0.69550 (0.00082) * log(Weight, kg) - 0.84693 (0.00331) * log(Height, m)

log(HC, cm) ~ 3.81061 (0.01062) + 0.39806 (0.00054) * log(Weight, kg) - 0.18233 (0.00221) * log(Height, cm)

log(WHR) ~ 2.04471 (0.01677) + 0.29744 (0.00086) * log(Weight, kg) - 0.66460 (0.00349) * log(Height, cm)

The models for men explained 78.7% of the variability of log(WC), 75.0% of the variability of log(HC) and 37.8% of the variability of log(WHR).

It should be noted, that allometric indices calibrated for a given dataset are proportional to the residuals of log-linear models regressing out associations with weight and height, i.e. adjusting for weight and height each body-shape measure. The only part of the log-linear model omitted from the allometric formula is the intercept, which is a constant. As body mass index (BMI) is a combination of weight and height, the same models can also be re-parameterised to adjust for BMI and height, instead of weight and height, as previously explained [4, 5].

To examine associations between ABSI and HI calibrated for participants in the National Health and Nutrition Examination Survey (NHANES) (5, 6) and $ABSI_{UKB}$ and $HI_{UKB}$ calibrated for UK Biobank participants, we used partial Pearson correlation coefficients, with adjustment for age at baseline and region of the assessment centre. We also repeated the main analyses with $ABSI_{UKB}$, $HI_{UKB}$ and $WHI_{UKB}$. Note that although both $WHI_{UKB}$ and WHI are calibrated for UK Biobank participants, $WHI_{UKB}$ uses the exact regression coefficients for the dataset in this study, while WHI uses coefficients rounded to simple fractions. The simplified version of WHI can be calculated with a conventional calculator with the following sequence, dependent on how square root is required to be entered on the specific device (prior to or after the number):

WC (cm) / HC (cm) / Weight (kg) √ √ * Height (cm) √ =

or

WC (cm) / HC (cm) / √ √ Weight (kg) * √ Height (cm) =

where **/** stands for key “division”, ***** stands for key “multiplication”, √ stands for key “square root” and **=** stands for key “equal to”. For example, if an individual has WC 105 cm, HC 95 cm, Weight 85 kg and Height 170 cm, WHI would be:

105 / 95 / √ √ 85 * √ 170 =

or

105 / 95 / 85 √ √ * 170 √ =

dependent on the function of the “square root” key in the calculator. The answer would be 4.746.

**Supplementary Table S1 Sun-exposure related characteristics of study participants**

| Characteristics | Men | Women | Pre-menopausal | Post-menopausal |
|---|---|---|---|---|
| Cohort size: n (%) | 200,289 (46.5) | 230,326 (53.5) | 67,106 (29.1) | 163,220 (70.9) |
| Cancer cases: n (%) | 14,682 (53.1) | 12,965 (46.9) | 2,341 (18.1) | 10,624 (81.9) |
| Skin colour: n (%) | | | | |
| Very fair | 12,872 (6.4) | 21,102 (9.2) | 7,081 (10.6) | 14,021 (8.6) |
| Fair | 143,369 (71.6) | 159,282 (69.2) | 45,132 (67.3) | 114,150 (69.9) |
| Dark | 40,990 (20.5) | 47,680 (20.7) | 14,471 (21.6) | 33,209 (20.3) |
| Missing | 3,058 (1.5) | 2,262 (1.0) | 422 (0.6) | 1,840 (1.1) |
| Ease of skin tanning: n (%) | | | | |
| Get very tanned | 51,530 (25.7) | 35,849 (15.6) | 10,541 (15.7) | 25,308 (15.5) |
| Get moderately tanned | 82,411 (41.1) | 87,305 (37.9) | 25,035 (37.3) | 62,270 (38.2) |
| Get mildly/occasionally tanned | 33,467 (16.7) | 55,907 (24.3) | 17,220 (25.7) | 38,687 (23.7) |
| Never tan/ only burn | 28,185 (14.1) | 44,577 (19.4) | 12,676 (18.9) | 31,901 (19.5) |
| Missing | 4,696 (2.3) | 6,688 (2.9) | 1,634 (2.4) | 5,054 (3.1) |
| Hair colour: n (%) | | | | |
| Blond or red | 26,792 (13.4) | 40,216 (17.5) | 12,036 (17.9) | 28,180 (17.3) |
| Light brown | 77,808 (38.8) | 95,963 (41.7) | 27,457 (40.9) | 68,506 (42.0) |
| Dark | 95,053 (47.5) | 93,975 (40.8) | 27,561 (41.1) | 66,414 (40.7) |
| Missing | 636 (0.3) | 172 (0.1) | 52 (0.1) | 120 (0.1) |
| Sunburn in childhood: n (%) | | | | |
| Never burned | 69,208 (34.6) | 97,786 (42.5) | 23,724 (35.4) | 74,062 (45.4) |
| Ever burned | 77,738 (38.8) | 76,642 (33.3) | 29,177 (43.5) | 47,465 (29.1) |
| Missing † | 53,343 (26.6) | 55,898 (24.3) | 14,205 (21.2) | 41,693 (25.5) |
| Solarium use | | | | |
| Never use | 181,813 (90.8) | 201,413 (87.4) | 55,214 (82.3) | 146,199 (89.6) |
| Ever use | 15,856 (7.9) | 25,585 (11.1) | 10,812 (16.1) | 14,773 (9.1) |
| Missing | 2,620 (1.3) | 3,328 (1.4) | 1,080 (1.6) | 2,248 (1.4) |
| Sun / UV protection: n (%) | | | | |
| Never / rarely | 26,753 (13.4) | 9,178 (4.0) | 2,242 (3.3) | 6,936 (4.2) |
| Sometimes | 80,824 (40.4) | 62,235 (27.0) | 17,005 (25.3) | 45,230 (27.7) |
| Most of the time | 63,352 (31.6) | 93,366 (40.5) | 29,322 (43.7) | 64,044 (39.2) |
| Always / do not go out in sunshine | 27,685 (13.8) | 63,756 (27.7) | 17,935 (26.7) | 45,821 (28.1) |
| Missing | 1,675 (0.8) | 1,791 (0.8) | 602 (0.9) | 1,189 (0.7) |
| Time spent outdoors in summer: n (%) | | | | |
| ≤ 3 hours a day | 90,344 (45.1) | 120,633 (52.4) | 41,246 (61.5) | 79,387 (48.6) |
| > 3 hours a day | 100,095 (50.0) | 93,284 (40.5) | 21,713 (32.4) | 71,571 (43.8) |
| Missing † | 9,850 (4.9) | 16,409 (7.1) | 4,147 (6.2) | 12,262 (7.5) |
| Hormone replacement therapy: n (%) ‡ | | | | |
| Never user | - | 140,492 (61.0) | 62,651 (93.4) | 77,841 (47.7) |
| Former user | - | 71,514 (31.0) | 1,890 (2.8) | 69,624 (42.7) |
| Current user | - | 17,766 (7.7) | 2,372 (3.5) | 15,394 (9.4) |
| Missing | | 554 (0.2) | 193 (0.3) | 361 (0.2) |
| Oral contraceptives: n (%) ‡ | - | | | |
| Never user | - | 40,251 (17.5) | 5,799 (8.6) | 34,452 (21.1) |
| Ever user | - | 189,631 (82.3) | 61,178 (91.2) | 128,453 (78.7) |
| Missing | | 444 (0.2) | 129 (0.2) | 315 (0.2) |
| Age at last live birth: n (%) ‡ | - | | | |
| No live births | - | 42,629 (18.5) | 16,983 (25.3) | 25,646 (15.7) |
| < 30 years | - | 89,363 (38.8) | 18,125 (27.0) | 71,238 (43.6) |
| ≥ 30 years | - | 97,781 (42.5) | 31,893 (47.5) | 65,888 (40.4) |
| Missing | | 553 (0.2) | 105 (0.2) | 448 (0.3) |

† – participants with missing values formed a separate category in the analysis; ‡ – female-specific variables (note that menopausal status was also used as a covariate in the models); n (%) – number of participants (percentage from total in cohort (for cohort size and cancer cases in men and women), or from total in women (for cohort size and cancer cases in pre- and post-menopausal women), or from total per column for categorical variables). The definition of variables is described in Supplementary Methods. Sun-exposure variables were used as covariates in the analyses for skin squamous-cell carcinoma and melanoma. Missing values were replaced with the sex-specific median category, except when marked with †.

## Supplementary Table S2 Allometric obesity indices in relation to cancer risk

| Cancer type<br>^ cancer subtype | Model | Cases | BMI<br>(per 5 kg/m$^2$)<br>(model A) | WHI<br>(per one SD)<br>(model A) | ABSI<br>(per one SD)<br>(model B) | HI<br>(per one SD)<br>(model B) |
|---|---|---|---|---|---|---|
| **Cancer groups** | | | | | | |
| Any cancer (overall) | Men | 14,682 | 1.01 (0.99 to 1.03) | 1.05 (1.03 to 1.07)** | 1.04 (1.02 to 1.06)** | 0.96 (0.94 to 0.97)** |
| | Women | 12,965 | 1.09 (1.07 to 1.11)** | 1.04 (1.03 to 1.06)** | 1.04 (1.02 to 1.05)** | 0.97 (0.95 to 0.99)** |
| | Pre-MP | 2,341 | 1.04 (1.00 to 1.09) | 0.98 (0.94 to 1.03) | 0.98 (0.94 to 1.02) | 1.00 (0.96 to 1.04) |
| | Post-MP | 10,624 | 1.11 (1.08 to 1.13)** | 1.06 (1.04 to 1.08)** | 1.05 (1.03 to 1.07)** | 0.97 (0.95 to 0.98)** |
| | $p_{interaction}$ | | <0.001 | <0.001 | <0.001 | 0.168 |
| ^ Obesity-related | Men | 3,316 | 1.20 (1.14 to 1.25)** | 1.13 (1.10 to 1.17)** | 1.13 (1.09 to 1.17)** | 0.92 (0.89 to 0.95)** |
| | Women | 7,668 | 1.19 (1.16 to 1.22)** | 1.04 (1.02 to 1.06)* | 1.03 (1.01 to 1.05)* | 0.97 (0.95 to 1.00)* |
| | Pre-MP | 728 | 1.19 (1.11 to 1.29)** | 1.04 (0.96 to 1.12) | 1.02 (0.94 to 1.10) | 0.96 (0.89 to 1.03) |
| | Post-MP | 6,940 | 1.19 (1.16 to 1.22)** | 1.04 (1.01 to 1.06)* | 1.03 (1.01 to 1.06)* | 0.97 (0.95 to 1.00)* |
| | $p_{interaction}$ | | 0.807 | 0.976 | 0.715 | 0.744 |
| ^ Non-obesity-related | Men | 11,366 | 0.96 (0.93 to 0.98)** | 1.03 (1.01 to 1.05)* | 1.02 (1.00 to 1.04) | 0.97 (0.95 to 0.99)* |
| | Women | 5,297 | 0.95 (0.93 to 0.98)* | 1.06 (1.03 to 1.09)** | 1.05 (1.02 to 1.08)* | 0.97 (0.94 to 0.99)* |
| | Pre-MP | 1,613 | 0.97 (0.92 to 1.03) | 0.96 (0.91 to 1.01) | 0.96 (0.91 to 1.01) | 1.02 (0.96 to 1.07) |
| | Post-MP | 3,684 | 0.95 (0.92 to 0.99)* | 1.09 (1.06 to 1.13)** | 1.08 (1.05 to 1.12)** | 0.94 (0.91 to 0.98)** |
| | $p_{interaction}$ | | 0.401 | <0.001 | <0.001 | 0.008 |
| **Head and neck** | | | | | | |
| Head and neck (overall) | Men | 412 | 0.91 (0.80 to 1.03) | 1.12 (1.02 to 1.23)* | 1.14 (1.03 to 1.26)* | 0.97 (0.88 to 1.08) |
| | Women | 164 | 0.85 (0.71 to 1.03) | 1.31 (1.13 to 1.51)** | 1.27 (1.10 to 1.48)* | 0.88 (0.75 to 1.02) |
| | Pre-MP | 31 | 1.01 (0.68 to 1.51) | 1.43 (1.01 to 2.02)* | 1.52 (1.07 to 2.17)* | 0.99 (0.68 to 1.43) |
| | Post-MP | 133 | 0.83 (0.67 to 1.02) | 1.29 (1.09 to 1.51)* | 1.22 (1.04 to 1.45)* | 0.85 (0.72 to 1.01) |
| | $p_{interaction}$ | | 0.561 | 0.740 | 0.315 | 0.387 |
| **Digestive system** | | | | | | |
| Oesophagus (overall) † | Men | 373 | 1.34 (1.19 to 1.52)** | 1.21 (1.10 to 1.33)** | 1.25 (1.12 to 1.39)** | 0.94 (0.85 to 1.04) |
| | Women | 115 | 0.73 (0.57 to 0.92)* | 1.20 (1.01 to 1.44)* | 1.21 (1.01 to 1.45)* | 0.95 (0.79 to 1.15) |
| ^ Oesophageal AC † | Men | 283 | 1.44 (1.26 to 1.66)** | 1.26 (1.13 to 1.40)** | 1.27 (1.12 to 1.44)** | 0.89 (0.79 to 1.00)* |
| | Women | 47 | 1.11 (0.81 to 1.52) | 0.93 (0.70 to 1.25) | 0.94 (0.70 to 1.26) | 1.03 (0.77 to 1.37) |
| ^ Oesophageal SCC † | Men | 46 | 0.81 (0.55 to 1.20) | 1.02 (0.76 to 1.36) | 1.16 (0.85 to 1.57) | 1.19 (0.97 to 1.46) |
| | Women | 60 | 0.43 (0.29 to 0.63)** | 1.35 (1.07 to 1.72)* | 1.37 (1.07 to 1.76)* | 0.91 (0.70 to 1.19) |
| Stomach † | Men | 220 | 1.24 (1.05 to 1.46)* | 1.26 (1.12 to 1.42)** | 1.24 (1.07 to 1.43)* | 0.83 (0.72 to 0.95)* |
| | Women | 97 | 1.07 (0.86 to 1.33) | 1.09 (0.89 to 1.32) | 1.03 (0.84 to 1.26) | 0.88 (0.72 to 1.07) |
| ^ Gastric cardia † | Men | 107 | 1.40 (1.11 to 1.76)* | 1.35 (1.12 to 1.63)* | 1.31 (1.07 to 1.61)* | 0.79 (0.65 to 0.96)* |
| | Women | 26 | 0.95 (0.61 to 1.47) | 0.99 (0.67 to 1.46) | 0.90 (0.60 to 1.33) | 0.84 (0.58 to 1.20) |
| ^ Gastric non-cardia † | Men | 113 | 1.10 (0.87 to 1.39) | 1.19 (1.00 to 1.42) | 1.17 (0.96 to 1.43) | 0.86 (0.71 to 1.04) |
| | Women | 71 | 1.11 (0.86 to 1.43) | 1.12 (0.89 to 1.41) | 1.08 (0.85 to 1.38) | 0.91 (0.72 to 1.14) |
| Colorectal (overall) | Men | 1,714 | 1.13 (1.07 to 1.21)** | 1.14 (1.09 to 1.20)** | 1.16 (1.10 to 1.22)** | 0.94 (0.89 to 0.99)* |
| | Women | 1,221 | 1.06 (1.00 to 1.13) | 1.07 (1.01 to 1.13)* | 1.07 (1.01 to 1.14)* | 0.98 (0.93 to 1.04) |
| | Pre-MP | 163 | 0.97 (0.82 to 1.15) | 1.12 (0.95 to 1.31) | 1.06 (0.90 to 1.24) | 0.87 (0.74 to 1.02) |
| | Post-MP | 1,058 | 1.08 (1.01 to 1.16)* | 1.06 (1.00 to 1.13)* | 1.08 (1.01 to 1.15)* | 1.00 (0.94 to 1.06) |
| | $p_{interaction}$ | | 0.190 | 0.479 | 0.754 | 0.100 |
| ^ Colon | Men | 1,029 | 1.19 (1.10 to 1.29)** | 1.16 (1.09 to 1.23)** | 1.18 (1.10 to 1.26)** | 0.92 (0.86 to 0.98)* |
| | Women | 889 | 1.09 (1.01 to 1.17)* | 1.07 (1.00 to 1.14)* | 1.08 (1.01 to 1.16)* | 0.99 (0.93 to 1.06) |
| | Pre-MP | 109 | 0.96 (0.78 to 1.19) | 1.13 (0.93 to 1.37) | 1.07 (0.88 to 1.31) | 0.87 (0.72 to 1.06) |
| | Post-MP | 780 | 1.11 (1.03 to 1.20)* | 1.06 (0.99 to 1.14) | 1.08 (1.01 to 1.16)* | 1.01 (0.94 to 1.08) |
| | $p_{interaction}$ | | 0.085 | 0.486 | 0.924 | 0.194 |
| ^ Rectum / RS junction | Men | 685 | 1.05 (0.95 to 1.16) | 1.11 (1.03 to 1.20)* | 1.13 (1.04 to 1.22)* | 0.97 (0.89 to 1.05) |
| | Women | 332 | 1.00 (0.88 to 1.13) | 1.07 (0.96 to 1.19) | 1.05 (0.94 to 1.18) | 0.96 (0.86 to 1.07) |
| | Pre-MP | 54 | 0.98 (0.74 to 1.32) | 1.09 (0.82 to 1.44) | 1.01 (0.76 to 1.35) | 0.86 (0.66 to 1.13) |
| | Post-MP | 278 | 1.00 (0.87 to 1.15) | 1.06 (0.94 to 1.20) | 1.06 (0.94 to 1.20) | 0.98 (0.87 to 1.10) |
| | $p_{interaction}$ | | 0.706 | 0.808 | 0.713 | 0.328 |
| Liver and gallbladder † | Men | 215 | 1.39 (1.18 to 1.63)** | 1.15 (1.01 to 1.31)* | 1.10 (0.95 to 1.27) | 0.86 (0.75 to 0.98)* |
| | Women | 155 | 1.08 (0.91 to 1.29) | 1.05 (0.90 to 1.23) | 1.10 (0.94 to 1.29) | 1.06 (0.91 to 1.23) |
| Pancreas | Men | 301 | 1.10 (0.95 to 1.27) | 1.04 (0.92 to 1.16) | 1.04 (0.92 to 1.18) | 0.99 (0.88 to 1.11) |
| | Women | 241 | 1.20 (1.04 to 1.37)* | 1.13 (1.00 to 1.28) | 1.09 (0.96 to 1.25) | 0.90 (0.80 to 1.02) |
| | Pre-MP | 22 | 1.95 (1.35 to 2.82)** | 1.39 (0.93 to 2.09) | 1.35 (0.88 to 2.07) | 0.82 (0.57 to 1.20) |
| | Post-MP | 219 | 1.11 (0.96 to 1.29) | 1.11 (0.97 to 1.26) | 1.07 (0.94 to 1.23) | 0.91 (0.80 to 1.04) |
| *(continues)* | $p_{interaction}$ | | 0.013 | 0.353 | 0.182 | 0.594 |

| **Cancer type**<br>^ cancer subtype | **Model** | **Cases** | **BMI**<br>(per 5 kg/m$^2$)<br>(model A) | **WHI**<br>(per one SD)<br>(model A) | **ABSI**<br>(per one SD)<br>(model B) | **HI**<br>(per one SD)<br>(model B) |
|---|---|---|---|---|---|---|
| **Respiratory system** | | | | | | |
| Lung (overall) | Men | 1,007 | 0.85 (0.78 to 0.92)** | 1.20 (1.13 to 1.27)** | 1.24 (1.16 to 1.33)** | 0.95 (0.89 to 1.02) |
| | Women | 875 | 0.93 (0.86 to 1.01) | 1.19 (1.12 to 1.27)** | 1.24 (1.16 to 1.32)** | 1.00 (0.94 to 1.07) |
| | Pre-MP | 54 | 1.06 (0.81 to 1.40) | 1.39 (1.07 to 1.80)* | 1.37 (1.04 to 1.81)* | 0.88 (0.67 to 1.14) |
| | Post-MP | 821 | 0.92 (0.85 to 1.00)* | 1.18 (1.10 to 1.26)** | 1.23 (1.15 to 1.31)** | 1.01 (0.95 to 1.08) |
| | $p_{interaction}$ | | 0.281 | 0.364 | 0.445 | 0.457 |
| ^ Lung AC | Men | 334 | 0.85 (0.74 to 0.99)* | 1.15 (1.03 to 1.28)* | 1.16 (1.03 to 1.30)* | 0.93 (0.83 to 1.04) |
| | Women | 398 | 0.85 (0.76 to 0.96)* | 1.15 (1.04 to 1.26)* | 1.17 (1.06 to 1.29)* | 0.99 (0.90 to 1.09) |
| | Pre-MP | 24 | 0.96 (0.61 to 1.51) | 1.41 (0.94 to 2.10) | 1.33 (0.88 to 2.01) | 0.82 (0.55 to 1.23) |
| | Post-MP | 374 | 0.85 (0.75 to 0.96)* | 1.13 (1.02 to 1.25)* | 1.16 (1.05 to 1.28)* | 1.00 (0.90 to 1.11) |
| | $p_{interaction}$ | | 0.717 | 0.399 | 0.588 | 0.430 |
| ^ Lung SCC † | Men | 256 | 0.92 (0.79 to 1.08) | 1.27 (1.14 to 1.42)** | 1.33 (1.17 to 1.52)** | 0.92 (0.81 to 1.04) |
| | Women | 129 | 0.86 (0.70 to 1.05) | 1.27 (1.08 to 1.49)* | 1.40 (1.20 to 1.63)** | 1.10 (0.94 to 1.29) |
| ^ Lung small cell CA † | Men | 113 | 0.83 (0.65 to 1.06) | 1.15 (0.96 to 1.37) | 1.10 (0.91 to 1.33) | 0.86 (0.71 to 1.04) |
| | Women | 96 | 1.24 (1.01 to 1.54)* | 1.27 (1.05 to 1.54)* | 1.39 (1.14 to 1.69)* | 1.04 (0.86 to 1.26) |
| **Urinary system** | | | | | | |
| Kidney (overall) | Men | 435 | 1.41 (1.26 to 1.57)** | 1.08 (0.98 to 1.18) | 1.04 (0.94 to 1.16) | 0.91 (0.82 to 1.00) |
| | Women | 226 | 1.43 (1.25 to 1.63)** | 1.28 (1.13 to 1.45)** | 1.17 (1.02 to 1.33)* | 0.80 (0.71 to 0.91)** |
| | Pre-MP | 28 | 1.72 (1.24 to 2.40)* | 1.23 (0.85 to 1.76) | 1.32 (0.91 to 1.92) | 1.07 (0.76 to 1.52) |
| | Post-MP | 198 | 1.37 (1.19 to 1.59)** | 1.29 (1.13 to 1.47)** | 1.15 (0.99 to 1.32) | 0.77 (0.67 to 0.88)** |
| | $p_{interaction}$ | | 0.190 | 0.740 | 0.254 | 0.035 |
| ^ Clear cell AC † | Men | 199 | 1.47 (1.25 to 1.74)** | 1.08 (0.94 to 1.25) | 1.04 (0.89 to 1.21) | 0.89 (0.77 to 1.03) |
| | Women | 111 | 1.52 (1.26 to 1.83)** | 1.36 (1.14 to 1.62)** | 1.25 (1.03 to 1.50)* | 0.78 (0.66 to 0.93)* |
| ^ Renal cell CA † | Men | 157 | 1.45 (1.21 to 1.75)** | 1.10 (0.94 to 1.29) | 1.07 (0.90 to 1.28) | 0.91 (0.77 to 1.07) |
| | Women | 89 | 1.39 (1.13 to 1.72)* | 1.19 (0.98 to 1.46) | 1.08 (0.87 to 1.33) | 0.83 (0.68 to 1.01) |
| Bladder † | Men | 450 | 1.09 (0.97 to 1.23) | 1.14 (1.04 to 1.26)* | 1.15 (1.04 to 1.27)* | 0.93 (0.85 to 1.03) |
| | Women | 116 | 1.14 (0.94 to 1.39) | 1.12 (0.94 to 1.34) | 1.10 (0.92 to 1.32) | 0.92 (0.77 to 1.09) |
| **Reproductive system** | | | | | | |
| Prostate | Men | 5,488 | 0.92 (0.89 to 0.96)** | 1.01 (0.98 to 1.04) | 1.00 (0.97 to 1.03) | 0.98 (0.95 to 1.00) |
| Breast (overall) | Women | 5,178 | 1.11 (1.08 to 1.15)** | 1.01 (0.98 to 1.03) | 1.00 (0.97 to 1.03) | 0.98 (0.96 to 1.01) |
| ^ Breast (pre-menopausal) † | Women | 1,055 | 0.98 (0.92 to 1.05) | 0.95 (0.89 to 1.01) | 0.95 (0.89 to 1.01) | 1.02 (0.96 to 1.09) |
| ^ Breast (post-menopausal) | Women | 4,123 | 1.16 (1.12 to 1.19)** | 1.02 (0.99 to 1.05) | 1.01 (0.98 to 1.04) | 0.97 (0.94 to 1.00) |
| | Pre-MP | 241 | 1.12 (0.98 to 1.28) | 1.06 (0.93 to 1.21) | 1.03 (0.90 to 1.18) | 0.92 (0.81 to 1.05) |
| | Post-MP | 3,882 | 1.16 (1.12 to 1.20)** | 1.02 (0.99 to 1.05) | 1.01 (0.98 to 1.04) | 0.98 (0.95 to 1.01) |
| | $p_{interaction}$ | | 0.395 | 0.436 | 0.825 | 0.436 |
| Ovary | Women | 594 | 1.06 (0.97 to 1.16) | 0.98 (0.90 to 1.07) | 0.99 (0.91 to 1.07) | 1.01 (0.93 to 1.10) |
| | Pre-MP | 97 | 1.19 (0.98 to 1.45) | 0.96 (0.78 to 1.18) | 0.99 (0.80 to 1.23) | 1.07 (0.87 to 1.31) |
| | Post-MP | 497 | 1.03 (0.93 to 1.14) | 0.99 (0.90 to 1.08) | 0.99 (0.90 to 1.08) | 1.00 (0.92 to 1.10) |
| | $p_{interaction}$ | | 0.301 | 0.525 | 0.853 | 0.486 |
| Endometrium | Women | 699 | 1.80 (1.68 to 1.92)** | 1.08 (1.00 to 1.16)* | 1.08 (1.00 to 1.17)* | 0.99 (0.92 to 1.06) |
| | Pre-MP | 104 | 1.47 (1.24 to 1.75)** | 0.95 (0.78 to 1.16) | 0.93 (0.76 to 1.15) | 1.01 (0.83 to 1.22) |
| | Post-MP | 595 | 1.86 (1.73 to 2.01)** | 1.10 (1.01 to 1.19)* | 1.11 (1.02 to 1.20)* | 0.98 (0.91 to 1.06) |
| | $p_{interaction}$ | | 0.064 | 0.264 | 0.084 | 0.869 |
| **Skin** | | | | | | |
| Skin SCC | Men | 953 | 0.91 (0.83 to 0.99)* | 0.98 (0.92 to 1.05) | 0.96 (0.89 to 1.02) | 0.96 (0.89 to 1.02) |
| | Women | 561 | 0.84 (0.76 to 0.94)* | 1.11 (1.02 to 1.21)* | 1.07 (0.99 to 1.17) | 0.91 (0.83 to 0.99)* |
| | Pre-MP | 32 | 0.60 (0.37 to 0.97)* | 1.04 (0.71 to 1.50) | 0.95 (0.65 to 1.40) | 0.85 (0.58 to 1.23) |
| | Post-MP | 529 | 0.86 (0.77 to 0.96)* | 1.12 (1.02 to 1.21)* | 1.08 (0.99 to 1.18) | 0.91 (0.84 to 0.99)* |
| | $p_{interaction}$ | | 0.118 | 0.970 | 0.488 | 0.525 |
| Melanoma | Men | 750 | 1.19 (1.09 to 1.31)** | 0.91 (0.84 to 0.98)* | 0.89 (0.82 to 0.96)* | 1.00 (0.93 to 1.08) |
| | Women | 736 | 0.97 (0.89 to 1.05) | 0.96 (0.89 to 1.04) | 0.92 (0.85 to 1.00)* | 0.93 (0.87 to 1.01) |
| | Pre-MP | 176 | 0.89 (0.74 to 1.06) | 0.78 (0.66 to 0.91)* | 0.77 (0.65 to 0.91)* | 1.06 (0.91 to 1.24) |
| | Post-MP | 560 | 0.99 (0.90 to 1.09) | 1.02 (0.94 to 1.11) | 0.97 (0.89 to 1.06) | 0.90 (0.83 to 0.98)* |
| | $p_{interaction}$ | | 0.108 | 0.004 | 0.006 | 0.185 |

| Cancer type<br>^ cancer subtype | Model | Cases | BMI (per 5 kg/m²) (model A) | WHI (per one SD) (model A) | ABSI (per one SD) (model B) | HI (per one SD) (model B) |
|---|---|---|---|---|---|---|
| **Nervous and endocrine** | | | | | | |
| Brain | Men | 266 | 0.92 (0.78 to 1.08) | 0.97 (0.86 to 1.10) | 0.94 (0.83 to 1.08) | 0.96 (0.84 to 1.09) |
| | Women | 167 | 1.00 (0.84 to 1.19) | 1.14 (0.98 to 1.33) | 1.11 (0.95 to 1.29) | 0.89 (0.76 to 1.03) |
| | Pre-MP | 25 | 1.15 (0.77 to 1.72) | 1.23 (0.82 to 1.84) | 1.04 (0.68 to 1.59) | 0.69 (0.47 to 1.01) |
| | Post-MP | 142 | 0.97 (0.80 to 1.18) | 1.14 (0.96 to 1.34) | 1.12 (0.95 to 1.32) | 0.92 (0.78 to 1.09) |
| | $p_{interaction}$ | | 0.291 | 0.984 | 0.596 | 0.269 |
| Thyroid | Men | 54 | 0.86 (0.60 to 1.22) | 1.20 (0.96 to 1.49) | 1.30 (0.97 to 1.73) | 0.97 (0.73 to 1.30) |
| | Women | 145 | 1.13 (0.95 to 1.35) | 0.98 (0.83 to 1.16) | 0.94 (0.79 to 1.12) | 0.93 (0.79 to 1.09) |
| | Pre-MP | 39 | 1.06 (0.76 to 1.48) | 0.88 (0.63 to 1.24) | 0.94 (0.67 to 1.33) | 1.17 (0.84 to 1.64) |
| | Post-MP | 106 | 1.18 (0.96 to 1.46) | 1.03 (0.85 to 1.24) | 0.95 (0.78 to 1.16) | 0.86 (0.71 to 1.03) |
| | $p_{interaction}$ | | 0.492 | 0.432 | 0.856 | 0.153 |
| **Hematopoietic system** | | | | | | |
| Leukaemia | Men | 378 | 0.98 (0.86 to 1.12) | 0.95 (0.86 to 1.06) | 0.94 (0.84 to 1.05) | 1.01 (0.90 to 1.12) |
| | Women | 234 | 1.12 (0.97 to 1.29) | 0.98 (0.86 to 1.11) | 0.95 (0.83 to 1.08) | 0.96 (0.85 to 1.09) |
| | Pre-MP | 30 | 1.47 (1.03 to 2.09)* | 0.92 (0.63 to 1.35) | 0.93 (0.64 to 1.37) | 1.02 (0.71 to 1.47) |
| | Post-MP | 204 | 1.08 (0.92 to 1.25) | 0.98 (0.86 to 1.13) | 0.95 (0.83 to 1.10) | 0.95 (0.83 to 1.09) |
| | $p_{interaction}$ | | 0.300 | 0.852 | 0.878 | 0.812 |
| Multiple myeloma | Men | 207 | 0.90 (0.74 to 1.08) | 1.04 (0.90 to 1.19) | 0.97 (0.83 to 1.12) | 0.86 (0.75 to 1.00)* |
| | Women | 174 | 1.13 (0.96 to 1.33) | 1.04 (0.89 to 1.20) | 0.98 (0.84 to 1.15) | 0.90 (0.78 to 1.04) |
| | Pre-MP | 23 | 0.91 (0.57 to 1.47) | 0.92 (0.59 to 1.43) | 0.84 (0.53 to 1.32) | 0.88 (0.58 to 1.35) |
| | Post-MP | 151 | 1.17 (0.99 to 1.39) | 1.05 (0.90 to 1.23) | 1.00 (0.85 to 1.18) | 0.90 (0.77 to 1.05) |
| | $p_{interaction}$ | | 0.231 | 0.731 | 0.535 | 0.98 |
| Non-Hodgkin lymphoma | Men | 571 | 1.03 (0.93 to 1.15) | 0.99 (0.90 to 1.07) | 0.93 (0.85 to 1.02) | 0.92 (0.84 to 1.01) |
| | Women | 490 | 1.00 (0.90 to 1.10) | 1.03 (0.95 to 1.13) | 1.04 (0.94 to 1.13) | 0.99 (0.91 to 1.08) |
| | Pre-MP | 65 | 0.92 (0.70 to 1.21) | 1.07 (0.83 to 1.37) | 1.13 (0.87 to 1.46) | 1.08 (0.85 to 1.39) |
| | Post-MP | 425 | 1.01 (0.91 to 1.13) | 1.03 (0.94 to 1.14) | 1.02 (0.93 to 1.13) | 0.98 (0.89 to 1.07) |
| | $p_{interaction}$ | | 0.745 | 0.877 | 0.510 | 0.415 |

**†** – cancers with less than 20 cases in women pre-menopausal at baseline, for which models were not adjusted for menopausal status; **ABSI** – a body shape index; **AC** – adenocarcinoma; **BMI** – body mass index; **CA** – carcinoma; **HI** – hip index; **MP** – menopausal; **RS** – recto-sigmoid; **SCC** – squamous cell carcinoma; **SD** – standard deviation; **WHI** – waist-to-hip index; Hazard ratios (95% confidence intervals) were obtained from delayed entry Cox proportional hazards models stratified by age at baseline and region of the assessment centre. **Model A** – included BMI and WHI with adjustment variables. **Model B** – included BMI, ABSI and HI with adjustment variables (HR estimates for BMI in Model A and Model B were similar). **Men** – models adjusted for height, weight change during last year preceding baseline, Townsend deprivation index, smoking status, alcohol consumption, physical activity, consumption of fresh fruit and vegetables, processed meat and red meat, family history of cancer and, for skin SCC and melanoma, sun-exposure-related factors. **Women** – models included adjustment variables as for men, with the addition of menopausal status (except for cancers marked with #), use of hormone replacement therapy, ever use of oral contraceptives and age at last live birth (with "no live births" as one of the categories). **Pre-MP** – for the models, this applies to the sub-group of women pre-menopausal at baseline, with adjustment as in models for women. **Pre-menopausal** – applies to breast cancers diagnosed below 55 years of age in women pre-menopausal at baseline. **Post-MP** – for the models, this applies to the sub-

group of women post-menopausal at baseline, with adjustment as in models for women. **Post-menopausal** – applies to breast cancers diagnosed at age 55 years or older, irrespective of menopausal status at baseline, i.e. women diagnosed with post-menopausal breast cancer, could be pre- or post-menopausal at baseline. $\mathbf{p_{interaction}}$ – p-value corresponding to the Wald test for the interaction term between menopausal status at baseline and each obesity index, included in adjusted models for women (Model A for BMI or WHI and Model B for ABSI or HI, one interaction at a time); Cancer types and subtypes are defined in Supplementary Methods according to the 10$^{th}$ edition of the International Statistical Classification of Diseases (ICD10). Obesity-related cancers included oesophageal adenocarcinoma, cancers of the gastric cardia, colon, rectum and rectosigmoid junction, liver, gallbladder and bile ducts, pancreas, kidney, postmenopausal breast, ovary, endometrium, thyroid and multiple myeloma. Non-obesity-related cancers included the remaining cancers. * p-value < 0.05; ** p-value < 0.001.

## Supplementary Table S3 Allometric obesity index categories in relation to cancer risk

| Cancer type / ^ subtype | Model | Cat | **BMI** (model A) Cases | **BMI** (model A) HR (95% CI) | **WHI** (model A) Cases | **WHI** (model A) HR (95% CI) | **ABSI** (model B) Cases | **ABSI** (model B) HR (95% CI) | **HI** (model B) Cases | **HI** (model B) HR (95% CI) |
|---|---|---|---|---|---|---|---|---|---|---|
| Any cancer (overall) | Men | T1 | 3,459 | reference | 3,922 | reference | 3,876 | reference | 4,833 | reference |
| | | T2 | 7,374 | 1.01 (0.97 to 1.05) | 4,941 | 1.07 (1.02 to 1.12)* | 4,756 | 1.00 (0.96 to 1.05) | 4,852 | 0.96 (0.92 to 1.00)* |
| | | T3 | 3,849 | 1.03 (0.98 to 1.08) | 5,819 | 1.10 (1.05 to 1.14)** | 6,050 | 1.07 (1.02 to 1.11)* | 4,997 | 0.91 (0.87 to 0.95)** |
| | Women | T1 | 4,638 | reference | 3,865 | reference | 3,748 | reference | 4,373 | reference |
| | | T2 | 5,029 | 1.08 (1.03 to 1.12)** | 4,304 | 1.05 (1.00 to 1.10)* | 4,296 | 1.04 (1.00 to 1.09) | 4,308 | 0.98 (0.94 to 1.02) |
| | | T3 | 3,298 | 1.18 (1.12 to 1.23)** | 4,796 | 1.09 (1.04 to 1.14)** | 4,921 | 1.08 (1.03 to 1.13)** | 4,284 | 0.94 (0.90 to 0.98)* |
| | Pre-MP | T1 | 1,061 | reference | 951 | reference | 968 | reference | 830 | reference |
| | | T2 | 798 | 1.06 (0.96 to 1.16) | 761 | 0.97 (0.88 to 1.06) | 809 | 1.04 (0.95 to 1.15) | 796 | 0.98 (0.88 to 1.08) |
| | | T3 | 482 | 1.02 (0.91 to 1.15) | 629 | 1.01 (0.91 to 1.12) | 564 | 1.00 (0.90 to 1.11) | 715 | 0.97 (0.88 to 1.07) |
| | Post-MP | T1 | 3,577 | reference | 2,914 | reference | 2,780 | reference | 3,543 | reference |
| | | T2 | 4,231 | 1.09 (1.04 to 1.14)** | 3,543 | 1.07 (1.02 to 1.13)* | 3,487 | 1.05 (0.99 to 1.10) | 3,512 | 0.98 (0.94 to 1.03) |
| | | T3 | 2,816 | 1.22 (1.15 to 1.28)** | 4,167 | 1.11 (1.06 to 1.17)** | 4,357 | 1.09 (1.04 to 1.15)** | 3,569 | 0.93 (0.89 to 0.98)* |
| Obesity-related (overall) | Men | T1 | 648 | reference | 789 | reference | 822 | reference | 1,194 | reference |
| | | T2 | 1,599 | 1.13 (1.03 to 1.24)* | 1,102 | 1.17 (1.07 to 1.28)** | 1,037 | 1.03 (0.94 to 1.13) | 1,059 | 0.89 (0.81 to 0.96)* |
| | | T3 | 1,069 | 1.43 (1.29 to 1.59)** | 1,425 | 1.30 (1.19 to 1.43)** | 1,457 | 1.23 (1.12 to 1.35)** | 1,063 | 0.85 (0.78 to 0.93)** |
| | Women | T1 | 2,481 | reference | 2,273 | reference | 2,149 | reference | 2,568 | reference |
| | | T2 | 3,035 | 1.20 (1.14 to 1.27)** | 2,549 | 1.04 (0.98 to 1.10) | 2,520 | 1.02 (0.96 to 1.08) | 2,508 | 0.97 (0.92 to 1.03) |
| | | T3 | 2,152 | 1.43 (1.35 to 1.53)** | 2,846 | 1.07 (1.01 to 1.13)* | 2,999 | 1.06 (1.00 to 1.12)* | 2,592 | 0.94 (0.89 to 1.00)* |
| | Pre-MP | T1 | 274 | reference | 271 | reference | 267 | reference | 275 | reference |
| | | T2 | 259 | 1.29 (1.08 to 1.54)* | 247 | 1.07 (0.90 to 1.28) | 269 | 1.18 (0.99 to 1.40) | 236 | 0.88 (0.73 to 1.04) |
| | | T3 | 195 | 1.49 (1.23 to 1.82)** | 210 | 1.10 (0.92 to 1.32) | 192 | 1.06 (0.88 to 1.29) | 217 | 0.88 (0.73 to 1.05) |
| | Post-MP | T1 | 2,207 | reference | 2,002 | reference | 1,882 | reference | 2,293 | reference |
| | | T2 | 2,776 | 1.19 (1.13 to 1.27)** | 2,302 | 1.04 (0.98 to 1.10) | 2,251 | 1.00 (0.94 to 1.07) | 2,272 | 0.99 (0.93 to 1.05) |
| | | T3 | 1,957 | 1.43 (1.34 to 1.52)** | 2,636 | 1.06 (1.00 to 1.13)* | 2,807 | 1.06 (0.99 to 1.12) | 2,375 | 0.95 (0.90 to 1.01) |
| Non-obesity-related (overall) | Men | T1 | 2,811 | reference | 3,133 | reference | 3,054 | reference | 3,639 | reference |
| | | T2 | 5,775 | 0.98 (0.94 to 1.03) | 3,839 | 1.04 (0.99 to 1.09) | 3,719 | 1.00 (0.95 to 1.05) | 3,793 | 0.98 (0.93 to 1.03) |
| | | T3 | 2,780 | 0.93 (0.88 to 0.98)* | 4,394 | 1.04 (1.00 to 1.10) | 4,593 | 1.03 (0.98 to 1.08) | 3,934 | 0.93 (0.88 to 0.97)* |
| | Women | T1 | 2,157 | reference | 1,592 | reference | 1,599 | reference | 1,805 | reference |
| | | T2 | 1,994 | 0.93 (0.87 to 0.99)* | 1,755 | 1.06 (0.99 to 1.14) | 1,776 | 1.07 (1.00 to 1.15)* | 1,800 | 0.99 (0.93 to 1.06) |
| | | T3 | 1,146 | 0.88 (0.81 to 0.95)** | 1,950 | 1.12 (1.05 to 1.20)** | 1,922 | 1.10 (1.03 to 1.18)* | 1,692 | 0.93 (0.87 to 1.00)* |
| | Pre-MP | T1 | 787 | reference | 680 | reference | 701 | reference | 555 | reference |
| | | T2 | 539 | 0.97 (0.87 to 1.09) | 514 | 0.92 (0.82 to 1.03) | 540 | 0.99 (0.88 to 1.10) | 560 | 1.03 (0.91 to 1.16) |
| | | T3 | 287 | 0.85 (0.74 to 0.98)* | 419 | 0.97 (0.86 to 1.10) | 372 | 0.97 (0.85 to 1.10) | 498 | 1.01 (0.90 to 1.15) |
| | Post-MP | T1 | 1,370 | reference | 912 | reference | 898 | reference | 1,250 | reference |
| | | T2 | 1,455 | 0.92 (0.85 to 1.00)* | 1,241 | 1.16 (1.06 to 1.26)** | 1,236 | 1.14 (1.04 to 1.24)* | 1,240 | 0.98 (0.90 to 1.06) |
| | | T3 | 859 | 0.90 (0.82 to 0.98)* | 1,531 | 1.21 (1.12 to 1.32)** | 1,550 | 1.17 (1.07 to 1.27)** | 1,194 | 0.89 (0.82 to 0.97)* |
| Head & neck (overall) | Men | T1 | 127 | reference | 110 | reference | 115 | reference | 146 | reference |
| | | T2 | 175 | 0.69 (0.55 to 0.88)* | 121 | 0.97 (0.75 to 1.26) | 120 | 0.95 (0.73 to 1.23) | 133 | 0.89 (0.70 to 1.13) |
| | | T3 | 110 | 0.80 (0.61 to 1.05) | 181 | 1.25 (0.97 to 1.59) | 177 | 1.22 (0.95 to 1.56) | 133 | 0.81 (0.63 to 1.04) |
| | Women | T1 | 63 | reference | 39 | reference | 39 | reference | 62 | reference |
| | | T2 | 63 | 0.91 (0.64 to 1.31) | 49 | 1.12 (0.74 to 1.71) | 55 | 1.30 (0.86 to 1.97) | 60 | 1.01 (0.71 to 1.45) |
| | | T3 | 38 | 0.89 (0.58 to 1.36) | 76 | 1.57 (1.06 to 2.33)* | 70 | 1.52 (1.01 to 2.28)* | 42 | 0.73 (0.49 to 1.08) |
| | Post-MP | T1 | 50 | reference | 29 | reference | 30 | reference | 51 | reference |
| | | T2 | 53 | 0.92 (0.61 to 1.37) | 42 | 1.21 (0.75 to 1.95) | 44 | 1.25 (0.78 to 2.00) | 48 | 0.99 (0.66 to 1.47) |
| | | T3 | 30 | 0.84 (0.52 to 1.35) | 62 | 1.52 (0.97 to 2.37) | 59 | 1.42 (0.90 to 2.24) | 34 | 0.69 (0.45 to 1.08) |
| Oesophagus (overall) † | Men | T1 | 56 | reference | 81 | reference | 74 | reference | 138 | reference |
| | | T2 | 182 | 1.44 (1.06 to 1.95)* | 117 | 1.14 (0.86 to 1.52) | 118 | 1.26 (0.94 to 1.70) | 113 | 0.84 (0.65 to 1.09) |
| | | T3 | 135 | 1.88 (1.35 to 2.60)** | 175 | 1.36 (1.04 to 1.79)* | 181 | 1.58 (1.19 to 2.11)* | 122 | 0.90 (0.69 to 1.16) |
| | Women | T1 | 47 | reference | 22 | reference | 25 | reference | 40 | reference |
| | | T2 | 50 | 1.01 (0.67 to 1.52) | 42 | 1.63 (0.97 to 2.73) | 40 | 1.40 (0.85 to 2.32) | 38 | 0.95 (0.61 to 1.49) |
| | | T3 | 18 | 0.60 (0.34 to 1.07) | 51 | 1.68 (1.01 to 2.79)* | 50 | 1.52 (0.93 to 2.50) | 37 | 0.95 (0.60 to 1.50) |
| | Post-MP | T1 | 46 | reference | 21 | reference | 25 | reference | 40 | reference |
| | | T2 | 46 | 0.92 (0.61 to 1.41) | 40 | 1.59 (0.94 to 2.70) | 37 | 1.25 (0.75 to 2.09) | 34 | 0.83 (0.53 to 1.32) |
| | | T3 | 17 | 0.57 (0.32 to 1.03) | 48 | 1.58 (0.94 to 2.66) | 47 | 1.35 (0.82 to 2.22) | 35 | 0.88 (0.55 to 1.39) |
| ^ Oesophageal adeno-carcinoma † | Men | T1 | 39 | reference | 62 | reference | 56 | reference | 110 | reference |
| | | T2 | 136 | 1.55 (1.08 to 2.22)* | 85 | 1.07 (0.77 to 1.49) | 91 | 1.28 (0.91 to 1.80) | 88 | 0.83 (0.62 to 1.11) |
| | | T3 | 108 | 2.19 (1.49 to 3.20)** | 136 | 1.37 (1.00 to 1.86)* | 136 | 1.57 (1.13 to 2.17)* | 85 | 0.80 (0.59 to 1.08) |
| | Women | T1 | 14 | reference | 10 | reference | 14 | reference | 15 | reference |
| | | T2 | 23 | 1.57 (0.79 to 3.12) | 23 | 1.92 (0.91 to 4.04) | 15 | 0.84 (0.40 to 1.75) | 13 | 0.89 (0.42 to 1.87) |
| | | T3 | 10 | 1.13 (0.48 to 2.68) | 14 | 0.96 (0.42 to 2.18) | 18 | 0.79 (0.38 to 1.62) | 19 | 1.27 (0.63 to 2.54) |
| | Post-MP | T1 | 14 | reference | 10 | reference | 14 | reference | 15 | reference |
| | | T2 | 22 | 1.47 (0.74 to 2.93) | 22 | 1.80 (0.85 to 3.80) | 14 | 0.76 (0.36 to 1.61) | 13 | 0.88 (0.42 to 1.86) |
| | | T3 | 10 | 1.10 (0.47 to 2.61) | 14 | 0.92 (0.40 to 2.09) | 18 | 0.74 (0.36 to 1.53) | 18 | 1.19 (0.59 to 2.40) |

| Cancer type / ^ subtype | Model | Cat | **BMI** (model A) Cases | HR (95% CI) | **WHI** (model A) Cases | HR (95% CI) | **ABSI** (model B) Cases | HR (95% CI) | **HI** (model B) Cases | HR (95% CI) |
|---|---|---|---|---|---|---|---|---|---|---|
| Stomach (overall) † | Men | T1 | 39 | reference | 46 | reference | 52 | reference | 85 | reference |
| | | T2 | 113 | 1.32 (0.91 to 1.91) | 75 | 1.35 (0.93 to 1.95) | 60 | 0.96 (0.66 to 1.40) | 68 | 0.79 (0.57 to 1.09) |
| | | T3 | 68 | 1.44 (0.95 to 2.18) | 99 | 1.50 (1.04 to 2.14)* | 108 | 1.44 (1.01 to 2.05)* | 67 | 0.72 (0.51 to 1.01) |
| | Women | T1 | 37 | reference | 26 | reference | 20 | reference | 38 | reference |
| | | T2 | 35 | 0.84 (0.52 to 1.35) | 36 | 1.24 (0.75 to 2.06) | 41 | 1.83 (1.07 to 3.14)* | 28 | 0.73 (0.45 to 1.19) |
| | | T3 | 25 | 0.93 (0.54 to 1.61) | 35 | 1.06 (0.63 to 1.78) | 36 | 1.37 (0.78 to 2.41) | 31 | 0.76 (0.47 to 1.24) |
| | Post-MP | T1 | 33 | reference | 21 | reference | 17 | reference | 34 | reference |
| | | T2 | 31 | 0.78 (0.47 to 1.29) | 33 | 1.34 (0.77 to 2.32) | 37 | 1.87 (1.05 to 3.34)* | 22 | 0.62 (0.36 to 1.07) |
| | | T3 | 20 | 0.76 (0.42 to 1.37) | 30 | 1.05 (0.59 to 1.85) | 30 | 1.24 (0.67 to 2.29) | 28 | 0.73 (0.44 to 1.22) |
| ^ Gastric cardia † | Men | T1 | 18 | reference | 22 | reference | 27 | reference | 45 | reference |
| | | T2 | 53 | 1.39 (0.81 to 2.40) | 33 | 1.21 (0.70 to 2.08) | 22 | 0.66 (0.37 to 1.16) | 29 | 0.64 (0.40 to 1.03) |
| | | T3 | 36 | 1.82 (1.00 to 3.29)* | 52 | 1.58 (0.95 to 2.63) | 58 | 1.42 (0.87 to 2.31) | 33 | 0.67 (0.41 to 1.09) |
| ^ Gastric non-cardia † | Men | T1 | 21 | reference | 24 | reference | 25 | reference | 40 | reference |
| | | T2 | 60 | 1.24 (0.75 to 2.05) | 42 | 1.48 (0.89 to 2.46) | 38 | 1.29 (0.77 to 2.16) | 39 | 0.95 (0.60 to 1.49) |
| | | T3 | 32 | 1.15 (0.64 to 2.05) | 47 | 1.41 (0.85 to 2.34) | 50 | 1.45 (0.87 to 2.42) | 34 | 0.77 (0.47 to 1.26) |
| | Women | T1 | 26 | reference | 20 | reference | 14 | reference | 28 | reference |
| | | T2 | 25 | 0.88 (0.50 to 1.54) | 25 | 1.15 (0.64 to 2.07) | 28 | 1.81 (0.95 to 3.46) | 18 | 0.64 (0.35 to 1.17) |
| | | T3 | 20 | 1.14 (0.61 to 2.12) | 26 | 1.06 (0.58 to 1.92) | 29 | 1.61 (0.83 to 3.12) | 25 | 0.85 (0.49 to 1.47) |
| | Post-MP | T1 | 23 | reference | 15 | reference | 11 | reference | 24 | reference |
| | | T2 | 22 | 0.81 (0.44 to 1.47) | 23 | 1.34 (0.70 to 2.57) | 25 | 1.97 (0.96 to 4.02) | 14 | 0.55 (0.29 to 1.08) |
| | | T3 | 15 | 0.87 (0.43 to 1.73) | 22 | 1.12 (0.57 to 2.18) | 24 | 1.58 (0.76 to 3.30) | 22 | 0.80 (0.44 to 1.45) |
| Colorectal (overall) | Men | T1 | 345 | reference | 399 | reference | 412 | reference | 601 | reference |
| | | T2 | 851 | 1.11 (0.97 to 1.26) | 585 | 1.24 (1.09 to 1.41)** | 540 | 1.08 (0.95 to 1.23) | 554 | 0.90 (0.80 to 1.01) |
| | | T3 | 518 | 1.29 (1.11 to 1.48)** | 730 | 1.35 (1.19 to 1.53)** | 762 | 1.32 (1.16 to 1.49)** | 559 | 0.87 (0.76 to 0.98)* |
| | Women | T1 | 442 | reference | 343 | reference | 336 | reference | 398 | reference |
| | | T2 | 484 | 1.07 (0.94 to 1.23) | 428 | 1.15 (1.00 to 1.32) | 412 | 1.09 (0.94 to 1.26) | 405 | 0.99 (0.86 to 1.14) |
| | | T3 | 295 | 1.09 (0.93 to 1.27) | 450 | 1.12 (0.97 to 1.29) | 473 | 1.11 (0.96 to 1.28) | 418 | 0.97 (0.84 to 1.11) |
| | Pre-MP | T1 | 75 | reference | 60 | reference | 65 | reference | 65 | reference |
| | | T2 | 53 | 0.98 (0.68 to 1.40) | 54 | 1.06 (0.73 to 1.53) | 56 | 1.03 (0.72 to 1.48) | 54 | 0.83 (0.58 to 1.20) |
| | | T3 | 35 | 1.00 (0.65 to 1.53) | 49 | 1.19 (0.81 to 1.74) | 42 | 1.02 (0.68 to 1.52) | 44 | 0.73 (0.49 to 1.08) |
| | Post-MP | T1 | 367 | reference | 283 | reference | 271 | reference | 333 | reference |
| | | T2 | 431 | 1.09 (0.95 to 1.26) | 374 | 1.17 (1.00 to 1.36) | 356 | 1.10 (0.94 to 1.29) | 351 | 1.02 (0.88 to 1.19) |
| | | T3 | 260 | 1.10 (0.93 to 1.31) | 401 | 1.11 (0.95 to 1.30) | 431 | 1.12 (0.96 to 1.31) | 374 | 1.01 (0.87 to 1.17) |
| ^ Colon | Men | T1 | 193 | reference | 232 | reference | 228 | reference | 369 | reference |
| | | T2 | 511 | 1.17 (0.99 to 1.38) | 346 | 1.25 (1.06 to 1.48)* | 326 | 1.17 (0.98 to 1.39) | 339 | 0.89 (0.77 to 1.04) |
| | | T3 | 325 | 1.40 (1.16 to 1.68)** | 451 | 1.41 (1.19 to 1.66)** | 475 | 1.45 (1.23 to 1.72)** | 321 | 0.81 (0.69 to 0.95)* |
| | Women | T1 | 307 | reference | 254 | reference | 242 | reference | 293 | reference |
| | | T2 | 352 | 1.10 (0.94 to 1.29) | 300 | 1.08 (0.92 to 1.28) | 291 | 1.06 (0.89 to 1.26) | 288 | 0.96 (0.82 to 1.14) |
| | | T3 | 230 | 1.19 (0.99 to 1.43) | 335 | 1.11 (0.94 to 1.31) | 356 | 1.13 (0.95 to 1.33) | 308 | 0.96 (0.82 to 1.14) |
| | Pre-MP | T1 | 49 | reference | 41 | reference | 46 | reference | 48 | reference |
| | | T2 | 36 | 1.05 (0.67 to 1.63) | 33 | 0.94 (0.60 to 1.50) | 30 | 0.79 (0.50 to 1.26) | 34 | 0.72 (0.46 to 1.13) |
| | | T3 | 24 | 1.12 (0.67 to 1.89) | 35 | 1.24 (0.79 to 1.96) | 33 | 1.14 (0.71 to 1.81) | 27 | 0.63 (0.39 to 1.02) |
| | Post-MP | T1 | 258 | reference | 213 | reference | 196 | reference | 245 | reference |
| | | T2 | 316 | 1.11 (0.94 to 1.32) | 267 | 1.11 (0.92 to 1.33) | 261 | 1.11 (0.92 to 1.34) | 254 | 1.01 (0.85 to 1.21) |
| | | T3 | 206 | 1.21 (0.99 to 1.47) | 300 | 1.10 (0.92 to 1.31) | 323 | 1.13 (0.94 to 1.36) | 281 | 1.02 (0.86 to 1.22) |
| ^ Rectum and recto-sigmoid junction | Men | T1 | 152 | reference | 167 | reference | 184 | reference | 232 | reference |
| | | T2 | 340 | 1.03 (0.85 to 1.25) | 239 | 1.23 (1.01 to 1.50)* | 214 | 0.98 (0.80 to 1.19) | 215 | 0.90 (0.75 to 1.09) |
| | | T3 | 193 | 1.14 (0.91 to 1.43) | 279 | 1.28 (1.05 to 1.56)* | 287 | 1.14 (0.94 to 1.39) | 238 | 0.96 (0.79 to 1.17) |
| | Women | T1 | 135 | reference | 89 | reference | 94 | reference | 105 | reference |
| | | T2 | 132 | 1.02 (0.79 to 1.30) | 128 | 1.33 (1.02 to 1.75)* | 121 | 1.17 (0.89 to 1.54) | 117 | 1.07 (0.82 to 1.39) |
| | | T3 | 65 | 0.83 (0.61 to 1.14) | 115 | 1.14 (0.86 to 1.51) | 117 | 1.06 (0.80 to 1.40) | 110 | 0.97 (0.74 to 1.28) |
| | Post-MP | T1 | 109 | reference | 70 | reference | 75 | reference | 88 | reference |
| | | T2 | 115 | 1.05 (0.80 to 1.38) | 107 | 1.35 (1.00 to 1.83) | 95 | 1.09 (0.81 to 1.48) | 97 | 1.06 (0.79 to 1.41) |
| | | T3 | 54 | 0.85 (0.60 to 1.20) | 101 | 1.16 (0.85 to 1.57) | 108 | 1.09 (0.81 to 1.48) | 93 | 0.96 (0.72 to 1.30) |
| Liver, gallbladder & bile ducts † | Men | T1 | 42 | reference | 47 | reference | 53 | reference | 87 | reference |
| | | T2 | 85 | 0.91 (0.62 to 1.32) | 69 | 1.20 (0.83 to 1.74) | 65 | 0.99 (0.68 to 1.43) | 60 | 0.75 (0.53 to 1.04) |
| | | T3 | 88 | 1.66 (1.13 to 2.45)* | 99 | 1.40 (0.98 to 2.00) | 97 | 1.20 (0.84 to 1.71) | 68 | 0.78 (0.55 to 1.10) |
| | Women | T1 | 51 | reference | 47 | reference | 40 | reference | 45 | reference |
| | | T2 | 58 | 0.97 (0.66 to 1.44) | 46 | 0.88 (0.59 to 1.33) | 50 | 1.05 (0.69 to 1.59) | 49 | 1.09 (0.73 to 1.64) |
| | | T3 | 46 | 1.17 (0.76 to 1.79) | 62 | 1.06 (0.72 to 1.56) | 65 | 1.14 (0.76 to 1.71) | 61 | 1.23 (0.83 to 1.82) |
| | Post-MP | T1 | 48 | reference | 43 | reference | 38 | reference | 42 | reference |
| | | T2 | 56 | 0.98 (0.66 to 1.45) | 43 | 0.88 (0.58 to 1.35) | 45 | 0.97 (0.63 to 1.50) | 47 | 1.12 (0.74 to 1.70) |
| | | T3 | 43 | 1.13 (0.73 to 1.76) | 61 | 1.10 (0.74 to 1.64) | 64 | 1.12 (0.74 to 1.70) | 58 | 1.23 (0.82 to 1.85) |

| **Cancer type /** ^ **subtype** | **Model** | **Cat** | **BMI** (model A) Cases | HR (95% CI) | **WHI** (model A) Cases | HR (95% CI) | **ABSI** (model B) Cases | HR (95% CI) | **HI** (model B) Cases | HR (95% CI) |
|---|---|---|---|---|---|---|---|---|---|---|
| Pancreas | Men | T1 | 64 | reference | 75 | reference | 82 | reference | 98 | reference |
| | | T2 | 146 | 1.06 (0.78 to 1.43) | 95 | 1.03 (0.76 to 1.40) | 88 | 0.83 (0.61 to 1.13) | 101 | 1.05 (0.79 to 1.40) |
| | | T3 | 91 | 1.23 (0.88 to 1.73) | 131 | 1.19 (0.89 to 1.60) | 131 | 0.99 (0.74 to 1.33) | 102 | 1.01 (0.75 to 1.36) |
| | Women | T1 | 76 | reference | 60 | reference | 52 | reference | 88 | reference |
| | | T2 | 96 | 1.17 (0.86 to 1.59) | 74 | 1.10 (0.78 to 1.54) | 83 | 1.33 (0.93 to 1.88) | 77 | 0.87 (0.64 to 1.18) |
| | | T3 | 69 | 1.45 (1.03 to 2.06)* | 107 | 1.37 (1.00 to 1.90) | 106 | 1.40 (0.99 to 1.97) | 76 | 0.78 (0.57 to 1.07) |
| | Post-MP | T1 | 72 | reference | 55 | reference | 49 | reference | 79 | reference |
| | | T2 | 87 | 1.07 (0.77 to 1.47) | 67 | 1.05 (0.74 to 1.50) | 73 | 1.19 (0.83 to 1.72) | 70 | 0.86 (0.62 to 1.19) |
| | | T3 | 60 | 1.27 (0.88 to 1.83) | 97 | 1.29 (0.92 to 1.80) | 97 | 1.28 (0.90 to 1.82) | 70 | 0.78 (0.56 to 1.08) |
| Lung (overall) | Men | T1 | 278 | reference | 182 | reference | 181 | reference | 327 | reference |
| | | T2 | 452 | 0.73 (0.63 to 0.85)** | 297 | 1.22 (1.01 to 1.47)* | 292 | 1.23 (1.02 to 1.48)* | 349 | 1.05 (0.90 to 1.23) |
| | | T3 | 277 | 0.75 (0.63 to 0.90)* | 528 | 1.56 (1.32 to 1.86)** | 534 | 1.59 (1.33 to 1.90)** | 331 | 0.88 (0.74 to 1.03) |
| | Women | T1 | 317 | reference | 169 | reference | 154 | reference | 288 | reference |
| | | T2 | 341 | 0.84 (0.72 to 0.99)* | 256 | 1.18 (0.97 to 1.43) | 261 | 1.30 (1.07 to 1.59)* | 289 | 1.04 (0.88 to 1.23) |
| | | T3 | 217 | 0.82 (0.68 to 0.99)* | 450 | 1.56 (1.30 to 1.87)** | 460 | 1.71 (1.42 to 2.06)** | 298 | 1.06 (0.90 to 1.25) |
| | Post-MP | T1 | 294 | reference | 156 | reference | 147 | reference | 267 | reference |
| | | T2 | 324 | 0.85 (0.72 to 1.00)* | 238 | 1.15 (0.94 to 1.41) | 239 | 1.21 (0.98 to 1.49) | 268 | 1.04 (0.88 to 1.23) |
| | | T3 | 203 | 0.82 (0.68 to 0.99)* | 427 | 1.53 (1.27 to 1.84)** | 435 | 1.59 (1.31 to 1.93)** | 286 | 1.08 (0.91 to 1.29) |
| ^ Lung adeno-carcinoma | Men | T1 | 96 | reference | 67 | reference | 62 | reference | 114 | reference |
| | | T2 | 151 | 0.70 (0.54 to 0.91)* | 92 | 1.04 (0.76 to 1.43) | 105 | 1.32 (0.96 to 1.82) | 117 | 0.99 (0.76 to 1.28) |
| | | T3 | 87 | 0.68 (0.50 to 0.92)* | 175 | 1.47 (1.10 to 1.96)* | 167 | 1.54 (1.13 to 2.09)* | 103 | 0.75 (0.56 to 1.00) |
| | Women | T1 | 149 | reference | 91 | reference | 80 | reference | 131 | reference |
| | | T2 | 167 | 0.91 (0.72 to 1.14) | 118 | 1.03 (0.78 to 1.36) | 125 | 1.23 (0.93 to 1.63) | 138 | 1.06 (0.84 to 1.35) |
| | | T3 | 82 | 0.70 (0.53 to 0.94)* | 189 | 1.30 (1.00 to 1.67)* | 193 | 1.49 (1.14 to 1.95)* | 129 | 1.01 (0.79 to 1.29) |
| | Post-MP | T1 | 139 | reference | 84 | reference | 76 | reference | 124 | reference |
| | | T2 | 157 | 0.89 (0.71 to 1.13) | 110 | 1.01 (0.76 to 1.35) | 115 | 1.15 (0.86 to 1.54) | 127 | 1.03 (0.80 to 1.32) |
| | | T3 | 78 | 0.70 (0.52 to 0.94)* | 180 | 1.27 (0.98 to 1.65) | 183 | 1.39 (1.05 to 1.83)* | 123 | 1.00 (0.78 to 1.29) |
| ^ Lung squamous cell carcinoma † | Men | T1 | 66 | reference | 40 | reference | 41 | reference | 83 | reference |
| | | T2 | 104 | 0.72 (0.52 to 0.98)* | 79 | 1.44 (0.99 to 2.12) | 69 | 1.23 (0.83 to 1.82) | 91 | 1.12 (0.82 to 1.51) |
| | | T3 | 86 | 0.98 (0.70 to 1.38) | 137 | 1.74 (1.21 to 2.49)* | 146 | 1.77 (1.23 to 2.55)* | 82 | 0.87 (0.63 to 1.21) |
| | Women | T1 | 46 | reference | 19 | reference | 19 | reference | 34 | reference |
| | | T2 | 47 | 0.70 (0.46 to 1.07) | 37 | 1.42 (0.81 to 2.47) | 34 | 1.28 (0.73 to 2.25) | 47 | 1.50 (0.96 to 2.33) |
| | | T3 | 36 | 0.75 (0.47 to 1.19) | 73 | 1.95 (1.17 to 3.26)* | 76 | 1.91 (1.14 to 3.20)* | 48 | 1.43 (0.91 to 2.24) |
| | Post-MP | T1 | 44 | reference | 18 | reference | 19 | reference | 33 | reference |
| | | T2 | 47 | 0.73 (0.48 to 1.12) | 36 | 1.43 (0.81 to 2.53) | 32 | 1.19 (0.67 to 2.10) | 45 | 1.47 (0.94 to 2.32) |
| | | T3 | 33 | 0.72 (0.44 to 1.17) | 70 | 1.90 (1.13 to 3.22)* | 73 | 1.78 (1.06 to 2.99)* | 46 | 1.41 (0.89 to 2.23) |
| ^ Lung small cell carcinoma † | Men | T1 | 28 | reference | 19 | reference | 22 | reference | 45 | reference |
| | | T2 | 57 | 0.94 (0.59 to 1.50) | 37 | 1.43 (0.82 to 2.50) | 40 | 1.44 (0.85 to 2.45) | 37 | 0.82 (0.52 to 1.27) |
| | | T3 | 28 | 0.74 (0.43 to 1.29) | 57 | 1.55 (0.91 to 2.63) | 51 | 1.33 (0.79 to 2.25) | 31 | 0.63 (0.39 to 1.03) |
| Kidney (overall) | Men | T1 | 72 | reference | 114 | reference | 120 | reference | 168 | reference |
| | | T2 | 201 | 1.35 (1.03 to 1.78)* | 139 | 1.03 (0.81 to 1.33) | 136 | 0.94 (0.73 to 1.20) | 139 | 0.87 (0.69 to 1.10) |
| | | T3 | 162 | 2.16 (1.61 to 2.88)** | 182 | 1.18 (0.93 to 1.50) | 179 | 1.06 (0.83 to 1.36) | 128 | 0.81 (0.64 to 1.04) |
| | Women | T1 | 55 | reference | 53 | reference | 49 | reference | 93 | reference |
| | | T2 | 88 | 1.47 (1.04 to 2.08)* | 75 | 1.33 (0.93 to 1.89) | 73 | 1.26 (0.88 to 1.82) | 72 | 0.79 (0.58 to 1.08) |
| | | T3 | 83 | 2.25 (1.57 to 3.24)** | 98 | 1.57 (1.12 to 2.21)* | 104 | 1.52 (1.07 to 2.16)* | 61 | 0.59 (0.43 to 0.82)* |
| | Post-MP | T1 | 47 | reference | 45 | reference | 43 | reference | 83 | reference |
| | | T2 | 79 | 1.46 (1.01 to 2.12)* | 66 | 1.30 (0.89 to 1.91) | 66 | 1.21 (0.82 to 1.79) | 63 | 0.77 (0.55 to 1.07) |
| | | T3 | 72 | 2.17 (1.47 to 3.21)** | 87 | 1.51 (1.05 to 2.17)* | 89 | 1.32 (0.91 to 1.92) | 52 | 0.55 (0.39 to 0.78)** |
| ^ Clear cell adeno-carcinoma † | Men | T1 | 33 | reference | 51 | reference | 54 | reference | 82 | reference |
| | | T2 | 85 | 1.20 (0.80 to 1.81) | 59 | 1.00 (0.69 to 1.46) | 64 | 1.03 (0.71 to 1.49) | 62 | 0.82 (0.59 to 1.15) |
| | | T3 | 81 | 2.19 (1.43 to 3.34)** | 89 | 1.35 (0.95 to 1.93) | 81 | 1.18 (0.82 to 1.71) | 55 | 0.75 (0.52 to 1.08) |
| | Women | T1 | 27 | reference | 23 | reference | 23 | reference | 49 | reference |
| | | T2 | 38 | 1.25 (0.75 to 2.07) | 44 | 1.86 (1.12 to 3.09)* | 35 | 1.34 (0.79 to 2.29) | 30 | 0.63 (0.40 to 0.99)* |
| | | T3 | 46 | 2.39 (1.44 to 3.99)** | 44 | 1.74 (1.04 to 2.90)* | 53 | 1.74 (1.05 to 2.90)* | 32 | 0.58 (0.37 to 0.91)* |
| | Post-MP | T1 | 22 | reference | 18 | reference | 19 | reference | 44 | reference |
| | | T2 | 35 | 1.30 (0.75 to 2.24) | 37 | 1.88 (1.07 to 3.30)* | 29 | 1.26 (0.71 to 2.27) | 24 | 0.56 (0.34 to 0.92)* |
| | | T3 | 38 | 2.22 (1.27 to 3.89)* | 40 | 1.82 (1.03 to 3.19)* | 47 | 1.67 (0.96 to 2.89) | 27 | 0.53 (0.32 to 0.86)* |

| **Cancer type /** ^ subtype | **Model** | **Cat** | **BMI** (model A) Cases | HR (95% CI) | **WHI** (model A) Cases | HR (95% CI) | **ABSI** (model B) Cases | HR (95% CI) | **HI** (model B) Cases | HR (95% CI) |
|---|---|---|---|---|---|---|---|---|---|---|
| ^ Renal cell carcinoma † | Men | T1 | 22 | reference | 42 | reference | 43 | reference | 60 | reference |
| | | T2 | 76 | 1.76 (1.09 to 2.85)* | 52 | 1.02 (0.68 to 1.54) | 49 | 0.89 (0.58 to 1.34) | 51 | 0.88 (0.60 to 1.28) |
| | | T3 | 59 | 2.76 (1.66 to 4.59)** | 63 | 1.04 (0.70 to 1.56) | 65 | 0.97 (0.65 to 1.46) | 46 | 0.81 (0.54 to 1.22) |
| | Women | T1 | 22 | reference | 24 | reference | 20 | reference | 32 | reference |
| | | T2 | 37 | 1.66 (0.97 to 2.84) | 21 | 0.79 (0.44 to 1.43) | 29 | 1.14 (0.64 to 2.03) | 36 | 1.12 (0.69 to 1.81) |
| | | T3 | 30 | 2.24 (1.25 to 4.01)* | 44 | 1.45 (0.87 to 2.41) | 40 | 1.30 (0.74 to 2.26) | 21 | 0.58 (0.33 to 1.02) |
| | Post-MP | T1 | 19 | reference | 21 | reference | 18 | reference | 28 | reference |
| | | T2 | 32 | 1.61 (0.90 to 2.87) | 20 | 0.83 (0.45 to 1.53) | 29 | 1.17 (0.65 to 2.11) | 33 | 1.15 (0.69 to 1.92) |
| | | T3 | 27 | 2.27 (1.22 to 4.24)* | 37 | 1.30 (0.76 to 2.24) | 31 | 0.99 (0.54 to 1.79) | 17 | 0.52 (0.28 to 0.95)* |
| Bladder † | Men | T1 | 93 | reference | 98 | reference | 106 | reference | 156 | reference |
| | | T2 | 215 | 1.04 (0.81 to 1.33) | 143 | 1.15 (0.89 to 1.49) | 130 | 0.93 (0.71 to 1.20) | 146 | 0.91 (0.72 to 1.15) |
| | | T3 | 142 | 1.34 (1.02 to 1.77)* | 209 | 1.37 (1.07 to 1.76)* | 214 | 1.20 (0.93 to 1.53) | 148 | 0.86 (0.68 to 1.10) |
| | Women | T1 | 35 | reference | 29 | reference | 26 | reference | 42 | reference |
| | | T2 | 42 | 0.95 (0.59 to 1.50) | 40 | 1.17 (0.73 to 1.90) | 44 | 1.36 (0.83 to 2.22) | 38 | 0.94 (0.60 to 1.46) |
| | | T3 | 39 | 1.28 (0.78 to 2.09) | 47 | 1.14 (0.71 to 1.82) | 46 | 1.09 (0.66 to 1.79) | 36 | 0.78 (0.49 to 1.23) |
| | Post-MP | T1 | 33 | reference | 28 | reference | 24 | reference | 39 | reference |
| | | T2 | 40 | 0.95 (0.59 to 1.53) | 38 | 1.13 (0.69 to 1.85) | 42 | 1.38 (0.83 to 2.29) | 36 | 0.95 (0.60 to 1.50) |
| | | T3 | 37 | 1.29 (0.77 to 2.15) | 44 | 1.08 (0.66 to 1.74) | 44 | 1.09 (0.65 to 1.81) | 35 | 0.80 (0.50 to 1.27) |
| Prostate | Men | T1 | 1,380 | reference | 1,560 | reference | 1,473 | reference | 1,650 | reference |
| | | T2 | 2,901 | 1.01 (0.94 to 1.08) | 1,909 | 1.04 (0.97 to 1.12) | 1,855 | 1.01 (0.95 to 1.09) | 1,893 | 1.04 (0.97 to 1.11) |
| | | T3 | 1,207 | 0.86 (0.80 to 0.94)** | 2,019 | 0.99 (0.92 to 1.06) | 2,160 | 1.00 (0.93 to 1.07) | 1,945 | 0.95 (0.89 to 1.02) |
| Breast (overall) | Women | T1 | 1,891 | reference | 1,680 | reference | 1,649 | reference | 1,731 | reference |
| | | T2 | 2,005 | 1.14 (1.07 to 1.22)** | 1,716 | 1.01 (0.94 to 1.08) | 1,693 | 0.98 (0.91 to 1.05) | 1,736 | 1.00 (0.93 to 1.07) |
| | | T3 | 1,282 | 1.24 (1.15 to 1.34)** | 1,782 | 1.02 (0.96 to 1.10) | 1,836 | 1.02 (0.95 to 1.09) | 1,711 | 0.96 (0.90 to 1.03) |
| Breast (pre-menopausal) | Women | T1 | 532 | reference | 449 | reference | 466 | reference | 356 | reference |
| | | T2 | 332 | 0.92 (0.80 to 1.06) | 344 | 0.95 (0.83 to 1.10) | 367 | 1.03 (0.90 to 1.18) | 362 | 1.02 (0.88 to 1.19) |
| | | T3 | 191 | 0.90 (0.75 to 1.07) | 262 | 0.97 (0.83 to 1.13) | 222 | 0.91 (0.77 to 1.07) | 337 | 1.05 (0.90 to 1.22) |
| Breast (post-menopausal) | Women | T1 | 1,359 | reference | 1,231 | reference | 1,183 | reference | 1,375 | reference |
| | | T2 | 1,673 | 1.22 (1.13 to 1.31)** | 1,372 | 1.02 (0.95 to 1.10) | 1,326 | 0.97 (0.89 to 1.05) | 1,374 | 0.99 (0.92 to 1.07) |
| | | T3 | 1,091 | 1.37 (1.25 to 1.49)** | 1,520 | 1.04 (0.97 to 1.12) | 1,614 | 1.04 (0.96 to 1.12) | 1,374 | 0.94 (0.87 to 1.02) |
| | Pre-MP | T1 | 82 | reference | 79 | reference | 85 | reference | 96 | reference |
| | | T2 | 102 | 1.63 (1.21 to 2.21)* | 89 | 1.25 (0.92 to 1.69) | 89 | 1.14 (0.84 to 1.54) | 75 | 0.78 (0.58 to 1.06) |
| | | T3 | 57 | 1.43 (1.00 to 2.05) | 73 | 1.18 (0.86 to 1.63) | 67 | 1.05 (0.75 to 1.46) | 70 | 0.84 (0.61 to 1.14) |
| | Post-MP | T1 | 1,277 | reference | 1,152 | reference | 1,098 | reference | 1,279 | reference |
| | | T2 | 1,571 | 1.20 (1.11 to 1.29)** | 1,283 | 1.01 (0.93 to 1.09) | 1,237 | 0.96 (0.88 to 1.04) | 1,299 | 1.01 (0.93 to 1.09) |
| | | T3 | 1,034 | 1.36 (1.24 to 1.48)** | 1,447 | 1.03 (0.95 to 1.12) | 1,547 | 1.03 (0.95 to 1.12) | 1,304 | 0.95 (0.88 to 1.03) |
| Ovary | Women | T1 | 210 | reference | 191 | reference | 171 | reference | 193 | reference |
| | | T2 | 241 | 1.17 (0.96 to 1.42) | 192 | 0.94 (0.77 to 1.15) | 222 | 1.18 (0.96 to 1.44) | 186 | 0.96 (0.78 to 1.18) |
| | | T3 | 143 | 1.18 (0.94 to 1.49) | 211 | 0.97 (0.79 to 1.18) | 201 | 0.96 (0.78 to 1.18) | 215 | 1.08 (0.88 to 1.31) |
| | Pre-MP | T1 | 37 | reference | 43 | reference | 34 | reference | 36 | reference |
| | | T2 | 33 | 1.30 (0.80 to 2.11) | 30 | 0.87 (0.54 to 1.39) | 43 | 1.62 (1.03 to 2.56)* | 27 | 0.79 (0.48 to 1.31) |
| | | T3 | 27 | 1.67 (0.98 to 2.84) | 24 | 0.86 (0.52 to 1.43) | 20 | 0.99 (0.56 to 1.74) | 34 | 1.10 (0.68 to 1.77) |
| | Post-MP | T1 | 173 | reference | 148 | reference | 137 | reference | 157 | reference |
| | | T2 | 208 | 1.14 (0.92 to 1.40) | 162 | 0.97 (0.77 to 1.21) | 179 | 1.10 (0.88 to 1.37) | 159 | 1.00 (0.80 to 1.24) |
| | | T3 | 116 | 1.09 (0.85 to 1.40) | 187 | 0.99 (0.80 to 1.24) | 181 | 0.94 (0.75 to 1.18) | 181 | 1.08 (0.87 to 1.34) |
| Endometrium | Women | T1 | 155 | reference | 226 | reference | 190 | reference | 231 | reference |
| | | T2 | 237 | 1.60 (1.30 to 1.96)** | 213 | 0.94 (0.78 to 1.14) | 225 | 1.04 (0.86 to 1.26) | 213 | 0.97 (0.80 to 1.17) |
| | | T3 | 307 | 3.46 (2.81 to 4.25)** | 260 | 1.10 (0.92 to 1.32) | 284 | 1.13 (0.93 to 1.36) | 255 | 1.00 (0.83 to 1.19) |
| | Pre-MP | T1 | 35 | reference | 46 | reference | 45 | reference | 33 | reference |
| | | T2 | 28 | 1.07 (0.64 to 1.79) | 30 | 0.82 (0.51 to 1.30) | 37 | 0.98 (0.63 to 1.52) | 39 | 1.22 (0.76 to 1.94) |
| | | T3 | 41 | 2.18 (1.34 to 3.56)* | 28 | 0.93 (0.58 to 1.50) | 22 | 0.71 (0.42 to 1.20) | 32 | 1.01 (0.62 to 1.66) |
| | Post-MP | T1 | 120 | reference | 180 | reference | 145 | reference | 198 | reference |
| | | T2 | 209 | 1.73 (1.38 to 2.18)** | 183 | 0.97 (0.79 to 1.19) | 188 | 1.07 (0.86 to 1.33) | 174 | 0.92 (0.75 to 1.14) |
| | | T3 | 266 | 3.79 (3.01 to 4.76)** | 232 | 1.13 (0.93 to 1.38) | 262 | 1.21 (0.98 to 1.49) | 223 | 0.99 (0.82 to 1.20) |

| Cancer type / ^ subtype | Model | Cat | BMI (model A) Cases | HR (95% CI) | WHI (model A) Cases | HR (95% CI) | ABSI (model B) Cases | HR (95% CI) | HI (model B) Cases | HR (95% CI) |
|---|---|---|---|---|---|---|---|---|---|---|
| Skin squamous cell carcinoma | Men | T1 | 251 | reference | 260 | reference | 255 | reference | 294 | reference |
| | | T2 | 484 | 0.91 (0.78 to 1.07) | 338 | 1.08 (0.92 to 1.27) | 315 | 0.95 (0.81 to 1.13) | 325 | 1.00 (0.85 to 1.17) |
| | | T3 | 218 | 0.85 (0.70 to 1.03) | 355 | 0.98 (0.83 to 1.15) | 383 | 0.93 (0.79 to 1.10) | 334 | 0.89 (0.75 to 1.05) |
| | Women | T1 | 241 | reference | 136 | reference | 153 | reference | 205 | reference |
| | | T2 | 208 | 0.76 (0.63 to 0.93)* | 197 | 1.26 (1.01 to 1.57)* | 198 | 1.12 (0.90 to 1.38) | 183 | 0.85 (0.70 to 1.04) |
| | | T3 | 112 | 0.73 (0.57 to 0.93)* | 228 | 1.29 (1.04 to 1.61)* | 210 | 1.03 (0.83 to 1.28) | 173 | 0.78 (0.63 to 0.96)* |
| | Post-MP | T1 | 224 | reference | 126 | reference | 141 | reference | 191 | reference |
| | | T2 | 195 | 0.75 (0.62 to 0.92)* | 183 | 1.24 (0.99 to 1.56) | 182 | 1.09 (0.87 to 1.36) | 172 | 0.86 (0.70 to 1.05) |
| | | T3 | 110 | 0.76 (0.59 to 0.97)* | 220 | 1.31 (1.05 to 1.63)* | 206 | 1.05 (0.84 to 1.31) | 166 | 0.79 (0.64 to 0.98)* |
| Melanoma | Men | T1 | 149 | reference | 246 | reference | 250 | reference | 257 | reference |
| | | T2 | 407 | 1.37 (1.13 to 1.65)* | 257 | 0.97 (0.82 to 1.16) | 261 | 0.93 (0.78 to 1.11) | 249 | 0.96 (0.80 to 1.14) |
| | | T3 | 194 | 1.39 (1.11 to 1.75)* | 247 | 0.93 (0.77 to 1.12) | 239 | 0.81 (0.67 to 0.98)* | 244 | 0.94 (0.78 to 1.14) |
| | Women | T1 | 297 | reference | 249 | reference | 261 | reference | 241 | reference |
| | | T2 | 296 | 1.08 (0.92 to 1.28) | 264 | 1.04 (0.88 to 1.24) | 243 | 0.89 (0.74 to 1.06) | 273 | 1.06 (0.89 to 1.27) |
| | | T3 | 143 | 0.92 (0.74 to 1.14) | 223 | 0.89 (0.74 to 1.07) | 232 | 0.84 (0.70 to 1.00) | 222 | 0.84 (0.70 to 1.01) |
| | Pre-MP | T1 | 88 | reference | 90 | reference | 103 | reference | 61 | reference |
| | | T2 | 68 | 1.25 (0.90 to 1.73) | 52 | 0.71 (0.51 to 1.01) | 38 | 0.48 (0.33 to 0.70)** | 59 | 1.00 (0.70 to 1.44) |
| | | T3 | 20 | 0.62 (0.37 to 1.03) | 34 | 0.62 (0.41 to 0.92)* | 35 | 0.66 (0.44 to 0.97)* | 56 | 1.04 (0.72 to 1.51) |
| | Post-MP | T1 | 209 | reference | 159 | reference | 158 | reference | 180 | reference |
| | | T2 | 228 | 1.04 (0.86 to 1.27) | 212 | 1.21 (0.99 to 1.49) | 205 | 1.11 (0.90 to 1.36) | 214 | 1.10 (0.90 to 1.34) |
| | | T3 | 123 | 0.99 (0.78 to 1.26) | 189 | 1.01 (0.82 to 1.26) | 197 | 0.95 (0.77 to 1.18) | 166 | 0.79 (0.64 to 0.98)* |
| Brain | Men | T1 | 76 | reference | 80 | reference | 79 | reference | 91 | reference |
| | | T2 | 119 | 0.77 (0.57 to 1.03) | 101 | 1.19 (0.88 to 1.60) | 86 | 0.99 (0.73 to 1.36) | 79 | 0.84 (0.62 to 1.14) |
| | | T3 | 71 | 0.88 (0.63 to 1.24) | 85 | 0.93 (0.68 to 1.28) | 101 | 1.05 (0.77 to 1.44) | 96 | 0.92 (0.68 to 1.26) |
| | Women | T1 | 58 | reference | 41 | reference | 42 | reference | 52 | reference |
| | | T2 | 75 | 1.32 (0.92 to 1.88) | 61 | 1.37 (0.92 to 2.03) | 58 | 1.26 (0.84 to 1.88) | 66 | 1.26 (0.88 to 1.82) |
| | | T3 | 34 | 1.03 (0.66 to 1.61) | 65 | 1.34 (0.90 to 1.99) | 67 | 1.32 (0.88 to 1.96) | 49 | 0.91 (0.61 to 1.36) |
| | Post-MP | T1 | 49 | reference | 31 | reference | 33 | reference | 44 | reference |
| | | T2 | 63 | 1.25 (0.85 to 1.83) | 53 | 1.48 (0.95 to 2.30) | 48 | 1.24 (0.79 to 1.93) | 55 | 1.24 (0.83 to 1.85) |
| | | T3 | 30 | 1.06 (0.66 to 1.72) | 58 | 1.41 (0.91 to 2.20) | 61 | 1.32 (0.86 to 2.05) | 43 | 0.92 (0.60 to 1.41) |
| Thyroid | Men | T1 | 15 | reference | 12 | reference | 14 | reference | 17 | reference |
| | | T2 | 26 | 0.81 (0.42 to 1.55) | 21 | 1.71 (0.84 to 3.51) | 19 | 1.27 (0.63 to 2.58) | 16 | 0.89 (0.44 to 1.78) |
| | | T3 | 13 | 0.71 (0.32 to 1.55) | 21 | 1.62 (0.78 to 3.38) | 21 | 1.34 (0.65 to 2.76) | 21 | 1.09 (0.54 to 2.18) |
| | Women | T1 | 45 | reference | 46 | reference | 51 | reference | 54 | reference |
| | | T2 | 61 | 1.47 (0.99 to 2.20) | 57 | 1.24 (0.84 to 1.83) | 48 | 0.89 (0.60 to 1.33) | 45 | 0.83 (0.56 to 1.24) |
| | | T3 | 39 | 1.56 (0.99 to 2.47) | 42 | 0.90 (0.59 to 1.38) | 46 | 0.81 (0.53 to 1.22) | 46 | 0.84 (0.56 to 1.25) |
| | Post-MP | T1 | 29 | reference | 29 | reference | 35 | reference | 40 | reference |
| | | T2 | 45 | 1.49 (0.92 to 2.41) | 43 | 1.36 (0.85 to 2.18) | 35 | 0.85 (0.53 to 1.37) | 34 | 0.85 (0.54 to 1.35) |
| | | T3 | 32 | 1.80 (1.05 to 3.09)* | 34 | 1.00 (0.60 to 1.65) | 36 | 0.74 (0.46 to 1.20) | 32 | 0.74 (0.46 to 1.19) |
| Leukaemia | Men | T1 | 84 | reference | 120 | reference | 107 | reference | 125 | reference |
| | | T2 | 201 | 1.18 (0.91 to 1.52) | 111 | 0.80 (0.62 to 1.04) | 125 | 0.94 (0.72 to 1.22) | 97 | 0.76 (0.58 to 0.99)* |
| | | T3 | 93 | 1.06 (0.78 to 1.45) | 147 | 0.94 (0.73 to 1.21) | 146 | 0.89 (0.68 to 1.16) | 156 | 1.18 (0.92 to 1.53) |
| | Women | T1 | 82 | reference | 74 | reference | 65 | reference | 81 | reference |
| | | T2 | 94 | 1.11 (0.82 to 1.51) | 80 | 0.99 (0.72 to 1.36) | 87 | 1.16 (0.84 to 1.61) | 77 | 0.92 (0.67 to 1.26) |
| | | T3 | 58 | 1.13 (0.79 to 1.62) | 80 | 0.91 (0.66 to 1.26) | 82 | 0.96 (0.68 to 1.34) | 76 | 0.85 (0.62 to 1.17) |
| | Post-MP | T1 | 67 | reference | 64 | reference | 53 | reference | 69 | reference |
| | | T2 | 89 | 1.21 (0.87 to 1.68) | 66 | 0.90 (0.64 to 1.28) | 75 | 1.16 (0.81 to 1.65) | 67 | 0.93 (0.66 to 1.30) |
| | | T3 | 48 | 1.07 (0.72 to 1.58) | 74 | 0.90 (0.64 to 1.26) | 76 | 0.98 (0.68 to 1.40) | 68 | 0.87 (0.62 to 1.22) |
| Multiple myeloma | Men | T1 | 53 | reference | 58 | reference | 58 | reference | 68 | reference |
| | | T2 | 101 | 0.92 (0.66 to 1.29) | 75 | 1.13 (0.80 to 1.60) | 76 | 1.08 (0.76 to 1.54) | 72 | 1.00 (0.71 to 1.40) |
| | | T3 | 53 | 0.98 (0.66 to 1.47) | 74 | 1.01 (0.70 to 1.44) | 73 | 0.88 (0.61 to 1.26) | 67 | 0.85 (0.59 to 1.22) |
| | Women | T1 | 62 | reference | 54 | reference | 54 | reference | 63 | reference |
| | | T2 | 54 | 0.80 (0.55 to 1.17) | 51 | 0.88 (0.60 to 1.30) | 49 | 0.78 (0.53 to 1.15) | 59 | 0.94 (0.66 to 1.34) |
| | | T3 | 58 | 1.41 (0.96 to 2.08) | 69 | 1.09 (0.75 to 1.56) | 71 | 0.97 (0.67 to 1.40) | 52 | 0.75 (0.51 to 1.08) |
| | Post-MP | T1 | 49 | reference | 45 | reference | 43 | reference | 54 | reference |
| | | T2 | 49 | 0.88 (0.59 to 1.33) | 42 | 0.84 (0.55 to 1.27) | 42 | 0.79 (0.51 to 1.21) | 49 | 0.92 (0.62 to 1.36) |
| | | T3 | 53 | 1.57 (1.03 to 2.39)* | 64 | 1.11 (0.76 to 1.64) | 66 | 1.00 (0.67 to 1.49) | 48 | 0.79 (0.53 to 1.17) |

| Cancer type / ^ subtype | Model | Cat | **BMI** (model A) Cases | HR (95% CI) | **WHI** (model A) Cases | HR (95% CI) | **ABSI** (model B) Cases | HR (95% CI) | **HI** (model B) Cases | HR (95% CI) |
|---|---|---|---|---|---|---|---|---|---|---|
| Non-Hodgkin lymphoma | Men | T1 | 140 | reference | 175 | reference | 176 | reference | 198 | reference |
| | | T2 | 282 | 0.99 (0.81 to 1.22) | 189 | 0.94 (0.77 to 1.16) | 191 | 0.91 (0.74 to 1.13) | 194 | 0.96 (0.78 to 1.17) |
| | | T3 | 149 | 1.03 (0.81 to 1.32) | 207 | 0.92 (0.75 to 1.13) | 204 | 0.83 (0.67 to 1.03) | 179 | 0.83 (0.67 to 1.04) |
| | Women | T1 | 183 | reference | 152 | reference | 142 | reference | 167 | reference |
| | | T2 | 194 | 0.98 (0.80 to 1.21) | 158 | 0.96 (0.76 to 1.19) | 152 | 0.95 (0.76 to 1.20) | 161 | 0.95 (0.76 to 1.18) |
| | | T3 | 113 | 0.94 (0.73 to 1.21) | 180 | 0.99 (0.80 to 1.24) | 196 | 1.08 (0.86 to 1.35) | 162 | 0.90 (0.72 to 1.12) |
| | Post-MP | T1 | 158 | reference | 125 | reference | 120 | reference | 142 | reference |
| | | T2 | 163 | 0.92 (0.73 to 1.15) | 142 | 1.00 (0.79 to 1.27) | 129 | 0.90 (0.70 to 1.16) | 138 | 0.95 (0.75 to 1.20) |
| | | T3 | 104 | 0.98 (0.75 to 1.28) | 158 | 0.98 (0.77 to 1.25) | 176 | 1.03 (0.81 to 1.31) | 145 | 0.92 (0.72 to 1.16) |

† – cancers with less than 20 cases in women pre-menopausal at baseline, for which models were not adjusted for menopausal status; **ABSI** – a body shape index; **BMI** – body mass index; **CI** – confidence interval; **HI** – hip index; **HR** – hazard ratio; **MP** – menopause; **SCC** – squamous cell carcinoma; **WHI** – waist-to-hip index; **T1-T3** – sex-specific tertiles for ABSI (cut-off points: 78.1 and 81.5 for men, 71.5 and 75.7 for women), HI (cut-off points: 59.4 and 61.1 for men, 63.3 and 65.3 for women) and WHI (cut-off points: 3.98 and 4.17 for men, 3.47 and 3.69 for women) or BMI categories according to the World Health Organisation: normal weight (T1) BMI ≥ 18.5 to < 25 kg/m$^2$, overweight (T2) BMI ≥ 25 to < 30 kg/m$^2$, obese (T3) BMI ≥ 30 to < 45 kg/m$^2$. Hazard ratios (95% confidence intervals) were obtained from delayed entry Cox proportional hazards models stratified by age at baseline and region of the assessment centre. **Model A** – included BMI and WHI with adjustment variables. **Model B** – included BMI, ABSI and HI with adjustment variables (HR estimates for BMI in Model A and Model B were similar). **Men** – models were adjusted for height, weight change during last year preceding baseline, Townsend deprivation index, smoking status, alcohol consumption, physical activity, consumption of fresh fruit and vegetables, processed meat and red meat, family history of cancer and, for skin SCC and melanoma, sun-exposure-related factors. **Women** – models included adjustment variables as for men, with the addition of menopausal status (except for cancers marked with †), use of hormone replacement therapy, ever use of oral contraceptives and age at last live birth (with “no live births” as one of the categories). **Pre-MP** – for the models, this applies to the sub-group of women pre-menopausal at baseline, with adjustment as in models for women. **Pre-menopausal** – applies to breast cancers diagnosed below 55 years of age in women pre-menopausal at baseline. **Post-MP** – for the models, this applies to the sub-group of women post-menopausal at baseline, with adjustment as in models for women. **Post-menopausal** – applies to breast cancers diagnosed at age 55 years or older, irrespective of menopausal status at baseline, i.e. women diagnosed with post-menopausal breast cancer, could be pre- or post-menopausal at baseline; Cancer types and subtypes are defined in Supplementary Methods according to the 10th edition of the International Statistical Classification of Diseases (ICD10). Obesity-related cancers included oesophageal adenocarcinoma, cancers of the gastric cardia, colon, rectum and rectosigmoid junction, liver, gallbladder and bile ducts, pancreas, kidney, postmenopausal breast, ovary, endometrium, thyroid and multiple myeloma. Non-obesity-related cancers included the remaining cancers. The table includes cancer types and analysis groups (men, women, pre-menopausal or post-menopausal women), for which at least 10 cases were available in each body-shape index tertile or BMI category. * p-value < 0.05; ** p-value < 0.001.

## Supplementary Table S4 Traditional obesity indices in relation to cancer risk

| **Cancer type /** ^ **subtype** | **Sex** | **Model** | Cases | **BMI** (model A) ‡ (per 5 $kg/m^2$) HR (95% CI) | **WHI** (model A) ‡ (per one SD) HR (95% CI) | **BMI** (model B) ‡ (per 5 $kg/m^2$) HR (95% CI) | **ABSI** (model B) ‡ (per one SD) HR (95% CI) | **HI** (model B) ‡ (per one SD) HR (95% CI) |
|---|---|---|---|---|---|---|---|---|
| Head and neck (overall) | Men | Indi | 412 | 0.92 (0.81 to 1.04) | 1.04 (0.94 to 1.15) | 0.92 (0.81 to 1.04) | 0.98 (0.88 to 1.09) | 0.92 (0.83 to 1.02) |
| | | Join | | 0.84 (0.72 to 0.99)* | 1.13 (1.00 to 1.27) | 0.80 (0.59 to 1.10) | 1.27 (1.01 to 1.60)* | 0.89 (0.72 to 1.09) |
| | Women | Indi | 164 | 0.84 (0.70 to 1.01) | 1.21 (1.04 to 1.41)* | 0.84 (0.70 to 1.01) | 0.99 (0.84 to 1.16) | 0.81 (0.68 to 0.97)* |
| | | Join | | 0.72 (0.59 to 0.89)* | 1.36 (1.15 to 1.61)** | 0.72 (0.45 to 1.17) | 1.68 (1.22 to 2.31)* | 0.70 (0.48 to 1.03) |
| Oesophagus (overall) † | Men | Indi | 373 | 1.35 (1.20 to 1.52)** | 1.34 (1.22 to 1.48)** | 1.35 (1.20 to 1.52)** | 1.36 (1.23 to 1.51)** | 1.22 (1.11 to 1.35)** |
| | | Join | | 1.16 (1.01 to 1.35)* | 1.26 (1.12 to 1.42)** | 0.92 (0.67 to 1.26) | 1.58 (1.25 to 2.00)** | 0.91 (0.75 to 1.11) |
| | Women | Indi | 115 | 0.72 (0.57 to 0.92)* | 1.05 (0.87 to 1.27) | 0.72 (0.57 to 0.92)* | 0.86 (0.70 to 1.05) | 0.75 (0.60 to 0.94)* |
| | | Join | | 0.65 (0.50 to 0.85)* | 1.22 (1.00 to 1.50) | 0.57 (0.31 to 1.04) | 1.45 (0.98 to 2.17) | 0.89 (0.56 to 1.42) |
| ^ Oesophageal AC † | Men | Indi | 283 | 1.45 (1.26 to 1.66)** | 1.43 (1.28 to 1.59)** | 1.45 (1.26 to 1.66)** | 1.45 (1.29 to 1.62)** | 1.26 (1.12 to 1.40)** |
| | | Join | | 1.21 (1.03 to 1.43)* | 1.32 (1.16 to 1.51)** | 1.04 (0.72 to 1.49) | 1.66 (1.27 to 2.17)** | 0.82 (0.66 to 1.03) |
| | Women | Indi | 47 | 1.12 (0.82 to 1.53) | 0.98 (0.72 to 1.32) | 1.12 (0.82 to 1.53) | 1.06 (0.78 to 1.43) | 1.13 (0.84 to 1.51) |
| | | Join | | 1.16 (0.82 to 1.63) | 0.92 (0.66 to 1.28) | 1.11 (0.45 to 2.71) | 0.86 (0.47 to 1.59) | 1.16 (0.58 to 2.32) |
| ^ Oesophageal SCC † | Men | Indi | 46 | 0.81 (0.55 to 1.20) | 0.92 (0.68 to 1.24) | 0.81 (0.55 to 1.20) | 0.94 (0.68 to 1.28) | 0.99 (0.73 to 1.34) |
| | | Join | | 0.81 (0.51 to 1.29) | 1.01 (0.70 to 1.46) | 0.42 (0.18 to 1.01) | 1.33 (0.67 to 2.65) | 1.40 (0.89 to 2.22) |
| | Women | Indi | 60 | 0.43 (0.30 to 0.64)** | 1.01 (0.77 to 1.31) | 0.43 (0.30 to 0.64)** | 0.66 (0.49 to 0.90)* | 0.49 (0.35 to 0.69)** |
| | | Join | | 0.35 (0.23 to 0.54)** | 1.42 (1.07 to 1.87)* | 0.28 (0.12 to 0.69)* | 2.00 (1.14 to 3.49)* | 0.77 (0.39 to 1.51) |
| Stomach (overall) † | Men | Indi | 220 | 1.25 (1.07 to 1.47)* | 1.36 (1.19 to 1.54)** | 1.25 (1.07 to 1.47)* | 1.27 (1.11 to 1.46)** | 1.08 (0.95 to 1.24) |
| | | Join | | 1.03 (0.85 to 1.25) | 1.34 (1.15 to 1.56)** | 1.10 (0.73 to 1.67) | 1.59 (1.17 to 2.16)* | 0.70 (0.54 to 0.91)* |
| | Women | Indi | 97 | 1.06 (0.85 to 1.32) | 1.09 (0.89 to 1.34) | 1.06 (0.85 to 1.32) | 1.06 (0.86 to 1.31) | 0.99 (0.80 to 1.22) |
| | | Join | | 1.02 (0.80 to 1.30) | 1.09 (0.87 to 1.36) | 1.32 (0.73 to 2.40) | 1.08 (0.71 to 1.65) | 0.73 (0.46 to 1.16) |
| ^ Gastric cardia † | Men | Indi | 107 | 1.42 (1.13 to 1.78)* | 1.52 (1.26 to 1.84)** | 1.42 (1.13 to 1.78)* | 1.42 (1.17 to 1.71)** | 1.16 (0.96 to 1.40) |
| | | Join | | 1.10 (0.83 to 1.46) | 1.46 (1.15 to 1.83)* | 1.22 (0.68 to 2.21) | 1.76 (1.13 to 2.73)* | 0.64 (0.44 to 0.92)* |
| | Women | Indi | 26 | 0.95 (0.62 to 1.47) | 0.97 (0.65 to 1.44) | 0.95 (0.62 to 1.47) | 0.90 (0.60 to 1.36) | 0.87 (0.57 to 1.32) |
| | | Join | | 0.96 (0.59 to 1.54) | 0.98 (0.63 to 1.53) | 1.67 (0.55 to 5.08) | 0.82 (0.36 to 1.87) | 0.66 (0.28 to 1.59) |
| ^ Gastric non-cardia † | Men | Indi | 113 | 1.11 (0.88 to 1.40) | 1.23 (1.02 to 1.48)* | 1.11 (0.88 to 1.40) | 1.15 (0.95 to 1.39) | 1.01 (0.84 to 1.23) |
| | | Join | | 0.95 (0.72 to 1.26) | 1.25 (1.01 to 1.56)* | 0.99 (0.55 to 1.78) | 1.44 (0.93 to 2.23) | 0.76 (0.53 to 1.10) |
| | Women | Indi | 71 | 1.10 (0.85 to 1.41) | 1.14 (0.90 to 1.45) | 1.10 (0.85 to 1.41) | 1.13 (0.89 to 1.44) | 1.03 (0.81 to 1.32) |
| | | Join | | 1.04 (0.79 to 1.38) | 1.12 (0.87 to 1.46) | 1.18 (0.58 to 2.39) | 1.21 (0.74 to 1.98) | 0.77 (0.44 to 1.34) |
| Colorectal (overall) | Men | Indi | 1,714 | 1.14 (1.08 to 1.22)** | 1.19 (1.13 to 1.25)** | 1.14 (1.08 to 1.22)** | 1.17 (1.11 to 1.23)** | 1.08 (1.02 to 1.13)* |
| | | Join | | 1.02 (0.95 to 1.10) | 1.18 (1.11 to 1.25)** | 0.93 (0.80 to 1.09) | 1.36 (1.22 to 1.53)** | 0.88 (0.80 to 0.97)* |
| | Women | Indi | 1,221 | 1.06 (0.99 to 1.13) | 1.09 (1.03 to 1.15)* | 1.06 (0.99 to 1.13) | 1.09 (1.02 to 1.15)* | 1.04 (0.98 to 1.11) |
| | | Join | | 1.02 (0.96 to 1.10) | 1.08 (1.01 to 1.15)* | 0.97 (0.81 to 1.15) | 1.16 (1.03 to 1.31)* | 0.95 (0.83 to 1.09) |
| ^ Colon | Men | Indi | 1,029 | 1.20 (1.11 to 1.29)** | 1.23 (1.16 to 1.31)** | 1.20 (1.11 to 1.29)** | 1.22 (1.14 to 1.30)** | 1.11 (1.04 to 1.18)* |
| | | Join | | 1.06 (0.97 to 1.16) | 1.20 (1.12 to 1.29)** | 0.97 (0.80 to 1.18) | 1.41 (1.22 to 1.63)** | 0.86 (0.76 to 0.97)* |
| | Women | Indi | 889 | 1.08 (1.01 to 1.17)* | 1.10 (1.03 to 1.18)* | 1.08 (1.01 to 1.17)* | 1.11 (1.04 to 1.19)* | 1.07 (1.00 to 1.15) |
| | | Join | | 1.05 (0.97 to 1.13) | 1.08 (1.00 to 1.16)* | 0.94 (0.77 to 1.16) | 1.19 (1.03 to 1.37)* | 0.98 (0.84 to 1.15) |
| ^ Rectum and rectosigmoid junction | Men | Indi | 685 | 1.06 (0.96 to 1.17) | 1.12 (1.04 to 1.21)* | 1.06 (0.96 to 1.17) | 1.10 (1.02 to 1.19)* | 1.03 (0.95 to 1.12) |
| | | Join | | 0.97 (0.86 to 1.10) | 1.14 (1.03 to 1.25)* | 0.87 (0.68 to 1.11) | 1.30 (1.09 to 1.56)* | 0.92 (0.79 to 1.08) |
| | Women | Indi | 332 | 0.99 (0.88 to 1.12) | 1.05 (0.94 to 1.18) | 0.99 (0.88 to 1.12) | 1.01 (0.90 to 1.14) | 0.97 (0.86 to 1.09) |
| | | Join | | 0.96 (0.84 to 1.10) | 1.07 (0.95 to 1.21) | 1.03 (0.73 to 1.45) | 1.09 (0.86 to 1.38) | 0.89 (0.68 to 1.15) |
| Liver, gallbladder and bile ducts † | Men | Indi | 215 | 1.39 (1.19 to 1.63)** | 1.31 (1.15 to 1.51)** | 1.39 (1.19 to 1.63)** | 1.32 (1.15 to 1.50)** | 1.17 (1.03 to 1.34)* |
| | | Join | | 1.25 (1.03 to 1.51)* | 1.19 (1.01 to 1.40)* | 1.51 (1.00 to 2.27)* | 1.22 (0.90 to 1.66) | 0.75 (0.58 to 0.97)* |
| | Women | Indi | 155 | 1.08 (0.91 to 1.28) | 1.08 (0.92 to 1.27) | 1.08 (0.91 to 1.28) | 1.12 (0.95 to 1.32) | 1.09 (0.93 to 1.28) |
| | | Join | | 1.05 (0.87 to 1.27) | 1.06 (0.89 to 1.26) | 0.81 (0.50 to 1.31) | 1.22 (0.88 to 1.70) | 1.12 (0.77 to 1.62) |
| Pancreas | Men | Indi | 301 | 1.10 (0.95 to 1.27) | 1.08 (0.96 to 1.21) | 1.10 (0.95 to 1.27) | 1.09 (0.97 to 1.23) | 1.06 (0.94 to 1.19) |
| | | Join | | 1.07 (0.90 to 1.28) | 1.04 (0.90 to 1.21) | 1.05 (0.73 to 1.51) | 1.09 (0.83 to 1.43) | 0.96 (0.76 to 1.20) |
| | Women | Indi | 241 | 1.18 (1.03 to 1.35)* | 1.20 (1.05 to 1.36)* | 1.18 (1.03 to 1.35)* | 1.20 (1.06 to 1.37)* | 1.11 (0.97 to 1.26) |
| | | Join | | 1.11 (0.96 to 1.29) | 1.15 (1.00 to 1.32)* | 1.21 (0.83 to 1.77) | 1.24 (0.95 to 1.61) | 0.80 (0.59 to 1.07) |
| Lung (overall) | Men | Indi | 1007 | 0.86 (0.79 to 0.93)** | 1.08 (1.02 to 1.16)* | 0.86 (0.79 to 0.93)** | 0.98 (0.92 to 1.04) | 0.89 (0.84 to 0.96)** |
| | | Join | | 0.74 (0.67 to 0.81)** | 1.24 (1.15 to 1.34)** | 0.59 (0.48 to 0.71)** | 1.57 (1.36 to 1.81)** | 0.90 (0.80 to 1.02) |
| | Women | Indi | 875 | 0.92 (0.85 to 0.99)* | 1.14 (1.07 to 1.22)** | 0.92 (0.85 to 0.99)* | 1.04 (0.97 to 1.12) | 0.94 (0.87 to 1.01) |
| *(continues)* | | Join | | 0.84 (0.77 to 0.91)** | 1.22 (1.13 to 1.31)** | 0.60 (0.49 to 0.74)** | 1.56 (1.36 to 1.79)** | 1.01 (0.86 to 1.18) |

| Cancer type / ^ subtype | Sex | Model | Cases | **BMI** (model A) ‡ (per 5 kg/m²) HR (95% CI) | **WHI** (model A) ‡ (per one SD) HR (95% CI) | **BMI** (model B) ‡ (per 5 kg/m²) HR (95% CI) | **ABSI** (model B) ‡ (per one SD) HR (95% CI) | **HI** (model B) ‡ (per one SD) HR (95% CI) |
|---|---|---|---|---|---|---|---|---|
| ^ Lung AC | Men | Indi | 334 | 0.86 (0.75 to 0.99)* | 1.05 (0.94 to 1.17) | 0.86 (0.75 to 0.99)* | 0.95 (0.85 to 1.07) | 0.88 (0.78 to 0.99)* |
| | | Join | | 0.77 (0.65 to 0.92)* | 1.17 (1.03 to 1.34)* | 0.73 (0.52 to 1.03) | 1.34 (1.04 to 1.73)* | 0.86 (0.69 to 1.08) |
| | Women | Indi | 398 | 0.85 (0.75 to 0.95)* | 1.07 (0.97 to 1.18) | 0.85 (0.75 to 0.95)* | 0.95 (0.86 to 1.06) | 0.87 (0.78 to 0.97)* |
| | | Join | | 0.79 (0.69 to 0.90)** | 1.17 (1.04 to 1.30)* | 0.63 (0.46 to 0.86)* | 1.39 (1.13 to 1.71)* | 0.99 (0.77 to 1.26) |
| ^ Lung SCC † | Men | Indi | 256 | 0.93 (0.80 to 1.09) | 1.19 (1.05 to 1.35)* | 0.93 (0.80 to 1.09) | 1.06 (0.94 to 1.21) | 0.93 (0.82 to 1.06) |
| | | Join | | 0.77 (0.63 to 0.92)* | 1.33 (1.15 to 1.53)** | 0.59 (0.40 to 0.88)* | 1.80 (1.36 to 2.39)** | 0.84 (0.66 to 1.07) |
| | Women | Indi | 129 | 0.84 (0.69 to 1.02) | 1.18 (0.99 to 1.40) | 0.84 (0.69 to 1.02) | 1.05 (0.88 to 1.25) | 0.92 (0.77 to 1.10) |
| | | Join | | 0.74 (0.60 to 0.93)* | 1.30 (1.08 to 1.56)* | 0.35 (0.21 to 0.58)** | 2.02 (1.45 to 2.81)** | 1.26 (0.85 to 1.88) |
| ^ Lung small-cell carcinoma † | Men | Indi | 113 | 0.84 (0.66 to 1.06) | 1.04 (0.86 to 1.26) | 0.84 (0.66 to 1.06) | 0.90 (0.74 to 1.10) | 0.82 (0.67 to 1.01) |
| | | Join | | 0.74 (0.56 to 1.00)* | 1.18 (0.94 to 1.48) | 0.92 (0.51 to 1.64) | 1.20 (0.78 to 1.84) | 0.76 (0.52 to 1.10) |
| | Women | Indi | 96 | 1.21 (0.98 to 1.48) | 1.35 (1.11 to 1.64)* | 1.21 (0.98 to 1.48) | 1.37 (1.12 to 1.66)* | 1.20 (0.99 to 1.46) |
| | | Join | | 1.08 (0.86 to 1.36) | 1.31 (1.06 to 1.62)* | 0.61 (0.34 to 1.11) | 1.91 (1.28 to 2.86)* | 1.08 (0.69 to 1.71) |
| Kidney (overall) | Men | Indi | 435 | 1.41 (1.26 to 1.58)** | 1.25 (1.14 to 1.38)** | 1.41 (1.26 to 1.58)** | 1.30 (1.19 to 1.43)** | 1.22 (1.11 to 1.33)** |
| | | Join | | 1.33 (1.16 to 1.52)** | 1.09 (0.97 to 1.23) | 1.58 (1.17 to 2.12)* | 1.08 (0.86 to 1.35) | 0.83 (0.69 to 1.00) |
| | Women | Indi | 226 | 1.39 (1.22 to 1.58)** | 1.42 (1.26 to 1.61)** | 1.39 (1.22 to 1.58)** | 1.41 (1.24 to 1.60)** | 1.20 (1.06 to 1.37)* |
| | | Join | | 1.24 (1.08 to 1.44)* | 1.31 (1.14 to 1.51)** | 1.76 (1.21 to 2.54)* | 1.35 (1.03 to 1.76)* | 0.58 (0.44 to 0.78)** |
| ^ Clear-cell AC † | Men | Indi | 199 | 1.48 (1.26 to 1.74)** | 1.29 (1.12 to 1.49)** | 1.48 (1.26 to 1.74)** | 1.35 (1.18 to 1.56)** | 1.25 (1.09 to 1.43)* |
| | | Join | | 1.39 (1.13 to 1.69)* | 1.11 (0.93 to 1.32) | 1.68 (1.09 to 2.58)* | 1.09 (0.79 to 1.51) | 0.81 (0.61 to 1.06) |
| | Women | Indi | 111 | 1.46 (1.22 to 1.76)** | 1.54 (1.29 to 1.83)** | 1.46 (1.22 to 1.76)** | 1.52 (1.27 to 1.82)** | 1.24 (1.04 to 1.48)* |
| | | Join | | 1.28 (1.04 to 1.57)* | 1.41 (1.16 to 1.71)** | 1.72 (1.03 to 2.88)* | 1.56 (1.08 to 2.26)* | 0.55 (0.37 to 0.81)* |
| ^ Renal-cell carcinoma † | Men | Indi | 157 | 1.46 (1.21 to 1.75)** | 1.30 (1.11 to 1.53)* | 1.46 (1.21 to 1.75)** | 1.35 (1.16 to 1.58)** | 1.25 (1.08 to 1.46)* |
| | | Join | | 1.35 (1.08 to 1.69)* | 1.12 (0.93 to 1.37) | 1.52 (0.93 to 2.51) | 1.13 (0.78 to 1.64) | 0.84 (0.62 to 1.15) |
| | Women | Indi | 89 | 1.37 (1.11 to 1.68)* | 1.31 (1.07 to 1.60)* | 1.37 (1.11 to 1.68)* | 1.33 (1.09 to 1.63)* | 1.21 (0.99 to 1.48) |
| | | Join | | 1.27 (1.01 to 1.60)* | 1.20 (0.96 to 1.50) | 1.86 (1.02 to 3.41)* | 1.12 (0.73 to 1.71) | 0.65 (0.41 to 1.03) |
| Bladder † | Men | Indi | 450 | 1.10 (0.98 to 1.24) | 1.17 (1.06 to 1.28)* | 1.10 (0.98 to 1.24) | 1.13 (1.02 to 1.25)* | 1.04 (0.95 to 1.15) |
| | | Join | | 0.99 (0.85 to 1.14) | 1.17 (1.04 to 1.32)* | 0.93 (0.69 to 1.26) | 1.33 (1.06 to 1.65)* | 0.88 (0.73 to 1.06) |
| | Women | Indi | 116 | 1.13 (0.93 to 1.37) | 1.18 (0.98 to 1.41) | 1.13 (0.93 to 1.37) | 1.18 (0.98 to 1.42) | 1.07 (0.89 to 1.29) |
| | | Join | | 1.06 (0.86 to 1.32) | 1.15 (0.95 to 1.40) | 1.06 (0.62 to 1.80) | 1.32 (0.91 to 1.92) | 0.82 (0.54 to 1.24) |
| Prostate | Men | Indi | 5,488 | 0.92 (0.89 to 0.96)** | 0.98 (0.95 to 1.00) | 0.92 (0.89 to 0.96)** | 0.94 (0.92 to 0.97)** | 0.94 (0.91 to 0.97)** |
| | | Join | | 0.91 (0.87 to 0.95)** | 1.02 (0.98 to 1.05) | 0.94 (0.86 to 1.03) | 1.01 (0.95 to 1.08) | 0.97 (0.91 to 1.02) |
| Breast (overall) | Women | Indi | 5,178 | 1.11 (1.08 to 1.15)** | 1.05 (1.02 to 1.08)** | 1.11 (1.08 to 1.15)** | 1.09 (1.06 to 1.12)** | 1.09 (1.06 to 1.12)** |
| | | Join | | 1.11 (1.07 to 1.14)** | 1.01 (0.98 to 1.04) | 1.15 (1.06 to 1.26)** | 1.00 (0.94 to 1.06) | 0.96 (0.90 to 1.03) |
| ^ Breast (pre-MP) | | Indi | 1,055 | 0.98 (0.92 to 1.05) | 0.95 (0.89 to 1.01) | 0.98 (0.92 to 1.05) | 0.96 (0.90 to 1.03) | 1.00 (0.93 to 1.06) |
| | | Join | | 1.01 (0.94 to 1.09) | 0.94 (0.88 to 1.01) | 1.01 (0.83 to 1.23) | 0.90 (0.78 to 1.04) | 1.07 (0.92 to 1.25) |
| ^ Breast (post-MP) | | Indi | 4,123 | 1.15 (1.12 to 1.19)** | 1.08 (1.05 to 1.11)** | 1.15 (1.12 to 1.19)** | 1.13 (1.09 to 1.17)** | 1.12 (1.08 to 1.15)** |
| | | Join | | 1.14 (1.10 to 1.18)** | 1.03 (0.99 to 1.06) | 1.20 (1.09 to 1.32)** | 1.02 (0.95 to 1.09) | 0.94 (0.87 to 1.01) |
| Ovary | Women | Indi | 594 | 1.06 (0.97 to 1.17) | 1.01 (0.93 to 1.10) | 1.06 (0.97 to 1.17) | 1.05 (0.96 to 1.14) | 1.06 (0.98 to 1.16) |
| | | Join | | 1.07 (0.97 to 1.19) | 0.98 (0.89 to 1.07) | 1.03 (0.80 to 1.33) | 0.98 (0.83 to 1.17) | 1.05 (0.86 to 1.27) |
| Endometrium | Women | Indi | 699 | 1.78 (1.66 to 1.90)** | 1.35 (1.26 to 1.45)** | 1.78 (1.66 to 1.90)** | 1.70 (1.59 to 1.82)** | 1.63 (1.53 to 1.74)** |
| | | Join | | 1.73 (1.61 to 1.86)** | 1.08 (1.00 to 1.17) | 1.63 (1.33 to 2.00)** | 1.15 (0.99 to 1.33) | 0.97 (0.82 to 1.13) |
| Skin SCC | Men | Indi | 953 | 0.91 (0.83 to 0.99)* | 0.95 (0.88 to 1.01) | 0.91 (0.83 to 0.99)* | 0.91 (0.84 to 0.97)* | 0.91 (0.84 to 0.98)* |
| | | Join | | 0.92 (0.83 to 1.02) | 0.98 (0.90 to 1.07) | 1.11 (0.90 to 1.38) | 0.89 (0.77 to 1.04) | 0.92 (0.81 to 1.06) |
| | Women | Indi | 561 | 0.84 (0.76 to 0.93)* | 1.03 (0.95 to 1.13) | 0.84 (0.76 to 0.93)* | 0.90 (0.82 to 0.99)* | 0.82 (0.75 to 0.91)** |
| | | Join | | 0.79 (0.71 to 0.89)** | 1.12 (1.02 to 1.23)* | 0.93 (0.71 to 1.21) | 1.15 (0.96 to 1.38) | 0.79 (0.64 to 0.97)* |
| Melanoma | Men | Indi | 750 | 1.18 (1.08 to 1.30)** | 1.00 (0.93 to 1.09) | 1.18 (1.08 to 1.30)** | 1.07 (0.99 to 1.16) | 1.11 (1.03 to 1.20)* |
| | | Join | | 1.28 (1.14 to 1.43)** | 0.89 (0.81 to 0.98)* | 1.54 (1.22 to 1.94)** | 0.77 (0.65 to 0.92)* | 1.02 (0.88 to 1.18) |
| | Women | Indi | 736 | 0.97 (0.89 to 1.06) | 0.96 (0.88 to 1.03) | 0.97 (0.89 to 1.06) | 0.93 (0.86 to 1.01) | 0.94 (0.87 to 1.03) |
| | | Join | | 0.99 (0.90 to 1.09) | 0.96 (0.88 to 1.05) | 1.28 (1.02 to 1.62)* | 0.85 (0.72 to 1.00)* | 0.87 (0.72 to 1.04) |
| Brain | Men | Indi | 266 | 0.91 (0.78 to 1.07) | 0.93 (0.82 to 1.06) | 0.91 (0.78 to 1.07) | 0.90 (0.79 to 1.03) | 0.91 (0.80 to 1.04) |
| | | Join | | 0.94 (0.77 to 1.14) | 0.96 (0.82 to 1.13) | 1.14 (0.77 to 1.69) | 0.87 (0.65 to 1.16) | 0.93 (0.72 to 1.20) |
| | Women | Indi | 167 | 0.99 (0.83 to 1.18) | 1.13 (0.97 to 1.32) | 0.99 (0.83 to 1.18) | 1.04 (0.88 to 1.22) | 0.94 (0.79 to 1.11) |
| | | Join | | 0.92 (0.76 to 1.12) | 1.16 (0.98 to 1.38) | 1.09 (0.68 to 1.75) | 1.22 (0.88 to 1.68) | 0.75 (0.52 to 1.08) |

| Cancer type / ^ subtype | Sex | Model | Cases | BMI (model A) ǂ (per 5 kg/m²) HR (95% CI) | WHI (model A) ǂ (per one SD) HR (95% CI) | BMI (model B) ǂ (per 5 kg/m²) HR (95% CI) | ABSI (model B) ǂ (per one SD) HR (95% CI) | HI (model B) ǂ (per one SD) HR (95% CI) |
|---|---|---|---|---|---|---|---|---|
| Thyroid | Men | Indi | 54 | 0.87 (0.62 to 1.24) | 1.10 (0.83 to 1.45) | 0.87 (0.62 to 1.24) | 1.01 (0.76 to 1.34) | 0.92 (0.69 to 1.24) |
| | | Join | | 0.74 (0.49 to 1.13) | 1.25 (0.93 to 1.67) | 0.50 (0.20 to 1.22) | 1.73 (0.91 to 3.30) | 0.96 (0.55 to 1.70) |
| | Women | Indi | 145 | 1.14 (0.95 to 1.35) | 1.05 (0.89 to 1.24) | 1.14 (0.95 to 1.35) | 1.09 (0.92 to 1.29) | 1.08 (0.91 to 1.28) |
| | | Join | | 1.14 (0.94 to 1.38) | 1.00 (0.83 to 1.20) | 1.42 (0.86 to 2.34) | 0.92 (0.65 to 1.30) | 0.86 (0.58 to 1.27) |
| Leukemia | Men | Indi | 378 | 0.98 (0.85 to 1.12) | 0.95 (0.85 to 1.05) | 0.98 (0.85 to 1.12) | 0.95 (0.85 to 1.06) | 0.98 (0.88 to 1.09) |
| | | Join | | 1.02 (0.87 to 1.20) | 0.94 (0.82 to 1.07) | 1.14 (0.82 to 1.59) | 0.86 (0.67 to 1.09) | 1.01 (0.82 to 1.25) |
| | Women | Indi | 234 | 1.12 (0.98 to 1.29) | 1.02 (0.89 to 1.16) | 1.12 (0.98 to 1.29) | 1.07 (0.93 to 1.22) | 1.08 (0.95 to 1.23) |
| | | Join | | 1.14 (0.98 to 1.33) | 0.97 (0.83 to 1.12) | 1.39 (0.94 to 2.06) | 0.88 (0.67 to 1.16) | 0.90 (0.66 to 1.22) |
| Multiple myeloma | Men | Indi | 207 | 0.90 (0.75 to 1.08) | 0.98 (0.85 to 1.14) | 0.90 (0.75 to 1.08) | 0.90 (0.78 to 1.05) | 0.85 (0.73 to 1.00)* |
| | | Join | | 0.87 (0.69 to 1.09) | 1.05 (0.88 to 1.25) | 1.24 (0.79 to 1.94) | 0.95 (0.69 to 1.33) | 0.77 (0.58 to 1.02) |
| | Women | Indi | 174 | 1.13 (0.96 to 1.33) | 1.09 (0.94 to 1.27) | 1.13 (0.96 to 1.33) | 1.11 (0.95 to 1.29) | 1.05 (0.90 to 1.23) |
| | | Join | | 1.10 (0.92 to 1.32) | 1.05 (0.89 to 1.25) | 1.45 (0.93 to 2.27) | 1.01 (0.74 to 1.38) | 0.76 (0.54 to 1.07) |
| Non-Hodgkin lymphoma | Men | Indi | 571 | 1.03 (0.93 to 1.15) | 1.00 (0.92 to 1.09) | 1.03 (0.93 to 1.15) | 0.98 (0.89 to 1.07) | 0.97 (0.88 to 1.06) |
| | | Join | | 1.05 (0.92 to 1.19) | 0.98 (0.88 to 1.09) | 1.46 (1.12 to 1.90)* | 0.85 (0.70 to 1.03) | 0.85 (0.72 to 1.01) |
| | Women | Indi | 490 | 0.99 (0.90 to 1.10) | 1.03 (0.94 to 1.13) | 0.99 (0.90 to 1.10) | 1.02 (0.93 to 1.12) | 0.99 (0.90 to 1.09) |
| | | Join | | 0.97 (0.87 to 1.09) | 1.05 (0.95 to 1.16) | 0.92 (0.69 to 1.21) | 1.10 (0.91 to 1.33) | 0.99 (0.80 to 1.23) |

**†** – cancers with less than 20 cases in women pre-menopausal at baseline, for which models were not adjusted for menopausal status; **AC** – adenocarcinoma; **BMI** – body mass index; **CI** – confidence interval; **HC** – hip circumference; **HR** – hazard ratio; **MP** – menopausal; **SCC** – squamous cell carcinoma; **SD** – standard deviation; **WC** – waist circumference; **WHR** – waist-to-hip ratio. **HRs (95% CI)** were obtained from delayed entry Cox proportional hazards models stratified by age at baseline and region of the assessment centre. **Indi** – models included individually each of BMI, WHR, WC or HC with adjustment variables (note that the values for BMI in columns "Model A" and "Model B" are the same, as this is the same model); **Join** – models included combinations of obesity indices with adjustment variables (**ǂ** – combinations of obesity indices in Model A and Model B were applied only to models in row "Join"; **Model A** – included BMI and WHR with adjustment variables. **Model B** – included BMI, WC and HC with adjustment variables; **Men** – models were adjusted for height, weight change during last year preceding baseline, Townsend deprivation index, smoking status, alcohol consumption, physical activity, consumption of fresh fruit and vegetables, processed meat and red meat, family history of cancer, and, for skin SCC and melanoma, sun-exposure-related factors. **Women** – models included adjustment variables as for men, with the addition of menopausal status (except for cancers marked with **†**), use of hormone replacement therapy, ever use of oral contraceptives and age at last live birth (with "no live births" as one of the categories); **pre-MP** – breast cancer diagnosed below 55 years of age in women pre-menopausal at baseline; **post-MP** – breast cancer diagnosed at age 55 years or older, irrespective of menopausal status at baseline. Cancer types and subtypes are defined in Supplementary Methods according to the 10th edition of the International Statistical Classification of Diseases (ICD10). Obesity-related cancers included oesophageal adenocarcinoma, cancers of the gastric cardia, colon, rectum and rectosigmoid junction, liver, gallbladder and bile ducts, pancreas, kidney, postmenopausal breast, ovary, endometrium, thyroid and multiple myeloma. Non-obesity-related cancers included the remaining cancers. * p-value < 0.05; ** p-value < 0.001.

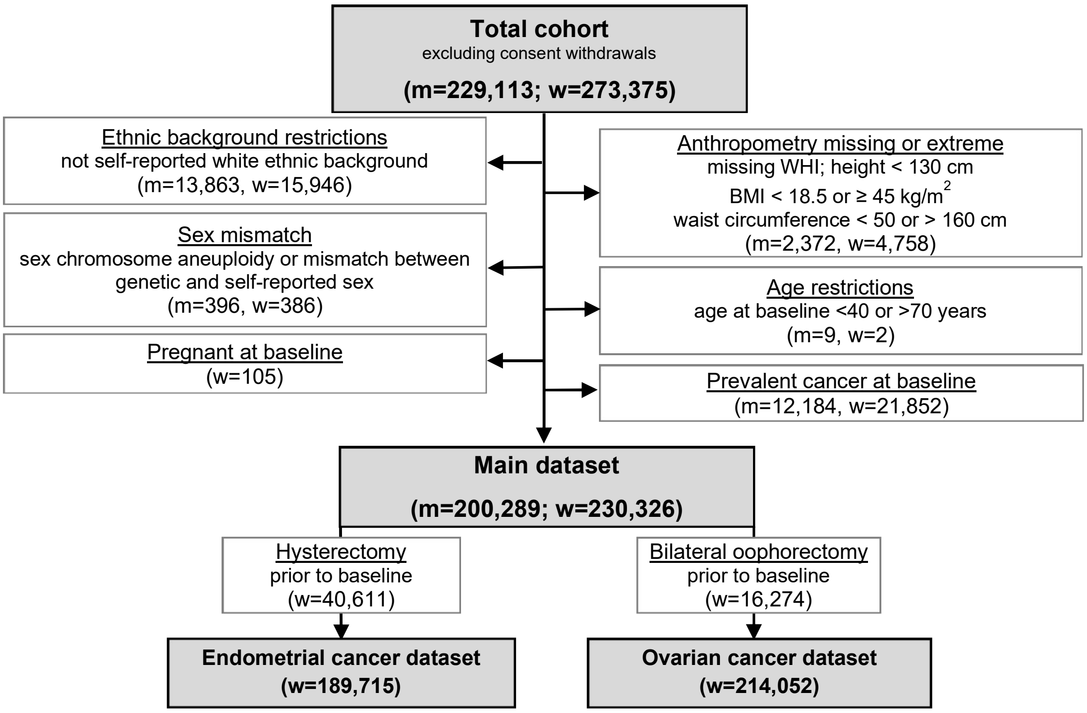


**Supplementary Figure S1 Flow diagram of UK Biobank participants included in the study**

**BMI** – body mass index; **HRT** – hormone replacement therapy; **m** – number of men; **w** – number of women. Supplementary Methods include details of the definition of variables. The exclusion criteria were applied sequentially in the order indicated by the position corresponding horizontal arrow, such that each excluded individual was counted only once.

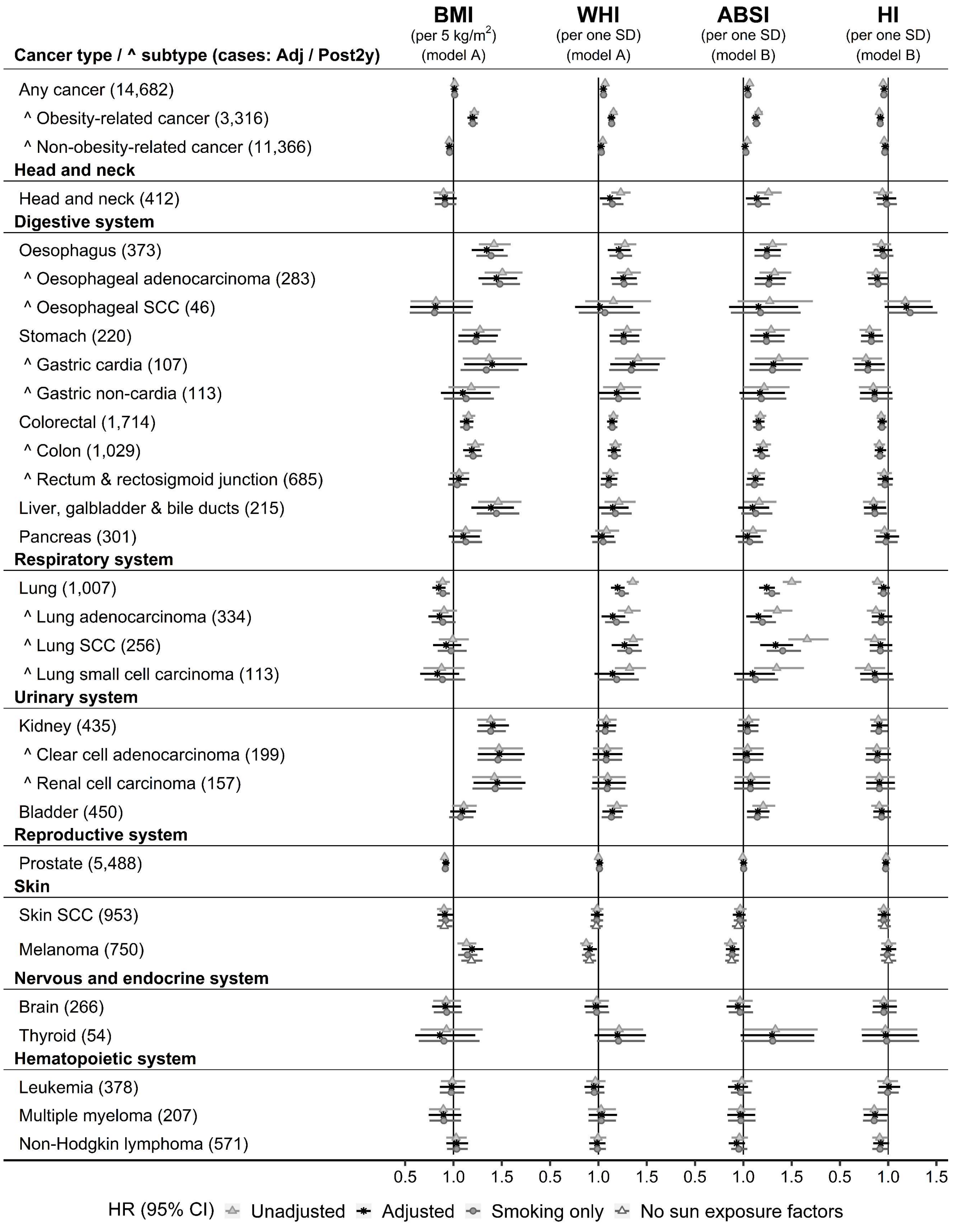


**Supplementary Figure S2 Sensitivity analyses with alternative adjustments in men**

**ABSI** – a body shape index; **BMI** – body mass index; **CI** – confidence interval; **HI** – hip index; **HR** – hazard ratio; **SCC** – squamous cell carcinoma; **SD** – standard deviation; **WHI** – waist-to-hip index. HRs (95% CI) were obtained from delayed entry Cox proportional hazards models stratified by age at baseline and region of the assessment centre; **Model A** – included BMI and WHI. **Model B** – included BMI, ABSI and HI with adjustment variables (HR estimates for BMI in Model A and Model

B were similar); **Unadjusted** – models including one of the two combinations of BMI and body-shape indices without adjustment variables, but retaining the stratification; **Adjusted** – models including one of the two combinations of BMI and body-shape indices and adjustment for height, weight change during last year preceding baseline, Townsend deprivation index, smoking status, alcohol consumption, physical activity, family history of cancer, consumption of fresh fruit and vegetables, processed meat intake and red meat intake, family history of cancer and, for skin SCC and melanoma, sun-exposure-related factors; **Smoking only** – models including one of the two combinations of BMI and body-shape indices with adjustment only for smoking status, but retaining the stratification; **No sun exposure factors** – as the fully adjusted models, but with sun-exposure-related risk factors omitted from the adjustment variables (only for skin SCC and melanoma). Cancer types and subtypes are defined in Supplementary Methods according to the 10$^{th}$ edition of the International Statistical Classification of Diseases (ICD10). Obesity-related cancers included oesophageal adenocarcinoma, cancers of the gastric cardia, colon, rectum and rectosigmoid junction, liver, gallbladder and bile ducts, pancreas, kidney, thyroid and multiple myeloma. Non-obesity-related cancers included the remaining cancers.

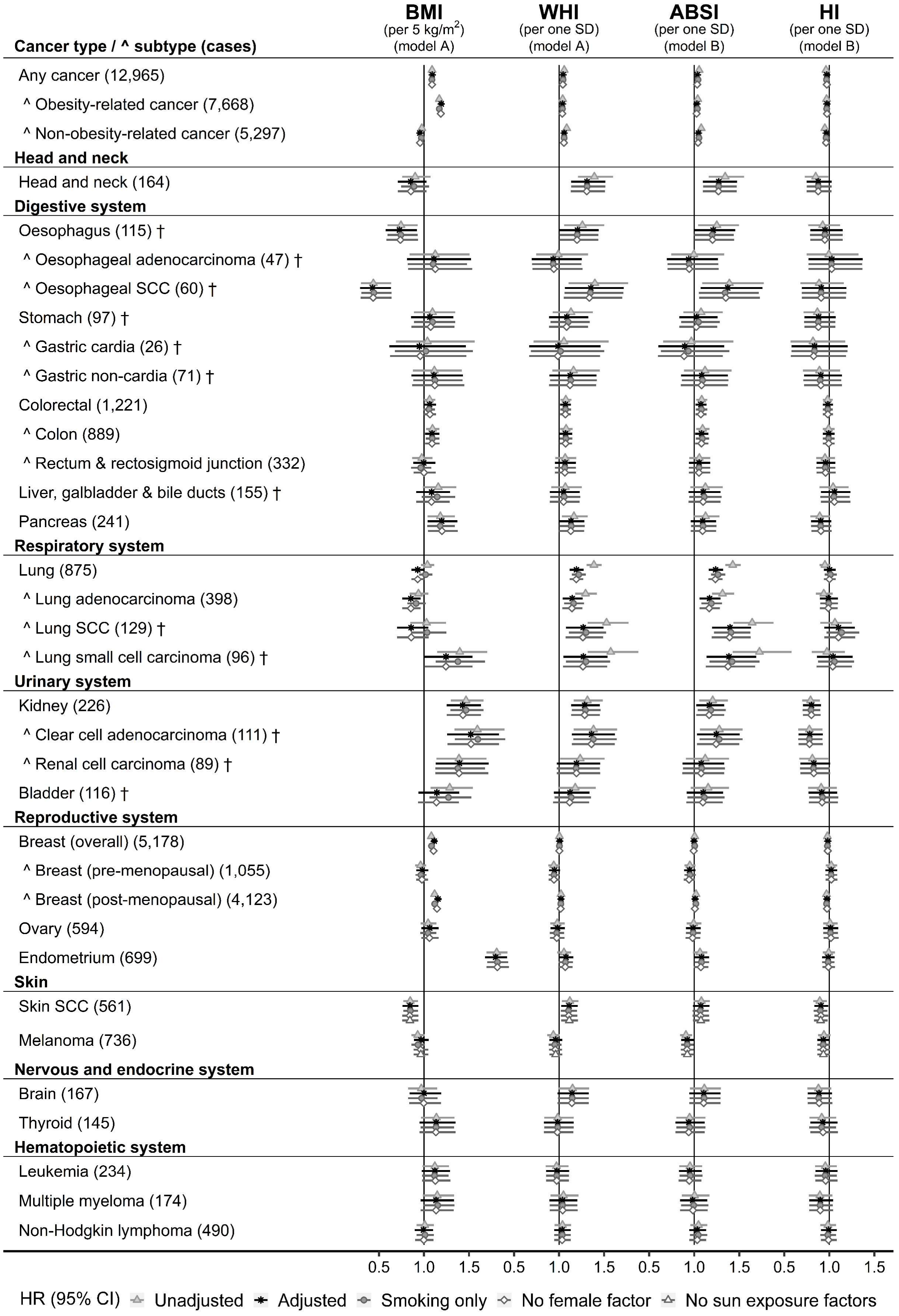


**Supplementary Figure S3 Sensitivity analyses with alternative adjustments in women**

**†** – cancers with less than 20 cases in women pre-menopausal at baseline, for which models were not adjusted for menopausal status; **ABSI** – a body shape index; **BMI** – body mass index; **CI** – confidence interval; **HI** – hip index; **HR** – hazard ratio; **SCC** – squamous cell carcinoma; **SD** – standard deviation; **WHI** – waist-to-hip index. **HRs (95% CI)** were obtained from delayed entry Cox proportional hazards models stratified by age at baseline and region of the assessment centre; **Model A** – included BMI and WHI. **Model B** – included BMI, ABSI and HI with adjustment variables (HR estimates for BMI in Model A and Model B were similar); **Unadjusted** – models including one of the two combinations of BMI and body-shape indices without adjustment variables, but retaining the stratification; **Adjusted** – models including one of the two combinations of BMI and body-shape indices and adjustment for height, weight change during last year preceding baseline, Townsend deprivation index, smoking status, alcohol consumption, physical activity, consumption of fresh fruit and vegetables, processed meat and red meat, family history of cancer, menopausal status (except for cancers marked with †), use of hormone replacement therapy, ever use of oral contraceptives, age at last live birth (with “no live births” as one of the categories) and, for skin SCC and melanoma, sun-exposure-related factors; **Smoking only** – models including one of the two combinations of BMI and body-shape indices with adjustment only for smoking status, but retaining the stratification; **No female factors** – as the fully adjusted models, but with female-specific risk factors omitted from the adjustment variables; **No sun exposure factors** – as the fully adjusted models, but with sun-exposure-related risk factors omitted from the adjustment variables (only for skin SCC and melanoma); Cancer types and subtypes are defined in Supplementary Methods according to the 10$^{th}$ edition of the International Statistical Classification of Diseases (ICD10) . Obesity-related cancers included oesophageal adenocarcinoma, cancers of the gastric cardia, colon, rectum and rectosigmoid junction, liver, gallbladder and bile ducts, pancreas, kidney, postmenopausal breast, ovary, endometrium, thyroid and multiple myeloma. Non-obesity-related cancers included the remaining cancers.

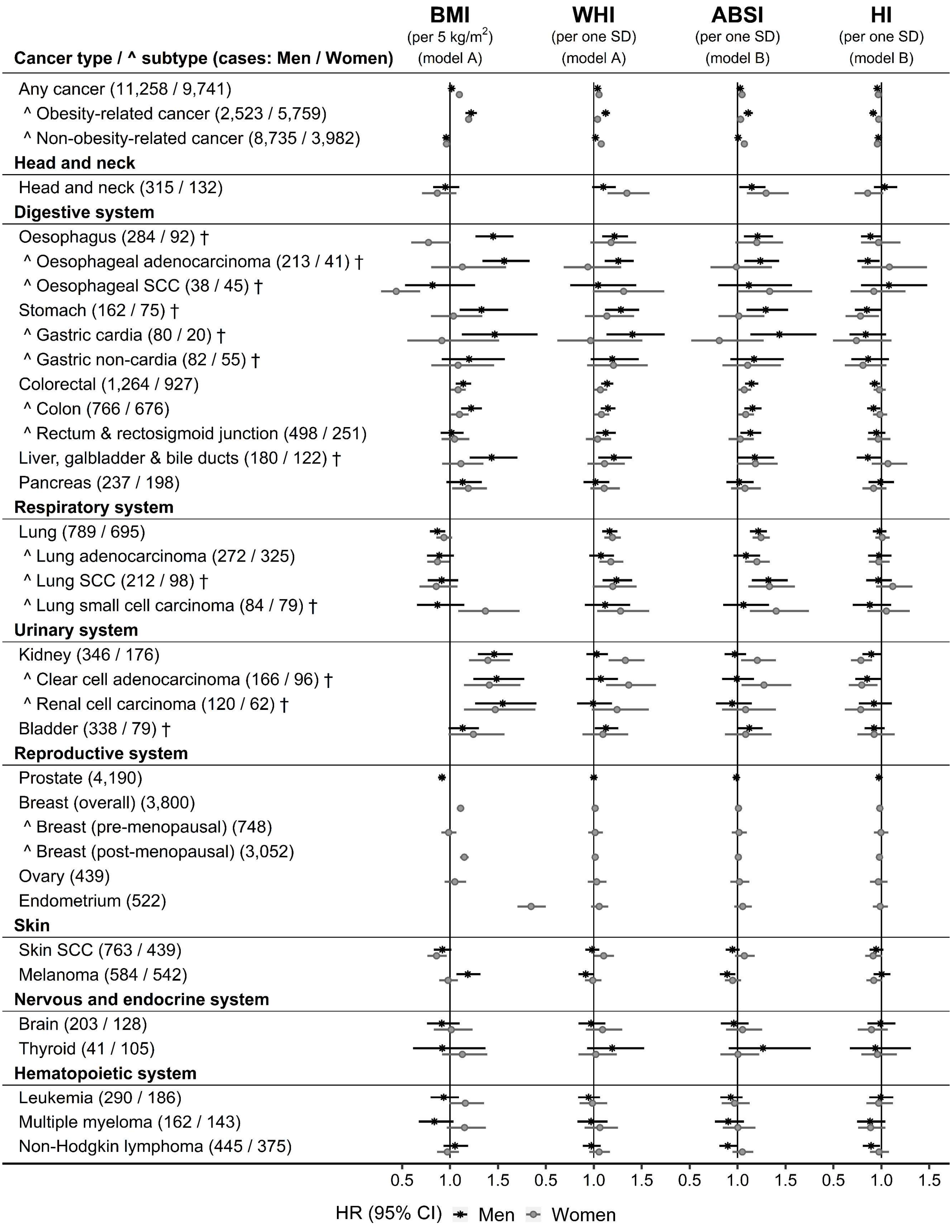


**Supplementary Figure S4 Sensitivity analyses excluding participants with less than two years of follow-up**

**†** – cancers with less than 20 cases in women pre-menopausal at baseline, for which models were not adjusted for menopausal status; **ABSI** – a body shape index; **BMI** – body mass index; **CI** – confidence interval; **HI** – hip index; **HR** – hazard ratio; **SCC** – squamous cell carcinoma; **SD** – standard deviation; **WHI** – waist-to-hip index. **HRs (95% CI)** were obtained from delayed entry Cox

proportional hazards models stratified by age at baseline and region of the assessment centre. **Model A** – included BMI and WHI with adjustment variables. **Model B** – included BMI, ABSI and HI with adjustment variables (HR estimates for BMI in Model A and Model B were similar). **Men** – models were adjusted for height, weight change during last year preceding baseline, Townsend deprivation index, smoking status, alcohol consumption, physical activity, consumption of fresh fruit and vegetables, processed meat and red meat, family history of cancer and, for skin SCC and melanoma, sun-exposure-related factors. **Women** – models included adjustment variables as for men, with the addition of menopausal status (except for cancers marked with †), use of hormone replacement therapy, ever use of oral contraceptives and age at last live birth (with "no live births" as one of the categories). Cancer types and subtypes are defined in Supplementary Methods according to the 10$^{th}$ edition of the International Statistical Classification of Diseases (ICD10). Obesity-related cancers included oesophageal adenocarcinoma, cancers of the gastric cardia, colon, rectum and rectosigmoid junction, liver, gallbladder and bile ducts, pancreas, kidney, postmenopausal breast, ovary, endometrium, thyroid and multiple myeloma. Non-obesity-related cancers included the remaining cancers.

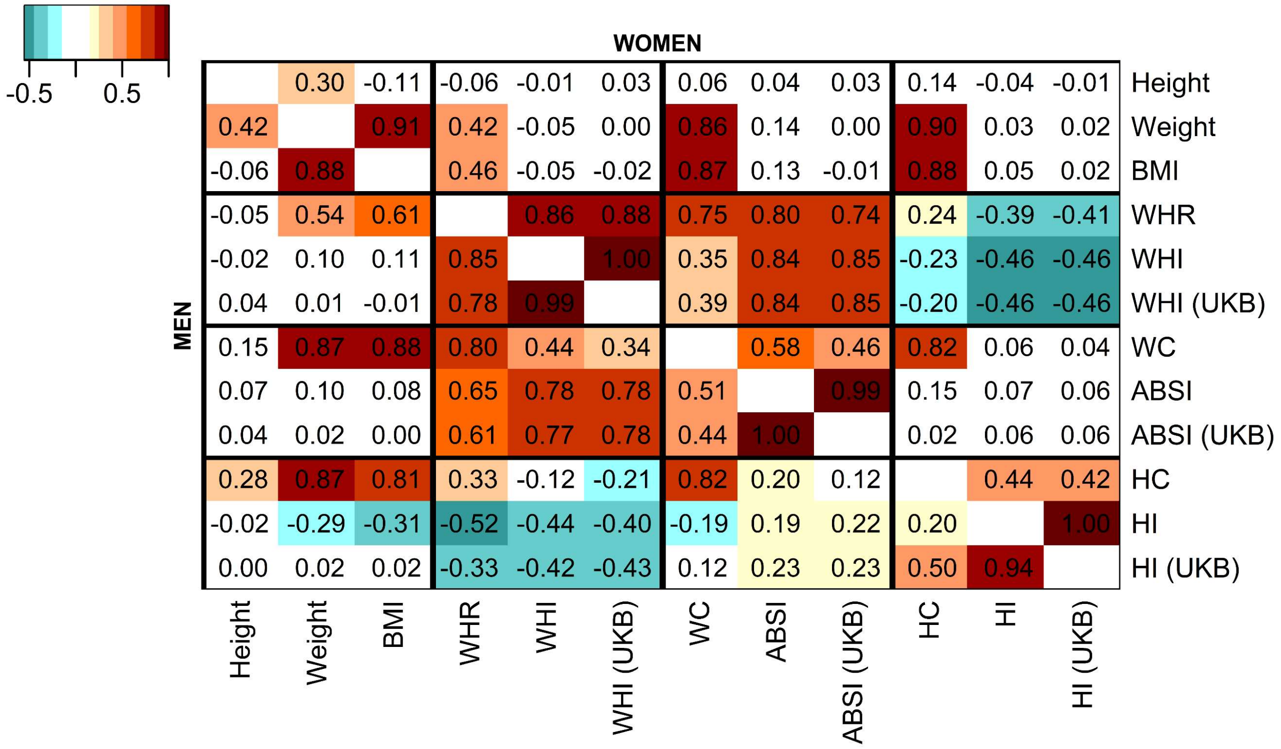


**Supplementary Figure S5 Correlation between anthropometric indices**

**ABSI** – a body shape index calculated with published regression coefficients, derived from the National Health and Nutrition Examination Survey (NHANES) (5); **ABSI (UKB)** – ABSI calculated with regression coefficients derived from UK Biobank data in the current study; **BMI** – body mass index; **HC** – hip circumference; **HI** – hip index calculated with published regression coefficients, derived from NHANES (6); **HI (UKB)** – HI calculated with regression coefficients derived from UK Biobank data in the current study; **WC** – waist circumference; **WHI** – waist-to-hip index calculated with coefficients derived from UK Biobank data and rounded to simple fractions; **WHI (UKB)** – WHI calculated with the exact regression coefficients derived from UK Biobank data in the current study; **WHR** – waist-to-hip ratio. **Men** – bottom left half of panel. **Women** – top right half of panel. **Cells** – show partial Pearson correlation coefficients with adjustment for age at baseline and region of the assessment centre.

**Note:** In women, hazard ratio (HR) estimates based on ABSI and HI calculated with regression coefficients from NHANES were almost identical to HR estimates based on ABSI (UKB) and HI (UKB) calculated with regression coefficients derived from UK Biobank data. In men, there was similarly no material difference between HR estimates based on ABSI and HI calculated with regression coefficients from NHANES and from UK Biobank, despite some weak inverse association between BMI and HI calculated with regression coefficients from NHANES.
